# Preclinical Efficacy of Recombinant Human Platelet-Derived Growth Factor-BB in Experimental Diabetic Wound Healing: A Systematic Review and Meta-Analysis of Animal Studies

**DOI:** 10.3390/ph19091497

**Published:** 2026-09-21

**Authors:** José Luis Muñoz-Carrillo, Oscar Gutiérrez-Coronado, Edrei Lopez-Hernandez, Rosalinda Gutiérrez-Hernández, Yael Sabbagh-Permuth, Ana Karola Zamora-Aguilar, Natalie Rodríguez-Cortés, Francisca Chávez-Ruvalcaba, Paola Trinidad Villalobos-Gutiérrez, María Isabel Chávez-Ruvalcaba

**Affiliations:** 1 Laboratorio de Inmunología, Centro Universitario de los Lagos, Universidad de Guadalajara, Enrique Díaz de León 1144, Paseos de La Montaña, Lagos de Moreno 47463, Jalisco, Mexico; paola.villalobos2452@academicos.udg.mx; 2 Centro de Investigación e Innovación, Global University, Av Independencia 2134, Trojes de Alonso, Aguascalientes 20116, Aguascalientes, Mexico; 3 Unidad de Ciencias de la Salud, Facultad de Medicina Campus Xalapa, Universidad Veracruzana, Médicos y Odontólogos S/N, Unidad del Bosque, Xalapa de Enriquez 91010, Veracruz, Mexico; edreihernandez777@gmail.com; 4 Licenciatura en Nutrición, Unidad Académica de Enfermería, Universidad Autónoma de Zacatecas, Carretera Zacatecas-Guadalajara Km. 6 Ejido La Escondida, Zacatecas 98160, Zacatecas, Mexico; rosalinda@uaz.edu.mx (R.G.-H.); charuva@uaz.edu.mx (F.C.-R.); 5 Escuela de Medicina, Facultad de Ciencias de la Salud, Universidad Anáhuac México, Av Universidad Anáhuac 46, Lomas Anáhuac, Huixquilucan 52786, Estado de México, Mexico; ysabbagh@anahuac.mx (Y.S.-P.); anakarola@live.com.mx (A.K.Z.-A.); 6 Facultad de Medicina, Universidad El Bosque, Bogotá 110121, PC, Colombia; natalirodriguez@unbosque.edu.co; 7 Laboratorio de Inmunoparasitología, Unidad Académica de Ciencias Biologicas, Universidad Autónoma de Zacatecas, Avenida Preparatoria S/N, Hidráulica, Zacatecas 98060, Zacatecas, Mexico; iasruv9si@uaz.edu.mx

**Keywords:** platelet-derived growth factor, recombinant human PDGF-BB, becaplermin, wound healing, diabetic foot, diabetes mellitus, experimental animal models, drug delivery systems, systematic review, meta-analysis

## Abstract

**Background/Objectives**: Recombinant human platelet-derived growth factor-BB (rhPDGF-BB) is intended to restore reparative signaling in diabetic wounds, yet its efficacy and the contribution of delivery systems remain uncertain. This systematic review evaluated its preclinical efficacy, mechanistic responses, safety, risk of bias, certainty, and attribution of effects. **Methods**: Following PRISMA 2020 and an OSF-registered protocol, PubMed, Embase, Scopus, Web of Science, and ScienceDirect were searched from inception to 29 July 2026 for controlled in vivo diabetic wound studies. Compatible outcomes were pooled using random-effects models with restricted maximum-likelihood estimation and Hartung–Knapp adjustment. Non-poolable findings were synthesized using attribution-specific effect direction maps. Risk of bias and certainty were assessed with SYRCLE and preclinical GRADE, respectively. **Results**: Twenty-nine studies were included from 3844 records. For percentage wound closure at days 10–12, three studies comprising 113 animals yielded a favorable but imprecise pooled estimate (mean difference: 12.94 percentage points; 95% CI: −0.34 to 26.23; *p* = 0.052; I^2^ = 54.6%). Two studies comprising 45 animals suggested a non-significant 4.28-day reduction in time to complete closure (95% CI: −27.98 to 19.42). In the exploratory effect direction synthesis, directly attributable macroscopic healing showed a favorable direction in 17 of 22 studies and was the only domain meeting the prespecified 70% concordance threshold; four of these studies had confirmed unit-of-analysis concerns, and other reparative domains were heterogeneous. Formulation-level comparisons met the same exploratory threshold across four reparative domains, but the isolated contribution of rhPDGF-BB could not be determined. Safety reporting was sparse. Certainty for the primary outcome was very low. **Conclusions**: Directly attributable comparisons showed a predominantly favorable study-level pattern for macroscopic wound healing in the exploratory synthesis, but the pooled effects were imprecise and inconclusive. Formulation-level comparisons suggested broader reparative activity, although the independent contribution of rhPDGF-BB could not be isolated. Better controlled and clinically representative studies are required before confident translation.

## 1. Introduction

Diabetic foot ulcers are among the most severe and resource-intensive complications of diabetes mellitus. Approximately 18.6 million people worldwide develop a diabetic foot ulcer each year, and these lesions precede most diabetes-related lower-extremity amputations and are associated with substantial functional impairment, recurrent infection, reduced quality of life, and increased mortality [1]. Although debridement, infection control, pressure offloading, restoration of adequate perfusion, and appropriate wound dressings remain the foundations of care, a considerable proportion of ulcers fail to achieve durable closure. This persistent clinical burden has driven the development of biological interventions intended to restore the cellular and molecular signals that are deficient or dysregulated in the diabetic wound environment.

Normal cutaneous repair requires the coordinated progression of hemostasis, inflammation, proliferation, re-epithelialization, extracellular matrix deposition, angiogenesis, and tissue remodeling. Diabetes disrupts this sequence through interacting metabolic, vascular, immune, and cellular abnormalities. Sustained hyperglycemia, advanced glycation end-products, oxidative stress, persistent inflammation, excessive protease activity, endothelial dysfunction, neuropathy, and impaired perfusion collectively delay the transition from inflammation to tissue reconstruction [2]. These disturbances compromise macrophage functional transitions, keratinocyte migration, fibroblast proliferation, collagen organization, endothelial cell activity, pericyte recruitment, and vascular maturation. Consequently, diabetic wounds are not simply acute wounds that heal more slowly; they represent a biologically hostile and dynamically dysregulated microenvironment in which endogenous reparative signals may be insufficient, rapidly degraded, or functionally uncoupled from their target cells [2,3].

Platelet-derived growth factor-BB (PDGF-BB) is a particularly relevant mediator of tissue repair. It is released early after injury by activated platelets and is subsequently produced by macrophages, fibroblasts, endothelial cells, and other cells within the wound. Through activation of PDGFR-α and PDGFR-β, PDGF-BB engages signaling pathways that include RAS–RAF–MEK–ERK, PI3K–AKT, and PLC-γ. These pathways support chemotaxis, proliferation, survival, extracellular matrix production, fibroblast recruitment, granulation tissue formation, and pericyte-mediated vascular stabilization [3]. This biological rationale led to the development of recombinant human PDGF-BB (rhPDGF-BB), including becaplermin, as a topical therapeutic strategy for diabetic wounds. A pivotal randomized, double-blind, placebo-controlled trial reported that becaplermin administered with standardized wound care increased complete closure and reduced the time to healing in chronic neuropathic diabetic ulcers [4].

However, the therapeutic position of growth factor treatment remains unsettled. Despite the biological plausibility of PDGF-BB and favorable findings in early clinical development, the current International Working Group on the Diabetic Foot guideline conditionally suggests against the routine use of growth factor therapy as an adjunct to standard care because the supporting clinical evidence is limited and of low certainty [5]. This apparent disconnect between mechanistic plausibility, selected favorable trials, and cautious clinical recommendations highlights the need to examine the preclinical evidence more critically. The effectiveness of an exogenously administered protein depends not only on receptor activity but also on its stability, local concentration, tissue retention, and duration of exposure. Chronic wound fluids can rapidly degrade PDGF-BB and other peptide growth factors through excessive proteolytic activity [6]. Hydrogels, nanofibrous scaffolds, extracellular-matrix-binding constructs, and controlled-release systems have, therefore, been developed to protect growth factors, restrict unwanted diffusion, and prolong local bioavailability [7]. Nevertheless, multicomponent platforms may also possess independent biological activity, making it difficult to determine whether an observed benefit results from rhPDGF-BB, the delivery vehicle, another active component, or their interaction.

The experimental evidence for rhPDGF-BB in diabetic wound healing is distributed across diverse animal models, formulations, dosing schedules, comparators, assessment times, and outcome domains. Some studies evaluated rhPDGF-BB or becaplermin directly, whereas others incorporated the growth factor into engineered proteins, biomaterials, or combination therapies. This heterogeneity has hindered quantitative synthesis and has encouraged broad interpretation of formulation-level effects as if they represented the isolated action of rhPDGF-BB. Moreover, the extent to which favorable macroscopic findings are accompanied by consistent histological, extracellular matrix, vascular, inflammatory, or molecular responses remains uncertain.

Accordingly, this systematic review and meta-analysis aimed primarily to determine the preclinical efficacy attributable specifically to exogenously administered rhPDGF-BB in experimental diabetic cutaneous wound healing. Studies evaluating rhPDGF-BB-containing formulations were included within the same review to characterize the influence of different delivery contexts; however, when the experimental comparator did not permit isolation of the contribution of rhPDGF-BB, the findings were interpreted only at the formulation level and not as evidence of the independent efficacy of the growth factor. The objectives were to characterize the animal and wound models, treatment protocols, delivery systems, and comparators; quantify effects on wound closure and time to complete closure when sufficiently compatible data were available; synthesize non-meta-analyzable findings across prespecified reparative domains; assess risk of bias and certainty of evidence; and distinguish directly attributable rhPDGF-BB effects from formulation-level signals. By integrating quantitative, descriptive, mechanistic, and attribution-specific evidence, this review sought to define more precisely the preclinical efficacy of rhPDGF-BB and the conditions required for its credible translation.

## 2. Results

### 2.1. Study Selection

The electronic database searches identified 3844 records: 398 from PubMed, 1415 from Embase, 1298 from Scopus, 592 from Web of Science Core Collection, and 141 from ScienceDirect (54 from Search A and 87 from Search B). After the removal of 2049 duplicate records, 1795 unique records remained for title and abstract screening. Of these, 1725 were excluded because they did not meet the predefined eligibility criteria, and 70 reports were sought for full-text retrieval.

Five reports could not be retrieved; therefore, 65 reports underwent full-text eligibility assessment. Thirty-six reports were excluded: 22 because the intervention was ineligible or could not be verified as recombinant human PDGF-BB, or because rhPDGF-BB/becaplermin was not administered to animals with diabetes; six because they were conference abstracts with insufficient methodological and outcome information; and eight because they were secondary or duplicate reports associated with complete primary studies.

**Figure 1:**
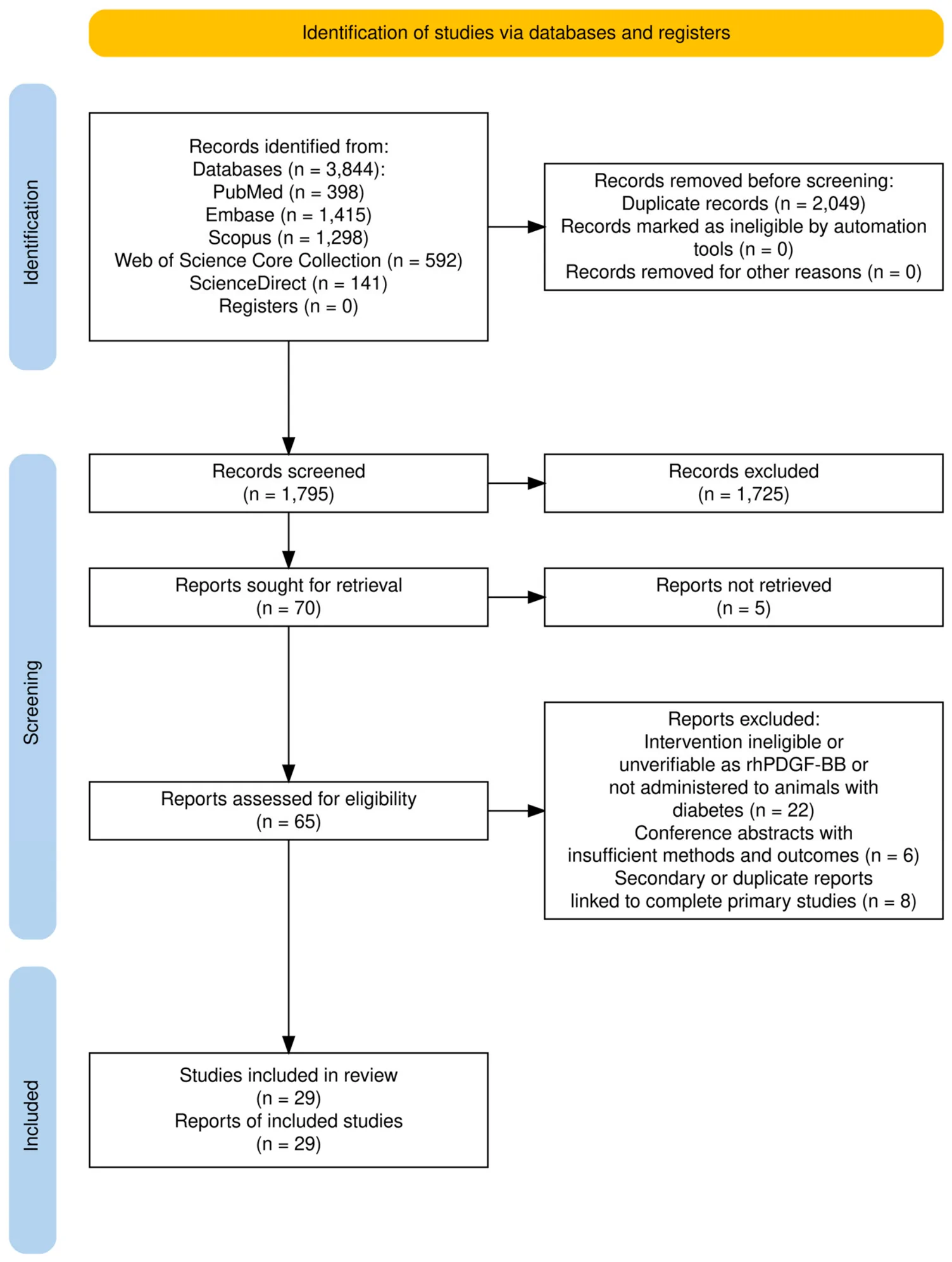
PRISMA 2020 flow diagram showing the identification, screening, eligibility assessment, and inclusion of studies in this systematic review.

Ultimately, 29 reports representing 29 unique controlled in vivo studies fulfilled the eligibility criteria and were included in this systematic review. No reports remained awaiting classification. The complete study selection process is presented in the PRISMA 2020 flow diagram (Figure 1).

### 2.2. Characteristics of the Included Studies

Twenty-nine controlled in vivo studies published between 1990 and 2025 were included [8,9,10,11,12,13,14,15,16,17,18,19,20,21,22,23,24,25,26,27,28,29,30,31,32,33,34,35,36]. Sixteen studies were conducted in the United States [12,13,14,17,21,23,25,26,27,28,29,32,33,34,35,36], three each in Taiwan [8,10,11] and India [15,20,30], two each in China [9,19] and Croatia [18,22], and one each in Norway [16] and Germany [24]. One study was conducted collaboratively in Australia and Japan [31].

Most studies used mouse models (22/29) [8,9,13,14,16,17,18,20,21,23,24,25,26,27,28,29,30,31,32,33,34,35], whereas six used rats [10,11,15,19,22,36], and one used Yorkshire pigs [12]. Genetic models involving leptin-receptor dysfunction, predominantly db/db mice, were the most frequently employed models of diabetes (16/29) [13,14,16,17,18,21,23,25,26,27,28,29,31,33,34,35]. Diabetes or hyperglycemia was induced using streptozotocin in eight studies [8,9,10,11,12,24,30,36] and alloxan in three [15,20,22]. White et al. used non-obese diabetic mice with spontaneous type 1 diabetes [32], whereas Cheng et al. used rats with pre-existing experimental diabetes for which the induction method was not reported [19].

Fifteen studies exclusively used male animals [8,9,10,13,15,19,20,22,23,25,30,31,34,35,36], five exclusively used females [12,18,28,32,33], and two included animals of both sexes [24,29]. Sex was not reported in seven studies [11,14,16,17,21,26,27]. Random allocation was explicitly reported in twelve studies [9,10,11,13,24,28,29,30,32,33,35,36]. Most studies employed parallel-group experimental designs; however, some used paired wounds assigned to different treatments within the same animal [12,21,36], repeated independent experiments, or separate terminal cohorts assessed at predefined time points [18,22,26,28,29,34,36].

Reported sample sizes varied considerably, ranging from three animals per group [8,17] to 104 animals across two experiments [27]. In several studies, the total number of unique animals could not be determined because sample sizes were reported according to wounds, experimental replicates, treatment groups, assessment time points, or terminal cohorts [12,22,23,26,28,29,31,32,34,35,36]. Follow-up periods also varied substantially, ranging from assessments performed 3 days after wounding [32] to observation until complete healing for up to 63 days [27]. One early study additionally followed a persistent chronic wound until day 90 [29]. The general characteristics of the included studies and animal models are summarized in Table 1.

All studies employed full-thickness cutaneous wound models [8,9,10,11,12,13,14,15,16,17,18,19,20,21,22,23,24,25,26,27,28,29,30,31,32,33,34,35,36]. Twenty-eight studies created wounds on the dorsal or dorsolateral trunk [8,9,10,11,12,13,14,15,16,17,18,19,20,21,22,23,25,26,27,28,29,30,31,32,33,34,35,36], whereas Uhl et al. used a full-thickness ear wound model [24]. Circular excisional wounds were used in 22 studies [8,9,10,11,13,14,15,18,19,20,21,22,23,24,25,30,31,32,33,34,35,36], while seven employed square or rectangular wounds [12,16,17,26,27,28,29]. Sixteen studies created a single wound per animal [8,9,15,16,17,18,20,22,23,24,25,26,27,28,29,33], eight created two wounds [10,11,13,14,21,31,32,34], and four created four wounds [19,30,35,36]. O’Brien et al. used multiple wounds arranged in paired dorsal fields, with triplicate wounds assigned to each treatment within the same animal [12]. Mechanical splinting to limit wound contraction was explicitly reported in only four mouse studies [13,14,32,33]; most remaining models relied on dressings or left wounds uncovered without rigid splinting.

Commercial becaplermin or Regranex formulations were evaluated in 13 studies [12,14,15,17,19,20,21,22,23,25,30,33,36]. In ten of these, becaplermin served as an active reference comparator against another experimental intervention [12,15,17,20,21,22,25,30,33,36], whereas it constituted a principal treatment arm in the remaining three [14,19,23]. Other studies evaluated purified, recombinant, or engineered human PDGF-BB as the primary intervention or as a component of a combination treatment [8,9,10,11,13,16,18,24,26,27,28,29,31,32,34,35]. Structured delivery systems included a PEI–polypyrrole–Pluronic F127 thermosensitive hydrogel [8], silk-sericin hydrogels [9], fibrin matrices [32,35], a PLGA–collagen scaffold [10], and a PLGA nanofibrous membrane [11]. Engineered PDGF-BB variants were additionally examined by Alshoubaki et al. [31] and White et al. [32].

Attribution was determined at the intervention–comparator contrast level; therefore, a single publication could contribute to more than one attribution category. Direct attribution of the observed effects to rhPDGF-BB or becaplermin was possible in 26 studies. Seventeen studies contained only directly attributable comparisons (D) [10,11,12,13,15,17,18,19,20,21,22,23,24,25,30,33,36], whereas nine contained both directly attributable and formulation-level comparisons (D/F) [9,14,26,27,28,29,31,32,34]. Three studies evaluated rhPDGF-BB only as part of a multicomponent formulation without an equivalent formulation-minus-factor comparator and, therefore, contributed formulation-level evidence (F) exclusively [8,16,35]. Accordingly, 26 studies provided at least one directly attributable comparison, and 12 provided at least one formulation-level comparison.

Administration was predominantly topical, either directly onto the wound bed or beneath a dressing. Gallant-Behm et al. administered rhPDGF-BB through periwound intradermal injections [34], while Lee et al. used the single placement of a PDGF-BB-containing scaffold or membrane [10,11]. Nine studies described a single administration or device placement [9,10,11,12,17,24,31,32,35], whereas the remaining studies used repeated treatment schedules [8,13,14,15,16,18,19,20,21,22,23,25,26,27,28,29,30,33,34,36]. Fixed repeated regimens ranged from four consecutive daily applications [20] to administration every other day through day 24, corresponding to 13 applications [27]. One study continued daily treatment until wound closure [14].

Substantial heterogeneity was observed in the reporting of rhPDGF-BB exposure. Among studies reporting an absolute active amount per wound, doses ranged from 50 ng per application as part of a dual-growth-factor fibrin formulation [35] to 20 µg per wound per day [22]. Other studies expressed exposure relative to wound area, including 7 µg/cm^2^ [19] and 4 or 40 µg/cm^2^ [27]. Several reports provided only the concentration of the formulation, the mass or volume of gel applied, or the amount used during scaffold fabrication; therefore, the exact active dose delivered per wound could not be reconstructed [9,10,11,12,15,17,20,21,25,30,36]. Comparator structures included untreated diabetic wounds, vehicle or carrier controls, unloaded delivery systems, nondiabetic reference groups, and active experimental treatments [8,9,10,11,12,13,14,15,16,17,18,19,20,21,22,23,24,25,26,27,28,29,30,31,32,33,34,35,36]. Combination regimens incorporated other growth factors—including EGF, VEGF, IGF, TGF-α, and FGF—or non-growth-factor modulators such as miRNA inhibitors and AMD3100 [9,14,16,26,27,28,29,32,34,35]. These differences in comparator composition were considered when determining whether the effect could be attributed directly to rhPDGF-BB or only to the complete formulation.

Across the 29 studies, 160 tabulated experimental entries were compiled and are detailed in Table S1 [8,9,10,11,12,13,14,15,16,17,18,19,20,21,22,23,24,25,26,27,28,29,30,31,32,33,34,35,36]. The number of entries varied from two in the simplest two-arm experiments [13,24] to 18 across the multiple dose- and duration-based experiments reported by Greenhalgh et al. [29]. Reported sample sizes were generally expressed as animals, but four studies reported the relevant experimental unit as wounds [12,31,35,36]. Other studies reported sample sizes by dose, experiment, replicate, assessment time point, or terminal cohort rather than as a single number of unique animals [17,18,22,26,28,29,34]. Moreover, some treatments were allocated to different wounds within the same animal [12,21,36]. Accordingly, the original unit and level of reporting were retained without converting wounds, repeated experiments, or terminal cohorts into inferred totals of independent animals. The wound models and treatment protocols are summarized in Table 2, and the detailed experimental groups, reported sample sizes, and eligible comparisons are provided in Table S1.

Twenty-four studies assessed wound dimensions macroscopically or through intravital imaging [8,9,10,11,12,13,14,15,16,17,18,19,20,22,23,24,25,26,27,28,29,30,33,36]. Most relied on serial wound photography or wound-edge tracing followed by manual or computerized planimetry [8,9,10,11,12,13,14,15,16,17,19,20,22,23,25,26,27,28,29,30,36], whereas Uhl et al. used intravital fluorescence microscopy to measure wound surface area and re-epithelialization [24]. Tkalčević et al. reported gross closure status in terminal cohorts, although the quantitative macroscopic method was not described [18]. In the remaining five studies, wound closure, wound extent, or healing stage was determined histomorphometrically or microscopically without serial macroscopic imaging [21,31,32,34,35]. The most frequently reported measures were residual or open wound area and percentage closure or contraction [8,9,10,11,12,13,14,15,16,17,19,20,22,23,24,25,26,27,28,29,30,33,36]. Time to partial or complete closure was additionally evaluated in selected studies [14,23,24,25,27].

Histological repair was assessed in 26 studies, predominantly using hematoxylin and eosin staining or conventional histomorphology to examine re-epithelialization, epithelial gap or wound width, granulation tissue formation, inflammatory cell infiltration, and overall tissue architecture [8,9,10,11,12,13,15,16,17,18,19,20,21,22,23,25,26,28,29,30,31,32,33,34,35,36]. Histological endpoints were assigned to the prespecified outcome domains according to the construct assessed, whereas the measurement approach was recorded separately as qualitative, semiquantitative, or quantitative. Qualitative assessments comprised descriptive morphological observations without numerical scoring; semiquantitative assessments comprised ordinal or composite histological scores; and quantitative assessments comprised continuous histomorphometric or image analysis measurements. Collagen deposition and extracellular matrix remodeling were evaluated in 20 studies [8,9,10,11,12,13,15,16,17,18,19,20,21,22,25,26,28,29,30].

These assessments included histochemical staining with Masson’s trichrome, Picrosirius red, Van Gieson, or Mallory methods [8,9,12,13,16,18,20,21,22,28,29,30]; biochemical quantification of hydroxyproline and hexosamine [15]; molecular or enzymatic evaluation of collagen I, MMP-8, MMP-9, and α-SMA [10,11,17,33]; and qualitative or semiquantitative histological assessment of collagen organization or fibrosis [19,25,26]. Angiogenesis or tissue perfusion was examined in 23 studies [8,9,11,12,14,15,16,17,18,19,20,21,22,24,25,26,28,29,31,32,34,35,36]. CD31 or PECAM-1 immunostaining, immunofluorescence, or flow cytometry was the most commonly used approach [8,9,12,14,17,20,31,32,34,35]. Other methods included VEGF immunohistochemistry [36], intravital fluorescence microscopy and laser Doppler perfusion measurements [24], and qualitative or semiquantitative evaluation of vascularity within histological sections or composite healing scores [11,15,16,18,19,21,22,25,26,28,29].

Inflammation or immune cell responses were assessed in 24 studies [8,9,10,11,12,13,15,16,19,20,21,22,23,24,25,26,28,29,30,31,32,33,35,36]. Although many studies relied on histological characterization of inflammatory cell infiltration, more specific approaches included CD206 immunofluorescence, macrophage-associated gene or protein expression, flow-cytometric characterization of leukocyte populations, ROS and NF-κB measurements, mast cell staining, calprotectin or F4/80 immunohistochemistry, and intravital analysis of leukocyte rolling and adhesion [9,12,16,24,30,32,33,35,36].

Proliferation or molecular-signaling outcomes were evaluated in ten studies [14,15,18,19,26,30,32,34,35,36]. These analyses included Nrf2- and Akt-related signaling [30], flow-cytometric assessment of Ki-67-positive proliferating cells in wound tissue [32], ITGA5 expression [34], endothelial Ki-67 labeling [35], BrdU incorporation and PDGF or FGF-2 immunostaining [36], circulating progenitor cell measurements [14], biochemical and cellular proliferation markers [15], desmin and α-SMA immunostaining [18], PCNA labeling and c-Fos, ERK/phospho-ERK, and α-SMA assessment [19], and apoptosis assessment [26].

Reporting of adverse effects and treatment tolerability was inconsistent. Thirteen of the 29 included studies documented at least one local or systemic safety-related variable, including local tissue reactions, wound infection, general clinical condition, body weight, food intake, blood glucose, biochemical indicators of hepatic or renal function, treatment tolerance, or mortality [8,10,11,13,16,19,20,24,27,29,32,34,36]. Only Gallant-Behm et al. [34] reported systemic biochemical testing, comprising plasma alanine aminotransferase, aspartate aminotransferase, blood urea nitrogen, and creatinine measurements. In Saklani et al.’s study [20], the OECD 402 dermal toxicity observations were performed in the peptide hydrogel groups and were not specific to becaplermin. White et al. [32] reported daily wound monitoring and observed no signs of wound infection, although other treatment-related adverse effects were not reported. The remaining 16 studies either did not perform a relevant in vivo wound model safety assessment or provided insufficient information to determine treatment-related adverse effects [9,12,14,15,17,18,21,22,23,25,26,28,30,31,33,35]. Safety-specific follow-up was generally not distinguished from efficacy follow-up, attrition and exclusions were inconsistently reported, and several observations could be attributed only to the complete intervention or formulation rather than specifically to rhPDGF-BB. Study-level safety variables, follow-up durations, reported adverse events, attrition, systemic assessments, and the permitted level of attribution are presented in Table S2. For studies without an eligible safety assessment, the absence of reported adverse events was classified as not assessable rather than as evidence that adverse effects were absent. Assessment schedules varied substantially. Early inflammatory, molecular, histological, or microcirculatory measurements were performed from day 1 after wounding [24,33,36], whereas longitudinal macroscopic follow-up extended until complete healing for up to 63 days [27]. Greenhalgh et al. monitored wounds for up to six weeks and followed one persistent chronic wound to day 90 [29]. Macroscopic outcomes were generally measured longitudinally, while histological, immunohistochemical, biochemical, and molecular analyses were commonly performed at predefined terminal time points. In Cheng et al.’s study, the direct becaplermin comparison provided macroscopic-healing data, whereas the histological and immunohistochemical analyses corresponded to a separate peptide–placebo experiment [17]. The early histological assessment time in Chan et al.’s study [23] was classified as unclear because it was reported as day 7 in the Methods but labeled as day 5 in the corresponding figure; the day-24 assessment was reported consistently. The outcome domains, original assessment methods, safety variables, and evaluation time points for each included study are summarized in Table 3.

### 2.3. Risk-of-Bias Assessment

**Figure 2:**
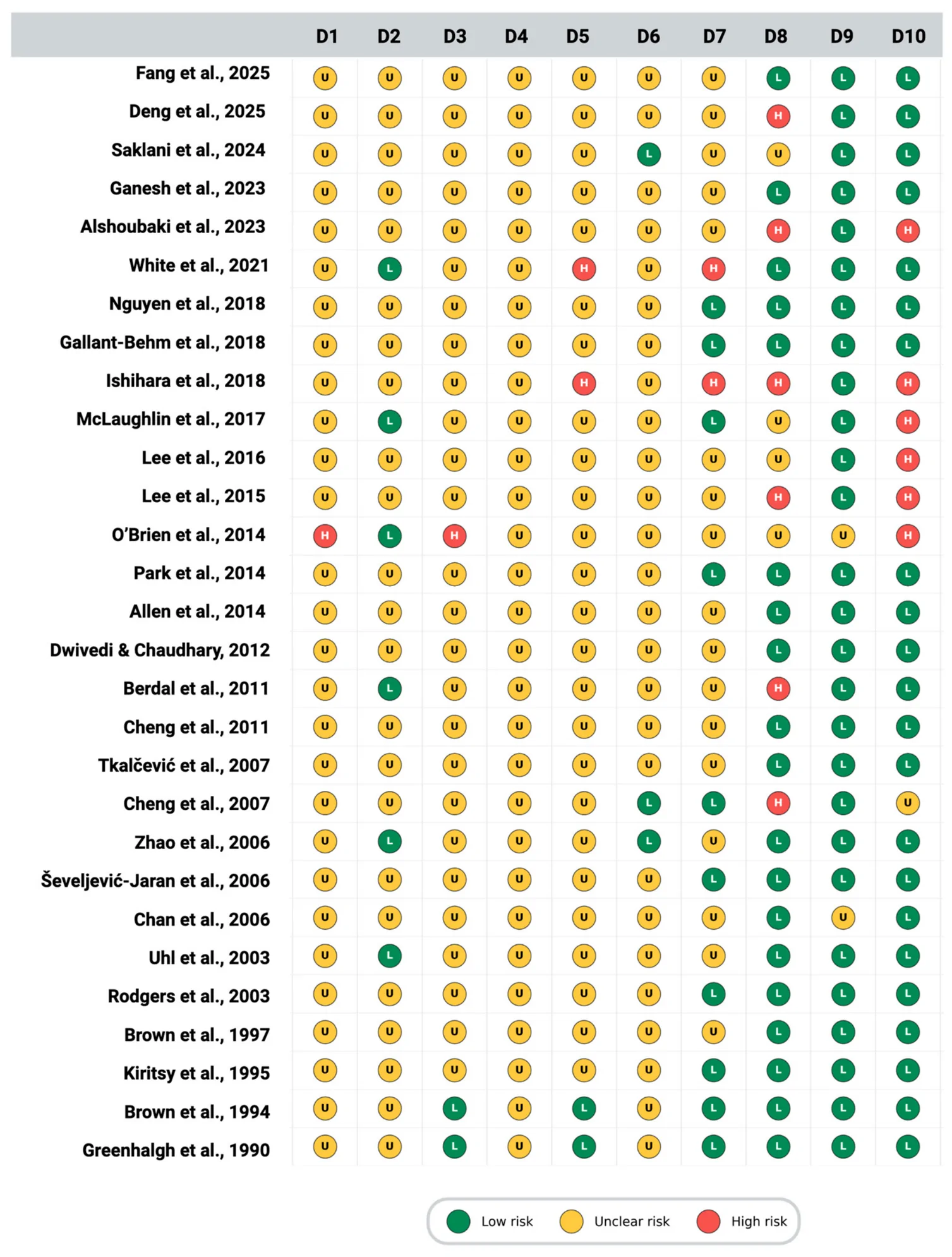
Risk-of-bias judgments for the 29 included studies across the 10 domains of the SYRCLE tool [8,9,10,11,12,13,14,15,16,17,18,19,20,21,22,23,24,25,26,27,28,29,30,31,32,33,34,35,36]. D1, sequence generation; D2, baseline characteristics; D3, allocation concealment; D4, random housing; D5, blinding of caregivers and investigators; D6, random outcome assessment; D7, blinding of outcome assessors; D8, incomplete outcome data; D9, selective outcome reporting; D10, other sources of bias. L, low risk; U, unclear risk; H, high risk. No overall study-level judgment was assigned. Created in BioRender. Carrillo, J. (2026) https://BioRender.com/oc9avnt.

**Figure 3:**
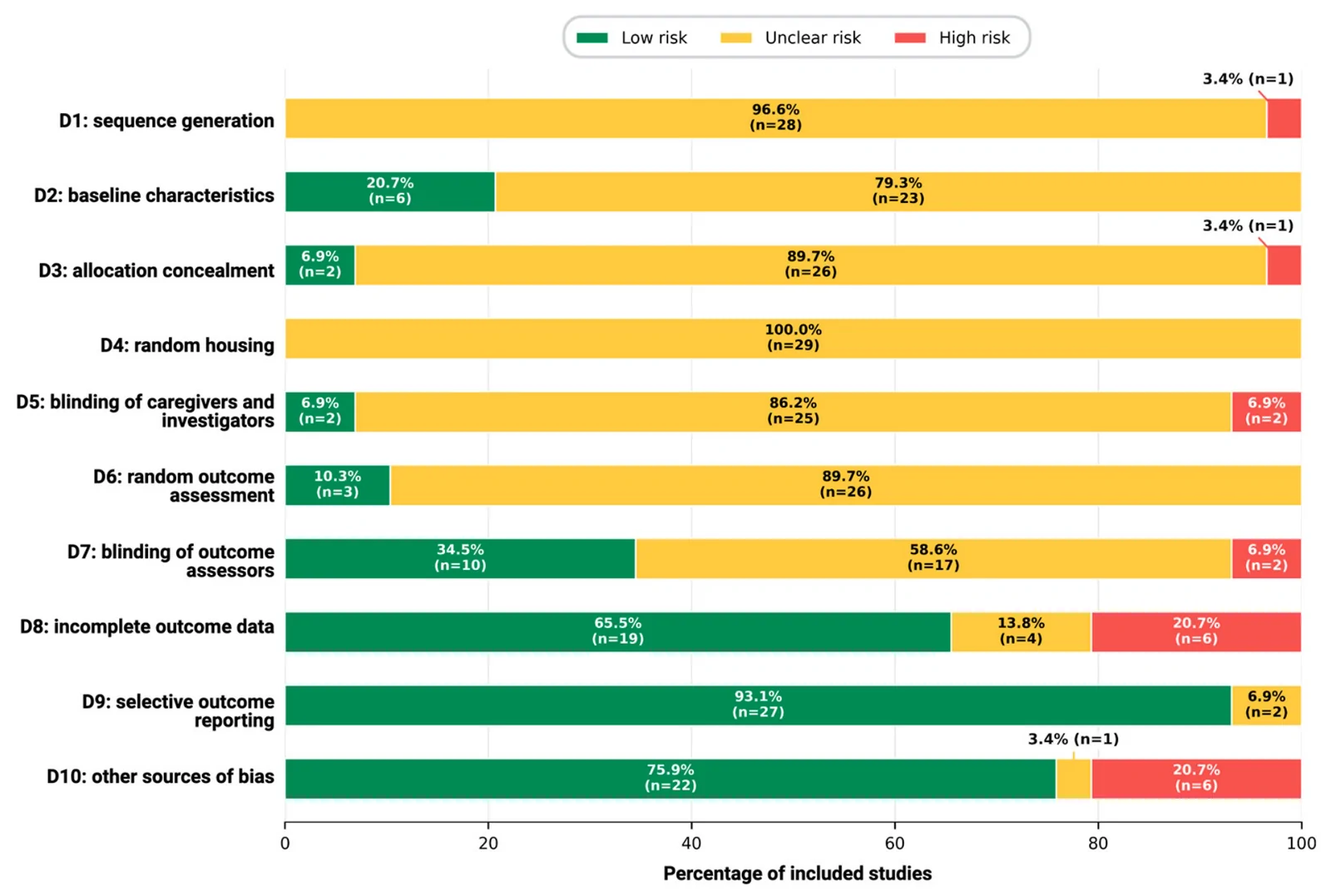
Distribution of low-, unclear-, and high-risk judgments across the 10 SYRCLE domains. Each domain comprises 29 study-level judgments [8,9,10,11,12,13,14,15,16,17,18,19,20,21,22,23,24,25,26,27,28,29,30,31,32,33,34,35,36]. Labels show the percentage (%) and number of studies (n) in each category; percentages may not total 100% because of rounding. No overall study-level judgment was calculated. Created in BioRender. Carrillo, J. (2026) https://BioRender.com/an85zvq.

Risk of bias was assessed for all 29 included animal studies [8,9,10,11,12,13,14,15,16,17,18,19,20,21,22,23,24,25,26,27,28,29,30,31,32,33,34,35,36] across the 10 SYRCLE domains (Figure 2), and the domain-level distribution is summarized in Figure 3. No composite numerical quality score or overall study-level risk-of-bias judgment was calculated. Of the 290 domain-level judgments, 91 (31.4%) were rated as low risk, 181 (62.4%) as unclear risk, and 18 (6.2%) as high risk. Sequence generation was rated as unclear in 28 studies [8,9,10,11,13,14,15,16,17,18,19,20,21,22,23,24,25,26,27,28,29,30,31,32,33,34,35,36] because none reported a reproducible method for generating the allocation sequence, including studies that merely stated that animals or wounds were randomly assigned. O’Brien et al. [12] was rated as high risk because treatment and control wounds were allocated using a fixed, non-random positional scheme. No study was rated as having a low risk of bias for sequence generation. Baseline characteristics were rated as low risk in six studies [12,16,21,24,32,36] that demonstrated group comparability, evaluated relevant baseline covariates, or used matched within-animal comparisons. The remaining 23 studies were rated as unclear risk [8,9,10,11,13,14,15,17,18,19,20,22,23,25,26,27,28,29,30,31,33,34,35] because baseline equivalence between the eligible groups was not adequately demonstrated. No study was rated as high risk in this domain. Allocation concealment was rated as low risk in two coded and blinded experiments [28,29], high risk in the study that used a fixed and predictable allocation scheme [12], and unclear in the remaining 26 studies [8,9,10,11,13,14,15,16,17,18,19,20,21,22,23,24,25,26,27,30,31,32,33,34,35,36] because the method used to conceal treatment assignment was not reported.

Random housing was rated as unclear in all 29 studies [8,9,10,11,12,13,14,15,16,17,18,19,20,21,22,23,24,25,26,27,28,29,30,31,32,33,34,35,36], as none reported randomization of cage placement, housing location, or the position of animals within the animal facility. No study was rated as either low or high risk in this domain. Blinding of caregivers and investigators during the intervention was rated as low risk in two explicitly coded and blinded experiments [28,29]. Two studies were rated as high risk because they explicitly reported that blinding was not possible or that the animal experiments were not blinded [32,35]. The remaining 25 studies were rated as unclear risk [8,9,10,11,12,13,14,15,16,17,18,19,20,21,22,23,24,25,26,27,30,31,33,34,36] because intervention-period blinding was not adequately described. Random outcome assessment was rated as low risk in three studies that randomly selected animals or microscopic fields for assessment [19,20,21]. The other 26 studies were rated as unclear risk [8,9,10,11,12,13,14,15,16,17,18,22,23,24,25,26,27,28,29,30,31,32,33,34,35,36] because random selection or ordering of animals, samples, sections, fields, or measurements during outcome assessment was not reported. No study was rated as high risk in this domain.

Blinding of outcome assessors was rated as low risk in 10 studies that reported blinded, masked, or coded assessment of the principal eligible outcomes [13,19,22,25,27,28,29,33,34,36]. Two explicitly non-blinded studies were rated as high risk [32,35]. The remaining 17 studies were rated as unclear risk [8,9,10,11,12,14,15,16,17,18,20,21,23,24,26,30,31] because assessor blinding was not reported or was described only for outcomes that did not permit a low-risk judgment for the principal eligible assessments.

Incomplete outcome data were rated as low risk in 19 studies in which the reported sample sizes were retained, terminal sampling was planned and adequately accounted for, or losses and exclusions were sufficiently explained [8,13,14,15,17,18,21,22,23,24,25,26,27,28,29,30,32,33,34]. Four studies were rated as unclear risk because the analyzed denominators or terminal cohorts could not be fully reconstructed from the available information [10,12,20,36]. Six studies were rated as high risk because of unexplained reductions in outcome-specific sample sizes, outcome-related exclusions, or missing observations that were not adequately addressed [9,11,16,19,31,35]. Selective outcome reporting was rated as low risk in 27 studies in which the outcomes described for the eligible animal experiments were reported without a clear omission [8,9,10,11,13,14,15,16,17,18,19,20,21,22,24,25,26,27,28,29,30,31,32,33,34,35,36]. Two studies were rated as unclear risk because internal or experiment-specific inconsistencies prevented confirmation that all expected outcomes and assessment times had been reported as planned [12,23]. No study was rated as high risk in this domain.

Other sources of bias were rated as low risk in 22 studies in which no additional methodological problem was identified [8,9,13,14,15,16,17,18,20,21,22,23,24,25,26,27,28,29,30,32,33,34]. One study was rated as unclear risk because the unit of analysis could not be definitively established [19]. Six studies were rated as high risk because of confirmed or substantively demonstrated unit-of-analysis problems involving multiple wounds within the same animal or otherwise non-independent observations that were not appropriately addressed in the design or analysis [10,11,12,31,35,36]. These judgments were retained at the individual-domain level and were not combined into a global study score.

### 2.4. Quantitative Synthesis

Experimental unit assessment confirmed that no pooled estimate treated individual wounds or repeated observations as independent experimental units. In the percentage wound closure analysis, Deng et al. [9] and Greenhalgh et al. [29] used one wound per mouse. Park et al. [13] created two bilateral wounds per mouse; however, both wounds received the same treatment, and the original investigators averaged the bilateral measurements to obtain one value per mouse. Accordingly, the reported sample size of 12 per group was retained at the animal level. The four eligible component experiments reported by Greenhalgh et al. [29] were combined within the publication: intervention groups sharing a common control were first combined, and the remaining independent experiment-level estimates were then pooled to obtain a single study-level estimate. In the time-to-complete-closure analysis, Uhl et al. [24] and Kiritsy et al. [27] each used one wound per mouse, and the extracted group sizes represented animals. Serial measurements were not entered as separate estimates; one estimate from the prespecified follow-up window or one time-to-closure summary per study was used. Consequently, neither quantitative synthesis contained an unresolved unit-of-analysis problem, and no within-animal correlation imputation or unit-of-analysis exclusion sensitivity analysis was required.

#### 2.4.1. Percentage Wound Closure at Days 10–12

**Figure 4:**
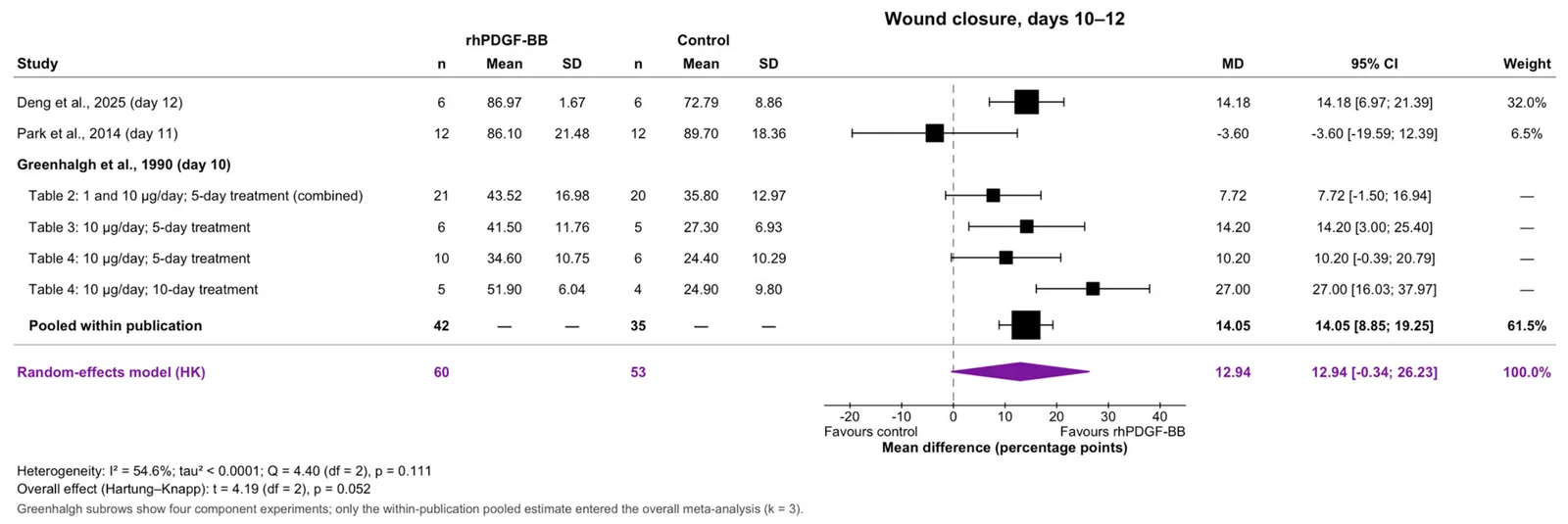
Meta-analysis of percentage wound closure at days 10–12 using directly attributable rhPDGF-BB comparisons. Deng et al. [9] compared a PDGF-BB-functionalized sericin hydrogel with the matched wild-type sericin hydrogel lacking the growth factor, whereas Park et al. [13] and Greenhalgh et al. [29] compared non-combination rhPDGF-BB treatment with factor-free vehicle controls. The four eligible component experiments reported by Greenhalgh et al. [29] were pooled within the publication before inclusion in the overall analysis, thereby preventing multiple counting of a single study. Mean differences (MDs) were synthesized using a random-effects model with restricted maximum-likelihood estimation and Hartung–Knapp adjustment. Positive MDs favor the rhPDGF-BB-containing intervention over its corresponding factor-free comparator. Squares represent study estimates, with the area of each square proportional to the study’s relative inverse-variance weight in the random-effects model; horizontal lines indicate 95% confidence intervals (CIs), and the diamond represents the pooled effect. n, number of animals in the corresponding group; SD, standard deviation.

Three studies—Deng et al.’s [9] at day 12, Park et al.’s [13] at day 11, and Greenhalgh et al.’s [29] at day 10—comprising 113 animals (60 receiving an rhPDGF-BB-containing intervention and 53 factor-free controls) were included in the primary meta-analysis of percentage wound closure at days 10–12 (Figure 4). All contributing contrasts met the criteria for direct attribution. Deng et al. [9] compared a PDGF-BB-functionalized sericin hydrogel with the matched wild-type sericin hydrogel lacking the growth factor, whereas Park et al. [13] and Greenhalgh et al. [29] compared non-combination rhPDGF-BB treatment with factor-free vehicle controls. Because each exact assessment day was represented by only one study providing the numerical information required to estimate an effect and its variance, a same-day sensitivity meta-analysis could not be performed. Before inclusion in the overall model, the four eligible component experiments reported by Greenhalgh et al. [29] were combined to obtain a single study-level estimate. The random-effects pooled estimate for these directly attributable comparisons numerically favored the rhPDGF-BB-containing intervention, corresponding to a 12.94-percentage-point increase in wound closure; however, the difference did not reach statistical significance (MD 12.94 percentage points; 95% CI: −0.34 to 26.23; *p* = 0.052). Heterogeneity estimates were imprecise because only three studies were available (I^2^ = 54.6%; τ^2^ < 0.0001; Q = 4.40; *p* = 0.111). Although these assessments were separated by only two calendar days, they should not be assumed to represent an identical biological stage. Healing kinetics may differ between the STZ-induced model used by Deng et al. [9] and the db/db models used by Park et al. [13] and Greenhalgh et al. [29], as well as between the splinted wounds evaluated by Park et al. [13] and the non-splinted wounds evaluated in the other two studies. The pooled estimate should, therefore, be interpreted as representing a narrow calendar time window that may still contain temporal and model-related heterogeneity.

**Figure 5:**
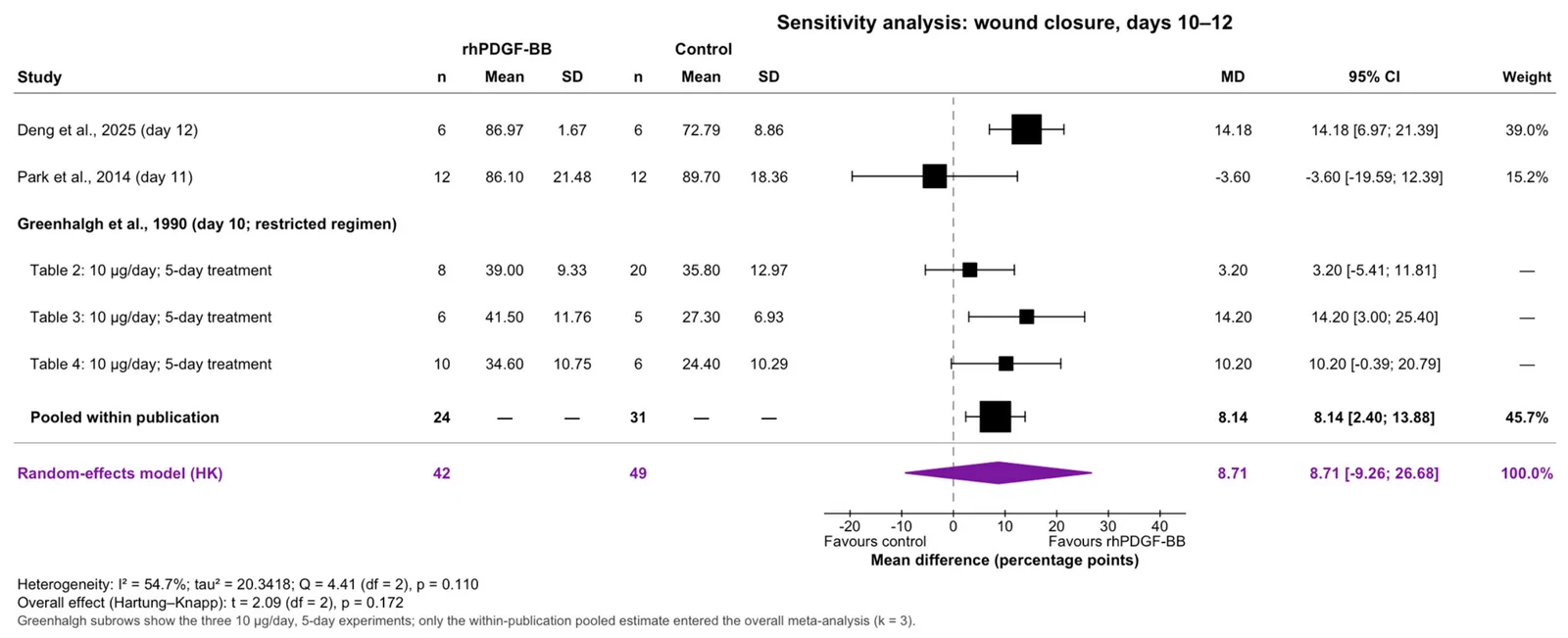
Sensitivity analysis of percentage wound closure at days 10–12 using directly attributable rhPDGF-BB comparisons. The contribution of Greenhalgh et al. [29] was restricted to component experiments evaluating non-combination rhPDGF-BB at 10 µg/day for 5 days against the corresponding factor-free vehicle controls; these experiments were pooled within the publication before inclusion in the overall analysis. The directly attributable comparisons from Deng et al. [9] and Park et al. [13] were retained unchanged. Mean differences (MDs) were synthesized using a random-effects model with restricted maximum-likelihood estimation and Hartung–Knapp adjustment. Positive MDs favor the rhPDGF-BB-containing intervention over its corresponding factor-free comparator. Squares represent study estimates, with the area of each square proportional to the study’s relative inverse-variance weight in the random-effects model; horizontal lines indicate 95% confidence intervals (CIs), and the diamond represents the pooled effect. n, number of animals in the corresponding group; SD, standard deviation.

A sensitivity analysis restricted the contribution of Greenhalgh et al. [29] to experiments evaluating rhPDGF-BB at 10 µg/day for 5 days (Figure 5). This analysis retained the same direct-attribution structure as the primary model and comprised 91 animals (42 receiving an rhPDGF-BB-containing intervention and 49 factor-free controls). The pooled estimate for these directly attributable comparisons remained favorable to the rhPDGF-BB-containing intervention, although its magnitude was smaller and its confidence interval wider than in the primary analysis. The difference remained statistically non-significant (MD: 8.71 percentage points; 95% CI: −9.26 to 26.68; *p* = 0.172). Heterogeneity estimates remained imprecise (I^2^ = 54.7%; τ^2^ = 20.34; Q = 4.41; *p* = 0.110). Thus, restricting the contribution of Greenhalgh et al. [29] did not reverse the direction of the pooled estimate, although substantial uncertainty remained.

Additional post hoc robustness analyses are summarized in Table S3. Leave-one-study-out estimates remained numerically favorable after sequential exclusion of the studies by Deng et al. [9] (MD: 6.91 percentage points; 95% CI: −103.18 to 117.00), Park et al. [13] (MD: 14.10 percentage points; 95% CI: −13.26 to 41.45), and Greenhalgh et al. [29] (MD: 6.78 percentage points; 95% CI: −104.58 to 118.14); however, all confidence intervals obtained with the safeguarded Hartung–Knapp procedure included the null. Greenhalgh et al. [29] contributed the largest model weight (61.5%). Influence diagnostics identified the studies by Deng et al. [9] and Greenhalgh et al. [29] as potentially influential according to at least one default screening criterion, principally Cook’s distance, whereas Park et al. [13] did not meet the default influence thresholds despite being directionally discordant. Given the availability of only three studies, these diagnostics were interpreted as evidence of overall model instability rather than as definitive identification of an outlying study. Excluding Deng et al. [9], the only contributing study judged at high risk of bias for incomplete outcome data, produced the same estimate as its leave-one-out analysis. Restriction to mechanically splinted wounds left only Park et al.’s study [13]; consequently, no pooled estimate was calculated, and its individual effect was MD: −3.60 percentage points (95% CI: −19.59 to 12.39). No formulation-level comparison and no study with unresolved experimental-unit concerns contributed to the primary model; therefore, these exclusions did not alter the analysis.

#### 2.4.2. Time to Complete Wound Closure

**Figure 6:**
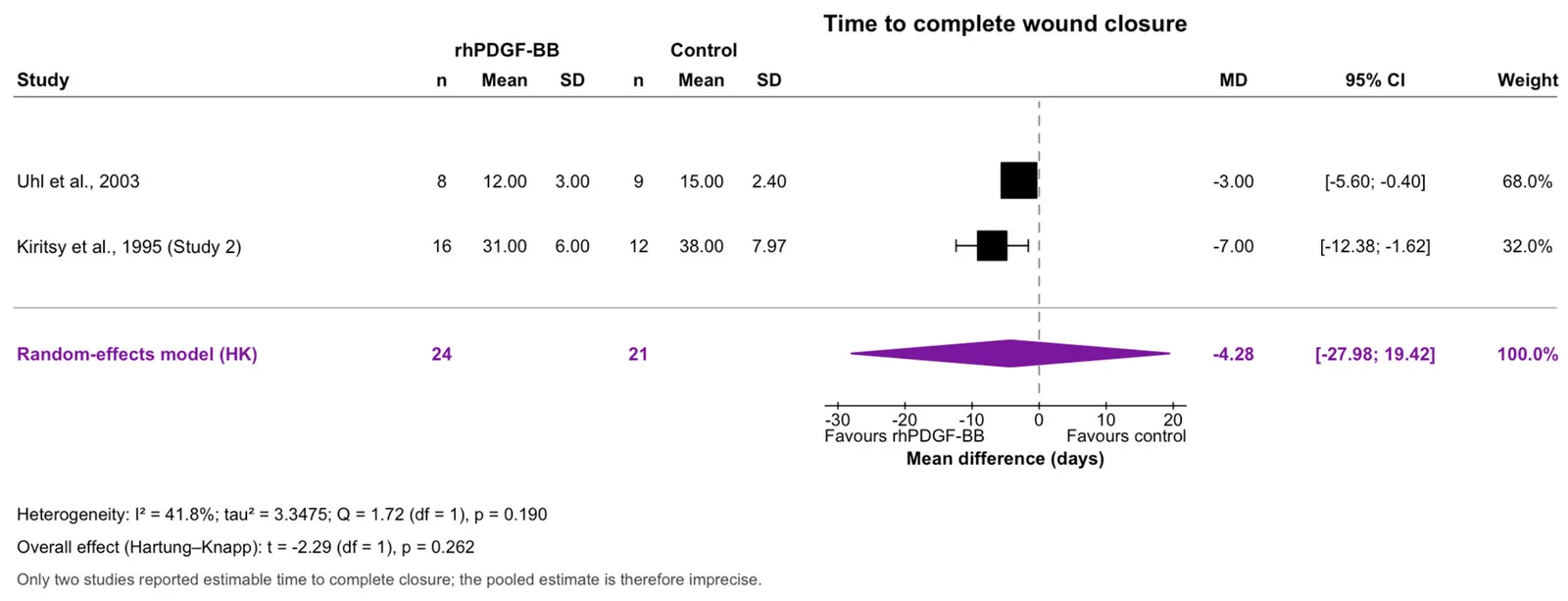
Meta-analysis of time to complete wound closure using directly attributable rhPDGF-BB comparisons. Uhl et al. [24] compared rhPDGF-BB with its carrier vehicle, whereas the eligible experiment from Kiritsy et al. [27] compared PDGF-BB monotherapy with a methylcellulose vehicle; the PDGF-BB–IGF-I combination evaluated by Kiritsy et al. was not included in this analysis. Mean differences (MDs) in time to complete wound closure were synthesized using a random-effects model with restricted maximum-likelihood estimation and Hartung–Knapp adjustment. Negative MDs indicate a shorter time to complete wound closure and, therefore, favor rhPDGF-BB over the corresponding factor-free comparator. Squares represent study estimates, with the area of each square proportional to the study’s relative inverse-variance weight in the random-effects model; horizontal lines indicate 95% confidence intervals (CIs), and the diamond represents the pooled effect. n, number of animals in the corresponding group; SD, standard deviation.

Two studies—Uhl et al. [24] and Kiritsy et al. [27]—comprising 45 animals (24 receiving rhPDGF-BB and 21 factor-free controls) provided compatible continuous data for time to complete wound closure (Figure 6). Both contributing contrasts met the criteria for direct attribution: Uhl et al. [24] compared a single application of rhPDGF-BB with its carrier vehicle, whereas the eligible experiment from Kiritsy et al. compared PDGF-BB monotherapy with a methylcellulose vehicle. Both individual estimates favored rhPDGF-BB. The random-effects pooled analysis of these directly attributable comparisons suggested that treatment shortened the time to complete wound closure by approximately 4.3 days; however, the combined effect was not statistically significant and was associated with substantial uncertainty (MD: −4.28 days; 95% CI: −27.98 to 19.42; *p* = 0.262). Heterogeneity estimates were imprecise because only two studies contributed to the analysis (I^2^ = 41.8%; τ^2^ = 3.35; Q = 1.72; *p* = 0.190).

Chan et al. [23] reported time to 50% wound closure and was not combined with studies evaluating complete wound closure. Tkalčević et al. [21] stated that wounds in all experimental groups were closed by day 18 but did not report arm-specific event counts or time-to-event distributions. Rodgers et al. [25] reported no complete-closure events in either the becaplermin or placebo group by day 25 (0/5 vs. 0/5), resulting in a double-zero contrast that was uninformative for estimating a relative treatment effect. These findings were, therefore, retained in the structured descriptive synthesis.

### 2.5. Structured Descriptive Synthesis of Outcomes Not Amenable to Meta-Analysis

All 29 included studies contributed to the structured descriptive synthesis. Twenty-six studies provided at least one directly attributable comparison (D), whereas 12 provided at least one formulation-level comparison (F); nine studies contributed to both categories, yielding 38 attribution-specific rows across the modified effect-direction evidence maps. Findings already included in the quantitative syntheses were not recoded.

**Figure 7:**
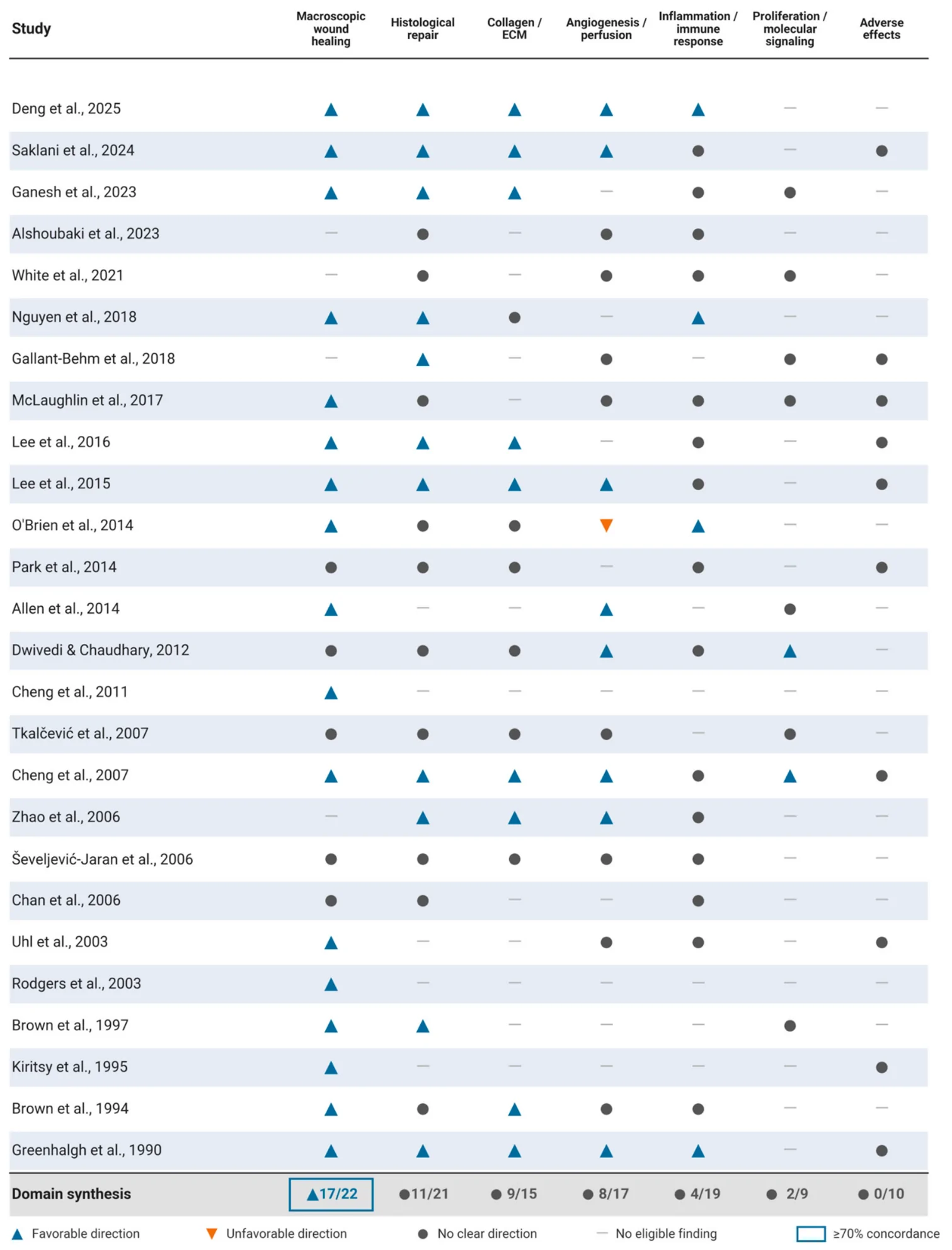
Modified effect direction evidence map for directly attributable rhPDGF-BB comparisons across the prespecified outcome domains. The studies represented in the map are cited in references [9,10,20,21,22,23,24,25,26,27,28,29,11,30,31,32,33,34,36,12,13,14,15,17,18,19]. Directly attributable comparisons were those in which the contribution of rhPDGF-BB could be evaluated independently of other formulation components. Only eligible in vivo findings not included in the quantitative synthesis were mapped. Fractions in the domain synthesis row represent the number of studies showing a favorable direction divided by the number of studies with an eligible finding. ECM, extracellular matrix; rhPDGF-BB, recombinant human platelet-derived growth factor-BB. Created in BioRender. Carrillo, J. (2026) https://BioRender.com/51mhrno.

Among directly attributable comparisons, macroscopic wound healing was evaluated in 22 studies. Seventeen studies (77.3%) showed a favorable direction [9,10,11,12,14,17,19,20,24,25,26,27,28,29,30,33,36], five (22.7%) showed no clear direction [13,15,18,22,23], and none showed an unfavorable direction (Figure 7). This proportion exceeded the prespecified exploratory 70% predominance threshold but should not be interpreted as demonstrating a statistically consistent or robust treatment effect. Four of the 22 studies (18.2%) had confirmed unit-of-analysis concerns related to non-independent wound observations [10,11,12,36], and all four belonged to the favorable subset (4/17; 23.5%). After descriptively excluding these four studies, 13 of 18 studies (72.2%) remained favorable. Three studies had a high risk of bias due to incomplete outcome data [9,11,19], and sequence generation was rated as unclear or high risk in all 22 studies. No formulation-level finding contributed to this direct 17/22 proportion; formulation-level macroscopic findings were synthesized separately. Study-specific sample sizes, experimental units, effect directions, and relevant risk-of-bias considerations are presented in Table S4. An exploratory stratification by diabetes etiology showed a favorable direction in eight of 11 studies using genetic db/db models (72.7%) and eight of 10 studies using chemically induced models (80.0%); no study in either subgroup showed an unfavorable direction. The remaining study, which used animals with pre-existing experimental diabetes but did not report the induction method, also showed a favorable direction. Because the strata were small and differed in species, wound configuration, treatment characteristics, outcome assessment, and risk of bias, they were not compared statistically. These descriptive proportions, therefore, provide no evidence of a systematic difference in treatment-effect direction according to diabetes etiology (Table 1 and Figure 7). To place these exploratory directional findings in quantitative context, two of the three study-level estimates included in the primary meta-analysis favored rhPDGF-BB, whereas one did not [9,13,29]; the pooled estimate favored treatment, but its 95% confidence interval included the null (113 animals; Figure 4). The dose-restricted sensitivity analysis retained the favorable pooled direction but remained imprecise (91 animals; Figure 5). Both studies evaluating time to complete wound closure favored rhPDGF-BB [24,27], but their pooled estimate was also highly imprecise (45 animals; Figure 6).

Angiogenesis or perfusion findings were favorable in eight of 17 directly attributable comparisons (47.1%) [9,11,14,15,19,20,21,29], showed no clear direction in eight, and were unfavorable in one [12]. Inflammation or immune response outcomes were favorable in four of 19 comparisons (21.1%) [9,12,29,33], with the remaining 15 showing no clear direction. Similarly, only two of nine comparisons assessing proliferation or molecular signaling were classified as favorable [15,19], whereas seven showed no clear direction. Consequently, none of these mechanistic domains met the threshold for a consistent direction of effect (Figure 7).

**Figure 8:**
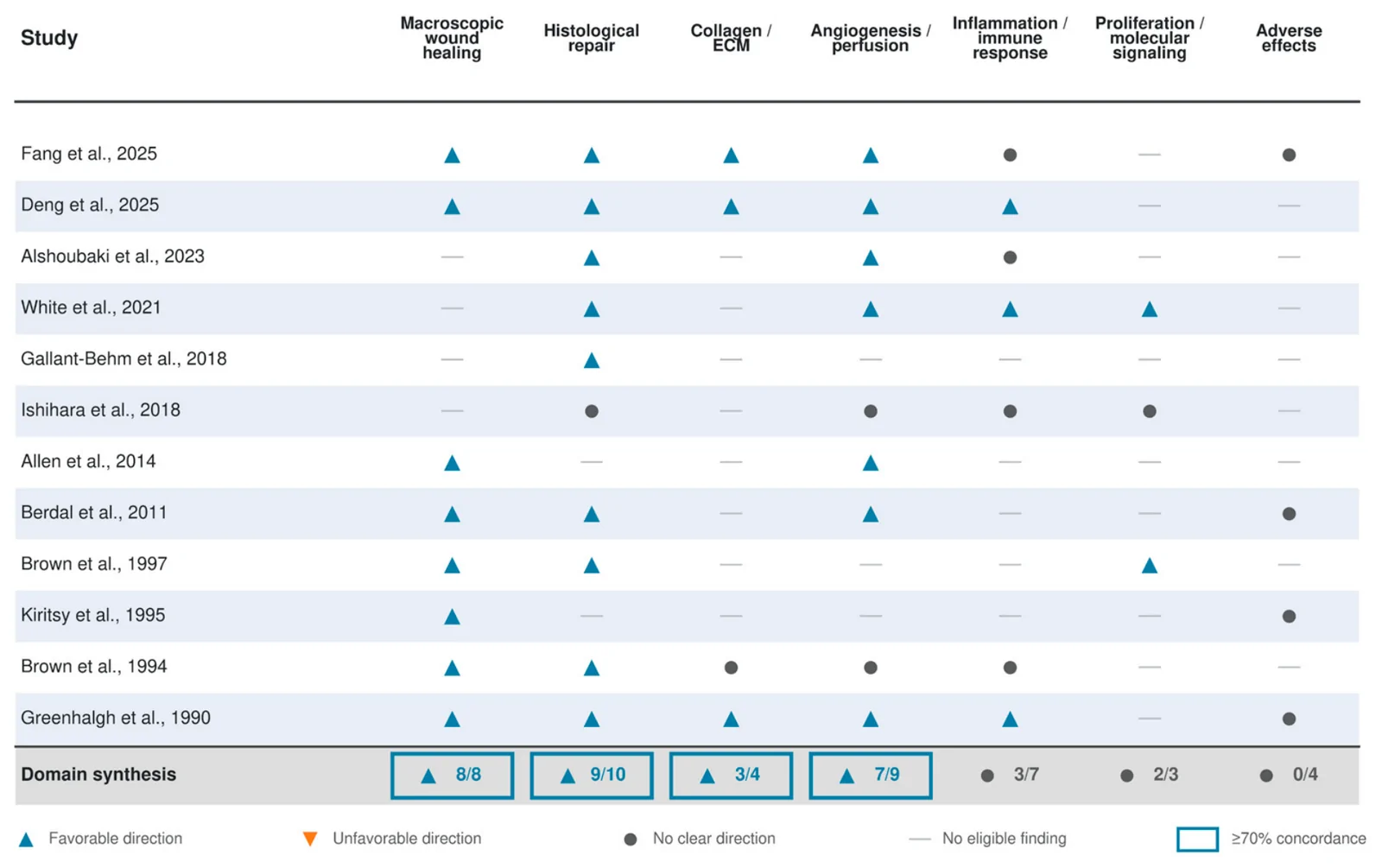
Modified effect direction evidence map for formulation-level comparisons involving rhPDGF-BB across the prespecified outcome domains. The studies represented in the map are cited in references [8,9,34,35,14,16,26,27,28,29,31,32]. These comparisons evaluated engineered rhPDGF-BB variants, rhPDGF-BB-containing delivery systems, or combinations with other biologically active interventions in which the experimental comparator did not permit isolation of the growth factor’s independent contribution. Only eligible controlled in vivo findings not included in the quantitative syntheses were mapped. Fractions in the domain synthesis row represent the number of studies showing a favorable direction divided by the number of studies with an eligible finding. Blue boxes identify domains meeting the prespecified descriptive 70% concordance threshold. Findings should be interpreted as effects of the complete intervention or formulation rather than as isolated effects of rhPDGF-BB. ECM, extracellular matrix; rhPDGF-BB, recombinant human platelet-derived growth factor-BB. Created in BioRender. Carrillo, J. (2026) https://BioRender.com/m2t5j7n.

Formulation-level comparisons showed favorable directional patterns across several reparative domains. All eight studies with eligible macroscopic outcomes showed a favorable direction [8,9,14,16,26,27,28,29]. Favorable directions were also observed for histological repair in nine of 10 studies (90.0%) [8,9,16,26,28,29,31,32,34], collagen or extracellular-matrix remodeling in three of four studies (75.0%) [8,9,29], and angiogenesis or perfusion in seven of nine studies (77.8%) [8,9,14,16,29,31,32]. These four domains met the prespecified descriptive 70% concordance threshold. In contrast, inflammation or immune-response outcomes were favorable in three of seven studies (42.9%) [9,13,36], and proliferation or molecular-signaling outcomes were favorable in two of three studies (66.7%) [26,32]; neither domain reached the threshold. Because these comparisons evaluated engineered or multicomponent interventions and did not permit isolation of the independent contribution of rhPDGF-BB, the observed directions represent effects of the complete formulation rather than isolated effects of the growth factor (Figure 8).

Adverse-effect reporting was limited. Ten directly attributable comparisons [10,11,13,19,20,24,27,29,34,36] and four formulation-level comparisons [8,16,27,29] provided an eligible safety or tolerability observation, but all were classified as showing no clear direction because no consistent between-group difference was identified. The remaining comparisons did not include an eligible in vivo adverse-effect assessment or provided insufficient reporting. Accordingly, the available evidence does not support conclusions regarding either a safety advantage or an increased risk associated with rhPDGF-BB (Figure 7 and Figure 8). An attribution-specific summary of the comparison structures, quantitative evidence, descriptive findings, and permitted interpretations is provided in Table 4.

Overall, the descriptive synthesis showed a predominantly favorable study-level pattern for macroscopic wound healing when the contribution of rhPDGF-BB could be directly evaluated. Histological, extracellular matrix, vascular, inflammatory, and molecular findings were more heterogeneous. Although multicomponent formulations showed favorable directions across several repair domains, these findings should be interpreted as formulation-level signals rather than isolated effects of rhPDGF-BB. These classifications describe effect direction only and do not incorporate effect magnitude, precision, statistical significance, risk of bias, or certainty of evidence (Figure 7 and Figure 8).

### 2.6. Certainty of the Evidence

The certainty of the evidence for percentage wound closure at days 10–12 was rated as very low (Table 5). Starting from high certainty for randomized animal experiments, the evidence was downgraded one level for serious risk of bias, one level for serious imprecision, and one level for serious indirectness. Risk-of-bias concerns primarily reflected insufficient reporting of sequence generation and allocation procedures across the three contributing studies, together with a high risk of bias due to incomplete outcome data in Deng et al.’s study [9]. These limitations were not considered very serious because Greenhalgh et al.’s study [29], which contributed 61.5% of the weight in the primary model, used coded and blinded procedures and was judged as low-risk in several outcome-relevant domains.

Although moderate statistical heterogeneity was observed (I^2^ = 54.6%), the evidence was not downgraded for inconsistency. The studies by Deng et al. [9] and Greenhalgh et al. [29] produced nearly identical favorable estimates and together accounted for 93.5% of the model weight, whereas the directionally discordant estimate from Park et al.’s study [13] was imprecise and contributed only 6.5%. Moreover, the confidence intervals of all three study estimates overlapped, and the sensitivity analysis did not reverse the direction of the pooled effect.

Imprecision was considered serious because only three studies comprising 113 animals contributed to the analysis, and the 95% confidence interval crossed the null effect, encompassing both little or no benefit and a potentially significant increase in wound closure. The wider confidence interval obtained in the sensitivity analysis further supported this judgment. Indirectness was also considered serious because all contributing evidence was derived from mouse models with experimentally created acute dorsal wounds, which do not fully reproduce the chronicity, neuropathy, ischemia, infection, and mechanical loading characteristic of human diabetic foot ulcers. Publication bias could not be assessed formally and cannot be excluded; however, no additional downgrade was applied because the comprehensive search identified both favorable and discordant findings. No upgrading factors were identified. Consequently, the available evidence is very uncertain regarding the effect of rhPDGF-BB on percentage wound closure at days 10–12.

## 3. Discussion

### 3.1. Principal Findings and Overall Interpretation

This systematic review identified a favorable but inconclusive preclinical signal from comparisons in which the contribution of rhPDGF-BB could be evaluated directly. Both quantitative syntheses were restricted to directly attributable contrasts in which rhPDGF-BB or an rhPDGF-BB-containing intervention was compared with a factor-free control, including a matched carrier when applicable. The primary meta-analysis numerically favored the rhPDGF-BB-containing intervention for percentage wound closure at days 10–12; however, the confidence interval included the possibility of no effect, and the estimate was derived from only three mouse studies [9,13,29]. In the structured descriptive synthesis, macroscopic wound healing was the only directly attributable domain in which the proportion of favorable study-level classifications exceeded the prespecified exploratory 70% concordance threshold (17 of 22 studies; Figure 7; Table S4), whereas histological repair, extracellular matrix remodeling, angiogenesis, inflammation, and molecular signaling showed more heterogeneous patterns. Formulation-level comparisons showed favorable patterns across four reparative domains, but these findings apply to the complete interventions and cannot be attributed specifically to rhPDGF-BB (Table 4). Taken together, the directly attributable evidence supports possible biological activity of rhPDGF-BB in diabetic wounds, but its very low certainty precludes firm conclusions regarding the magnitude, reproducibility, or translational relevance of the effect. This uncertainty is compounded by substantial translational indirectness because the quantitative evidence was derived exclusively from experimentally created acute dorsal wounds in mice, predominantly without mechanical splinting, and these models do not reproduce the chronicity, neuropathy, ischemia, infection, repetitive mechanical loading, or polymicrobial biofilm characteristic of human diabetic foot ulcers.

The apparent contrast between the favorable direction observed across many studies and the inconclusive pooled estimate is not contradictory. The effect direction synthesis captured whether eligible findings favored the intervention, but did not account for effect magnitude, precision, statistical significance, sample size, or risk of bias. Consequently, a broadly favorable directional pattern can coexist with substantial quantitative uncertainty. Similarly, the more extensive favorable pattern observed with multicomponent formulations cannot be attributed solely to rhPDGF-BB, because biomaterial properties, delivery kinetics, and additional active components may have contributed to the observed effects. The two studies evaluating time to complete wound closure also used directly attributable contrasts and produced estimates favoring rhPDGF-BB relative to their corresponding factor-free carrier controls, but the pooled effect remained highly imprecise [24,27]. Moreover, the absence of a consistent difference in adverse effects should not be interpreted as evidence of equivalent safety, given the limited and heterogeneous reporting of tolerability outcomes.

This cautious interpretation is consistent with the clinical literature. A PDGF-specific meta-analysis of six randomized trials involving 992 participants reported a higher probability of complete healing with topical rhPDGF than with placebo or standard care [37]. A more recent network meta-analysis also found that PDGF increased the likelihood of complete healing; however, this effect was not reproduced when the analysis was restricted to trials at low risk of bias [38]. Accordingly, the 2023 International Working Group on the Diabetic Foot guideline suggested against the routine use of growth factor therapy as an adjunct to standard care, issuing a conditional recommendation based on low-certainty evidence [5]. The preclinical and clinical evidence, therefore, converge on a similar pattern: an overall favorable therapeutic signal whose strength is diminished by methodological limitations, heterogeneity, imprecision, and insufficient safety assessment. The present findings should, consequently, be viewed as a basis for refining mechanistic hypotheses, delivery strategies, animal models, and future trial designs rather than as direct support for a clinical treatment recommendation.

### 3.2. Macroscopic Wound Healing and Interpretation of the Therapeutic Effect

Macroscopic wound healing emerged as the clearest directional efficacy signal within the directly attributable evidence. Favorable responses were reported across more than three decades of experimental research, ranging from direct comparisons of topically administered recombinant growth factor in genetically diabetic mice [29] and hyperglycemic animals with impaired microcirculation [24] to a matched-carrier comparison in which a PDGF-BB-functionalized sericin hydrogel was evaluated against the corresponding factor-free sericin hydrogel [9]. This breadth suggests directly attributable macroscopic activity across different diabetic models and delivery contexts; it does not imply that findings from unmatched multicomponent formulations can be ascribed to rhPDGF-BB alone. Nevertheless, wound closure is an integrative endpoint that may reflect different combinations of wound contraction, re-epithelialization, granulation tissue formation, and tissue remodeling. An improvement in macroscopic closure, therefore, does not necessarily indicate complete structural or functional restoration of the injured tissue.

The mechanism by which rodent wounds close is particularly important when interpreting these findings. Excisional wounds in mice and rats heal predominantly through contraction mediated by the panniculus carnosus, whereas human cutaneous wounds rely more heavily on granulation-tissue formation and re-epithelialization. Silicone splinting was developed to minimize contraction and thereby produce a repair process that more closely resembles human wound healing [39]. However, only four studies included in the present review explicitly used mechanical splinting. The absence of an accelerated healing response in the splinted model reported by Park et al. [13] raises the possibility that contraction may have amplified some favorable effects observed in non-splinted wounds. Nevertheless, favorable macroscopic responses were also observed in other splinted models in which becaplermin was evaluated as an active comparator or as part of a combination strategy [14,33]. Splinting should, therefore, be considered an important effect modifier rather than a binary indicator of model validity, and variation in contraction control probably contributed to the heterogeneity between studies.

A structured comparison of the four studies that explicitly used mechanical splinting [13,14,32,33] provided mixed findings. Three contributed eligible macroscopic wound-healing evidence: favorable directions were observed in Nguyen et al. [33] and Allen et al. [14], whereas Park et al. [13] showed no clear direction; White et al. [32] did not contribute an eligible macroscopic finding to the effect direction synthesis. In the quantitative sensitivity analysis, excluding non-splinted models left only Park et al. [13], whose individual estimate did not favor rhPDGF-BB and remained imprecise (MD: −3.60 percentage points; 95% CI: −19.59 to 12.39). Consequently, a splinted-only pooled estimate could not be calculated. The available splinted evidence is, therefore, insufficient to determine whether contraction control modifies the efficacy of rhPDGF-BB.

The underlying diabetes model provides another source of biological variability. A systematic review and network meta-analysis of mouse models found that db/db mice exhibited the greatest impairment in wound healing, although STZ-induced, STZ-plus-high-fat-diet, and ob/ob models also showed delayed closure. Age, sex, and wound configuration additionally influenced the magnitude of healing impairment [40]. In the present review, exploratory study-level directions for directly attributable macroscopic healing were broadly similar between genetic db/db and chemically induced models (Table 1 and Figure 7). However, the small number of studies and the heterogeneity in species, wound characteristics, interventions, and outcome assessments precluded a formal comparison, and this descriptive similarity should not be interpreted as evidence of equivalent efficacy across diabetic phenotypes. Most experiments used one of these two model categories and created acute full-thickness dorsal wounds. Only one study used a porcine model, which offers greater anatomical and physiological similarity to human skin [12]. Although these models reproduce selected consequences of diabetes, they generally do not reproduce the prolonged disease duration, repetitive mechanical loading, neuropathy, ischemia, infection, and polymicrobial biofilm characteristic of chronic human diabetic foot ulcers. Recent systematic evidence, therefore, supports combining metabolic dysfunction with ischemic, infectious, or mechanically relevant wound conditions and validating promising interventions in species whose repair mechanisms more closely resemble those of humans [41].

Accordingly, the favorable direction observed in the directly attributable macroscopic evidence should be regarded as the strongest preclinical efficacy signal for rhPDGF-BB, but not as a demonstration of a precise treatment effect or of translational effectiveness. Future studies should standardize wound dimensions, contraction control, photographic acquisition, planimetric analysis, and assessment time points; report the independent experimental unit clearly; and complement percentage closure with measures of re-epithelialization, granulation tissue quality, tensile integrity, and sustained healing. Confirmation in chronic composite models and subsequent validation in porcine or other human-relevant systems would provide a more rigorous basis for determining whether the observed acceleration of closure represents meaningful tissue repair.

### 3.3. Histological Repair and Extracellular Matrix Remodeling

Histological repair and extracellular matrix remodeling showed less directional concordance than macroscopic wound closure within the directly attributable evidence. Favorable directions were observed in 11 of 21 studies for histological repair [9,10,11,19,20,21,26,29,30,33,34] and in nine of 15 studies for collagen or extracellular matrix remodeling [9,10,11,19,20,21,28,29,30]. In separate formulation-level comparisons, favorable directions were observed in nine of 10 studies for histological repair [8,9,16,26,28,29,31,32,34] and in three of four studies for collagen or extracellular matrix remodeling [8,9,29]; however, these findings apply to the complete formulations and do not isolate the independent contribution of rhPDGF-BB. Although individual experiments reported improved re-epithelialization, granulation-tissue formation, collagen deposition, or matrix organization [9,10,11,19,28,29], the lower concordance in the directly attributable evidence indicates that faster macroscopic closure did not uniformly translate into restoration of an organized and mature dermal architecture. This distinction is important because wound area reduction can precede the development of mechanically competent tissue and may not capture abnormalities in epithelial integrity, collagen organization, matrix turnover, or scar durability.

The histological effects of rhPDGF-BB are plausibly mediated through the recruitment and expansion of repair-associated mesenchymal cells. Activation of PDGF receptors, particularly PDGFR-β in fibroblasts and pericytes, promotes cellular migration and proliferation during early wound repair. Experimental inhibition of PDGFR-β delays closure and reduces fibroblast recruitment, myofibroblast abundance, fibronectin deposition, and type I collagen-producing cells [42]. Diabetes disrupts this response through persistent oxidative and inflammatory stress, impaired fibroblast migration and survival, and defective transition toward a reparative myofibroblast phenotype. A recent systematic review showed that the diabetic wound environment interferes with TGF-β/SMAD and other pathways required for myofibroblast function and collagenization [43]. Exogenous rhPDGF-BB may, therefore, partially restore the cellular input required for granulation tissue and matrix formation, but its effect remains dependent on the availability and functional competence of responsive fibroblast populations.

Matrix restoration also requires more than increased collagen accumulation. Fibronectin forms an essential provisional scaffold for fibroblast migration, collagen assembly, and growth factor sequestration, but its deposition is impaired, and its degradation predominates in the proteolytic environment of diabetic foot ulcers [44]. During normal remodeling, the provisional matrix is progressively replaced by organized collagen, with coordinated degradation of damaged matrix and maturation of newly synthesized fibers. In diabetic wounds, excessive matrix metalloproteinase activity can shift this balance toward persistent degradation. Clinically, elevated wound-fluid MMP-9 and an increased MMP-9-to-TIMP-1 ratio have been associated with slower healing and a lower probability of complete diabetic foot ulcer closure [45]. Consequently, increased collagen staining at a single experimental time point cannot by itself establish successful remodeling; collagen composition, alignment, cross-linking, degradation, and tensile function must also be considered.

Interpretation is further complicated by the characteristics of the delivery systems used in several included studies. PLGA–collagen scaffolds, nanofibrous membranes, sericin hydrogels, fibrin matrices, and other biomaterials may independently provide structural support, cell adhesion sites, moisture control, or protection from proteolytic degradation [9,10,11]. Histological improvements observed with these platforms may, therefore, reflect a combined interaction between rhPDGF-BB, the carrier matrix, and the local wound environment rather than the isolated activity of the growth factor. This does not diminish their therapeutic relevance, but it changes the level of attribution: the resulting tissue response should be interpreted as a formulation-level effect unless an otherwise equivalent carrier lacking rhPDGF-BB was evaluated.

Future studies should combine standardized histomorphometry with quantitative assessment of epithelial continuity, granulation-tissue volume, fibronectin, collagen types I and III, fiber alignment and maturity, MMP/TIMP balance, and biomechanical strength. Evaluations at multiple repair stages would help distinguish an early increase in the provisional matrix from durable extracellular matrix restoration. Such approaches are necessary to determine whether rhPDGF-BB merely accelerates tissue filling or promotes the formation of structurally and functionally competent, repaired skin.

### 3.4. Angiogenesis, Immune Regulation, and PDGFR-Dependent Signaling

The mechanistic domains showed substantially less directional consistency than macroscopic wound healing. Among directly attributable comparisons, angiogenesis or perfusion showed a favorable direction in eight of 17 studies [9,11,14,15,19,20,21,29], no clear direction in eight [18,22,24,28,31,32,34,35], and an unfavorable direction in one [12]. Inflammation or immune response outcomes were favorable in four of 19 studies [9,12,29,33], whereas the remaining 15 showed no clear direction [10,11,13,15,19,20,21,22,23,24,28,30,31,32,35]. Proliferation or molecular-signaling outcomes were favorable in only two of nine studies [15,19], with no clear direction in the other seven [14,18,26,30,32,34,35]. Among formulation-level comparisons, favorable directions were observed for angiogenesis or perfusion in seven of nine studies [8,9,14,16,29,31,32], with no clear direction in two [28,35]; for inflammation or immune responses in three of seven studies [9,29,32], with no clear direction in four [8,28,31,35]; and for proliferation or molecular signaling in two of three studies [26,32], with no clear direction in one [35]. These findings apply to the complete formulations and cannot establish pathway-specific effects attributable independently to rhPDGF-BB. The lower concordance observed as the assessment moved from wound-level repair to specific biological pathways argues against a single linear mechanism and instead supports a context-dependent network effect. The study-level findings summarized above constitute the mechanistic evidence provided by the included experiments; the receptor- and pathway-level framework discussed below is derived primarily from the external biological literature and is used to interpret, rather than confirm, these heterogeneous findings.

The heterogeneity of the vascular findings is biologically plausible because PDGF-BB should not be interpreted simply as another endothelial sprouting factor. While VEGF predominantly initiates endothelial cell activation, permeability, migration, and vascular sprouting, PDGF-BB has a particularly important role in recruiting PDGFR-β-expressing pericytes and vascular smooth-muscle cells to nascent endothelial tubes. It also contributes to perivascular cell proliferation, basement membrane deposition, and vascular stabilization and maturation [46,47]. Consequently, an increased capillary bud density or CD31-positive area does not necessarily indicate that newly formed vessels are mature or functionally perfused. Conversely, improved vascular stability or tissue perfusion may occur without a large increase in vessel number. Studies assessing functional microcirculation, such as that by Uhl et al. [24], may, therefore, provide information that is more biologically informative than vascular density alone.

The diabetic wound environment may further limit the vascular response to rhPDGF-BB. Diabetes impairs HIF-1α stabilization and the expression of downstream angiogenic mediators, including VEGF, while also disrupting endothelial cell function and the mobilization and trafficking of vascular progenitor cells [48]. PDGF-BB cannot independently compensate for all these abnormalities. Rather, the available evidence supports a coordinated and partially overlapping model in which endothelial sprouting signals establish immature vascular networks and PDGF-dependent recruitment of perivascular cells subsequently contributes to their stabilization and functional maturation. The improvement in progenitor cell trafficking and neovascularization observed when PDGF-BB was combined with AMD3100 [14], together with favorable formulation-level findings from multi-growth-factor systems [9,29,32], is consistent with this cooperative model. However, these combination studies cannot establish whether the broader response resulted from PDGF-BB itself, the accompanying therapeutic component, altered local bioavailability, or interaction among these mechanisms. Similarly, the single unfavorable vascular direction should be interpreted in the context of its specific comparator and experimental design [12] and does not, in isolation, demonstrate an antiangiogenic effect of rhPDGF-BB.

The inflammatory findings require similar caution. Successful repair depends on a transient inflammatory response followed by resolution and transition toward a reparative environment. Diabetic wounds instead exhibit delayed or persistent neutrophil accumulation, dysfunctional neutrophil extracellular trap formation, impaired efferocytosis, and the coexistence of inflammatory and reparative macrophage populations with altered functions [49]. Recent experimental evidence also demonstrates that diabetic keratinocytes can sustain macrophage NLRP3 inflammasome activity through an IL-1α/IL-1 receptor/JMJD3-dependent pathway, illustrating the importance of intercellular signaling in maintaining unresolved inflammation [50]. Against this complex background, rhPDGF-BB should not be regarded as a dedicated anti-inflammatory agent. Any reduction in inflammatory markers may be secondary to accelerated tissue repair, improved vascularization, reduced tissue damage, or the biological activity of a carrier or co-administered agent. This interpretation is supported by the predominance of no-clear-direction classifications in the directly attributable comparisons [10,11,13,15,19,20,21,22,23,24,28,30,31,32,35] and by the more favorable, but non-attributable, inflammatory signals observed in multicomponent formulations [9,29,32].

Evidence from the broader receptor biology literature indicates that PDGF-BB binding induces dimerization and autophosphorylation of PDGFR-containing receptor complexes, generating docking sites for signaling proteins that activate pathways including Ras–MAPK/ERK, PI3K–Akt, phospholipase C-γ, and Src-family networks [46]. These pathways can promote cell migration, proliferation, survival, and matrix production. Cheng et al. [19] provided the clearest direct evidence of target-related signaling among the included studies by demonstrating increased ERK phosphorylation, c-Fos expression, and proliferating cell nuclear antigen labeling after rhPDGF treatment in diabetic wounds. Nevertheless, only two directly attributable comparisons showed a favorable direction for proliferation or molecular signaling [15,19], whereas the remaining seven showed no clear direction [14,18,26,30,32,34,35]. Most studies measured isolated downstream markers at a single time point, frequently in whole-wound tissue, without confirming PDGFR activation or identifying the responding cell population. The mechanistic plausibility of PDGF signaling is, therefore, strong, but its activation within the diabetic wound environment was not comprehensively demonstrated across the included evidence.

Taken together, the included experiments do not establish uniform activation of PDGFR-dependent pathways. When interpreted alongside the broader mechanistic literature, the heterogeneous findings are compatible with a model in which rhPDGF-BB may act as a context-dependent amplifier and coordinator of repair rather than as a universal switch for angiogenesis or inflammation. When the wound bed is adequately perfused, debrided, and controlled for infection, PDGF-dependent signaling may enhance fibroblast and perivascular cell recruitment, granulation tissue formation, extracellular matrix deposition, and vascular maturation. In contrast, severe hypoxia, persistent inflammation, oxidative stress, excessive protease activity, and impaired endogenous angiogenic signaling may reduce growth factor bioavailability or cellular responsiveness. Delivery systems and combination therapies may partially overcome these barriers, but their effects must be separated experimentally from those of rhPDGF-BB.

Future studies should, therefore, incorporate temporal and cell-specific mechanistic assessments. These should include PDGFR phosphorylation and downstream ERK and Akt activation; localization of signaling to fibroblasts, pericytes, endothelial cells, and immune populations; functional perfusion measurements; pericyte coverage and vascular permeability; macrophage phenotypes and efferocytosis; and neutrophil persistence and extracellular trap formation. Factorial designs with matched carriers and individual therapeutic components will also be required to distinguish PDGF-specific activity from formulation-level synergy. Such an approach would connect molecular target engagement to tissue-level repair and provide a stronger basis for translating favorable preclinical directions into clinically meaningful effects.

### 3.5. Formulation, Local Bioavailability, and Combination Strategies

One of the most informative findings of this review was the distinction between directly attributable and formulation-level evidence. After applying the attribution framework at the comparison level, 12 studies contributed at least one formulation-level comparison [8,9,14,16,26,27,28,29,31,32,34,35]; nine of these also contained directly attributable comparisons, whereas three contributed formulation-level evidence exclusively. All eight studies with eligible macroscopic outcomes showed a favorable direction [8,9,14,16,26,27,28,29]. Favorable directions were also observed for histological repair in nine of 10 studies (90.0%) [8,9,16,26,28,29,31,32,34], collagen or extracellular matrix remodeling in three of four studies (75.0%) [8,9,29], and angiogenesis or perfusion in seven of nine studies (77.8%) [8,9,14,16,29,31,32]. In contrast, inflammation or immune response outcomes were favorable in three of seven studies (42.9%) [9,29,32], and proliferation or molecular-signaling outcomes were favorable in two of three studies (66.7%) [26,32]. These directional patterns apply to the complete engineered or multicomponent interventions and do not demonstrate greater intrinsic efficacy of rhPDGF-BB, because the independent contribution of the growth factor could not be isolated, and the direct and formulation-level syntheses involved different interventions, comparators, delivery systems, and experimental contexts.

Local bioavailability is likely to be a major determinant of the response to rhPDGF-BB. Recombinant growth factors administered in soluble or conventional topical form have relatively short biological persistence and may be degraded by the protease-rich chronic-wound environment, inactivated through oxidation, removed by wound exudate, or distributed away from their intended cellular targets. Bolus administration can also produce a high initial concentration followed by rapid decline rather than the sustained spatial gradient required for cell recruitment and coordinated tissue repair [51]. Hydrogels, nanofibrous membranes, polymeric scaffolds, and extracellular-matrix-binding strategies may protect the protein, prolong its residence within the wound bed, and control its release over time [51,52]. Therefore, the nominal dose of rhPDGF-BB does not necessarily represent the biologically available dose received by target cells.

The marked heterogeneity in dose reporting also prevents definition of a universal preclinical reference range. Among studies reporting an absolute active amount, rhPDGF-BB exposure ranged from 50 ng per application to 20 µg per wound per day, whereas other studies used area-normalized doses of 4, 7, or 40 µg/cm^2^; several reports lacked sufficient information to reconstruct the active mass delivered per wound. Because a standardized equivalent cumulative dose could not be calculated across studies, differences in total exposure, administration frequency, treatment duration, and release kinetics may have contributed unaccounted between-study variability in the observed effect sizes. This potential source of heterogeneity could not be examined through dose-based subgroup analysis or meta-regression because only three studies contributed to the primary meta-analysis, and exposure was incompletely or non-comparably reported. As a clinical reference, the pivotal phase III trial evaluated becaplermin gel at 30 or 100 µg/g, applied once daily together with standardized wound care until complete closure or for a maximum of 20 weeks; the 100 µg/g formulation, equivalent to 0.01%, was the dose that significantly improved complete closure relative to the placebo [4]. Accordingly, future studies evaluating free rhPDGF-BB or conventional gel should include a clinically anchored 0.01% becaplermin reference arm, normalize exposure by wound area, and report both µg/wound and µg/cm^2^. Dose–response experiments should include lower and higher exposures when appropriate. However, a single nominal range should not be imposed on controlled-release carriers or engineered proteins because loading efficiency, release kinetics, local retention, degradation, and retained bioactivity can substantially modify effective tissue exposure.

Several included studies illustrate how the delivery platform can alter therapeutic performance. Fang et al. incorporated rhPDGF-BB into a conductive, thermosensitive polypyrrole-based hydrogel and reported wound-healing activity comparable to Regranex within their diabetic mouse model while using approximately 1/200 of the rhPDGF-BB dose [8]. Deng et al. used a silk-sericin hydrogel containing both EGF and PDGF-BB to provide sustained growth factor release together with a biologically supportive matrix [9]. White et al. engineered VEGF-A, PDGF-BB, and HB-EGF to bind with high affinity to extracellular matrix components, thereby increasing local retention and producing a broader response when the three engineered factors were administered together [32]. Similarly, Alshoubaki et al. demonstrated that engineering PDGF-BB with a high-affinity extracellular-matrix-binding motif prolonged tissue retention, reduced systemic leakage, and enhanced regenerative activity compared with the unmodified protein [31]. Collectively, these studies indicate that retention, spatial presentation, and release kinetics may be as important as the administered concentration.

The carrier itself may also contribute biologically to the observed response. Natural and synthetic matrices can maintain wound moisture, absorb exudate, provide structural support for cellular infiltration, interact with extracellular matrix proteins, and modify local mechanical properties. Some formulations additionally possess conductive, antioxidant, antimicrobial, or immunomodulatory activity. Accordingly, a favorable comparison involving rhPDGF-BB within an active hydrogel or scaffold may reflect the combined effects of growth factor signaling, material properties, and wound microenvironment modulation rather than PDGF-BB alone. This distinction is especially important when the experimental comparator is untreated tissue, saline, or a different commercial formulation rather than an identical carrier lacking only rhPDGF-BB.

Combination strategies introduce an additional level of complexity. Different growth factors regulate overlapping but nonidentical components of repair, creating a biological rationale for combining fibroblast recruitment, epithelialization, angiogenic sprouting, and vascular maturation. However, combination does not automatically imply synergy. Kiritsy et al. found that PDGF-BB combined with insulin-like growth factor-I was more effective than PDGF-BB alone in promoting complete healing in older diabetic mice [27], while Brown et al. reported synergistic repair with PDGF-BB and transforming growth factor-α [28]. In contrast, Greenhalgh et al. found that PDGF-BB combined with basic fibroblast growth factor improved healing parameters but did not outperform either growth factor administered alone [29]. These findings indicate that the effect of a combination depends on the biological partner, dose ratio, temporal sequence, wound model, and outcome assessed.

Current delivery research is moving from simple sustained release toward stage-responsive and sequential therapeutic systems. For example, a recent hierarchical hydrogel was designed to release PDGF-BB and DNase I with different kinetics, combining early growth factor activity with degradation of excessive neutrophil extracellular traps in diabetic wounds [53]. Although such studies do not establish the isolated efficacy of rhPDGF-BB and remain preclinical, they illustrate a potentially important principle: a growth factor may be more effective when its delivery is coordinated with correction of the hostile wound microenvironment rather than administered as a single uninterrupted signal.

Future studies should, therefore, treat the formulation as an experimental variable rather than as a neutral vehicle. At minimum, factorial designs should include an untreated or standard-care group, the carrier alone, free rhPDGF-BB, carrier-delivered rhPDGF-BB, each additional active component alone, and the complete combination. Formulations should also be characterized for loading efficiency, release kinetics under wound-relevant conditions, retained protein bioactivity, degradation, local residence time, systemic exposure, material-related toxicity, sterilization stability, and batch reproducibility. Measurement of PDGFR target engagement would help determine whether prolonged retention results in sustained biological signaling rather than merely prolonged protein detection.

Overall, the formulation-level evidence identifies drug delivery as a central modifier of the rhPDGF-BB response. Advanced carriers may improve stability, localization, and temporal presentation and may simultaneously correct other components of the diabetic wound microenvironment. Nevertheless, these systems constitute distinct therapeutic entities and should not be considered interchangeable with free rhPDGF-BB or conventional becaplermin gel. Separating growth-factor-specific activity from carrier effects and true combination synergy will be essential for identifying which preclinical formulations warrant clinical translation.

### 3.6. Safety, Adverse-Effect Reporting, and Clinical Relevance

Safety reporting was substantially less informative than efficacy reporting. Ten directly attributable comparisons provided at least one eligible safety or tolerability observation [10,11,13,19,20,24,27,29,34,36], and four formulation-level comparisons contributed such information [8,16,27,29]. All eligible observations were classified as showing no clear direction because they did not demonstrate a consistent between-group difference. For directly attributable comparisons, this classification does not establish either the safety of rhPDGF-BB or an increased risk attributable to the growth factor. For formulation-level comparisons, it likewise does not establish the safety of the complete formulation or of any individual component. Rather, the available observations were insufficient to determine either an increased risk or a safety advantage at either level of attribution. Study-level details regarding the safety and tolerability variables assessed, follow-up duration, reported adverse events, attrition and exclusions, systemic evaluations, and permitted level of attribution are provided in Table S2.

The strength of this conclusion is limited by the design of the included experiments. Most studies used relatively small cohorts, short observation periods, and follow-ups centered primarily on wound closure or tissue repair. Safety outcomes were seldom prespecified or evaluated using standardized definitions, and systematic assessments of application-site reactions, infection, systemic toxicity, laboratory abnormalities, biodistribution, immunogenicity, or pathological proliferative responses were generally incomplete [8,10,11,13,16,19,20,24,27,29,34,36]. Consequently, the experiments may have been capable of detecting overt acute toxicity but were insufficiently powered to identify uncommon, delayed, exposure-dependent, or organ-specific adverse effects.

Because PDGF-BB is a potent mitogen for mesenchymal cells and supports extracellular matrix production, sustained or spatially uncontrolled PDGF/PDGFR signaling could theoretically contribute to excessive or persistent granulation tissue formation, pathological fibrosis, or tumor-promoting activity in susceptible tissues [46]. These outcomes were not demonstrated by the included animal studies, and their biological plausibility should not be interpreted as evidence that topical rhPDGF-BB is oncogenic; however, the available study designs and follow-up periods were insufficient to exclude uncommon or delayed proliferative effects.

Clinical evidence provides cautious reassurance regarding conventional topical becaplermin. A matched cohort study including 1622 becaplermin initiators and 2809 matched comparators found no statistically significant increase in overall cancer incidence, while extended follow-up did not demonstrate an increase in overall cancer mortality; however, estimates among patients with greater cumulative exposure remained imprecise [54]. Subsequent postmarketing investigations contributed to the removal, in 2018, of the boxed warning concerning cancer-related mortality from the prescribing information [55]. These findings reduce concern regarding a major population-level carcinogenic signal but do not establish the absence of rare risks or equivalent safety across all clinical circumstances.

The clinical relevance of these observations must also be considered within the authorized and evidence-based context of use. Conventional becaplermin is intended as an adjunct—not a substitute—for appropriate ulcer care in adequately perfused lower-extremity diabetic neuropathic ulcers extending into the subcutaneous tissue or beyond. Its use, therefore, remains dependent on adequate debridement, pressure offloading, infection control, moist wound care, and assessment and correction of vascular insufficiency when indicated [55,56]. Moreover, current IWGDF guidance suggests against the routine use of growth factor therapy for diabetes-related foot ulcers because the supporting clinical efficacy evidence remains uncertain and predominantly of low certainty [5]. Reassessment of a historical safety concern should, therefore, not be interpreted as strengthening the evidence for therapeutic effectiveness [5,38].

Importantly, the clinical safety experience with the conventional carboxymethylcellulose-based becaplermin gel cannot be directly extrapolated to engineered extracellular-matrix-binding proteins, conductive or biodegradable hydrogels, nanofibrous scaffolds, or multicomponent growth factor formulations [8,9,10,11,31,32,35]. These systems may alter local retention, tissue penetration, degradation kinetics, cumulative exposure, and systemic distribution. Their safety assessment should consequently address not only rhPDGF-BB but also carrier toxicity, degradation products, local irritation or sensitization, immunogenicity, systemic leakage, infection or biofilm formation, excessive fibrosis, ectopic angiogenesis, and unintended proliferative responses. This distinction is particularly relevant because PDGF receptor signaling regulates cellular proliferation, migration, and vascular remodeling, and its biological consequences depend on dose, spatial distribution, cellular context, and duration of receptor activation [46,55].

Future preclinical studies should incorporate prespecified and standardized safety outcomes alongside efficacy endpoints. Relevant assessments include serial clinical monitoring, application-site scoring, hematological and biochemical testing, blinded histopathological examination of the wound and major organs, systemic pharmacokinetic and biodistribution analyses, evaluation of antidrug antibodies when appropriate, and follow-up extending beyond wound closure. Advanced formulations should additionally be evaluated using formulation-matched controls and sufficiently long observation periods to detect delayed inflammatory, fibrotic, angiogenic, or proliferative abnormalities. Overall, the available evidence is insufficient to establish either the safety of rhPDGF-BB or the absence of safety concerns, particularly under repeated dosing or prolonged follow-up. This limitation is even greater for newer delivery systems and combination products, whose complete formulations may alter local and systemic exposure.

### 3.7. Translational Relevance and Mechanistic Integration

**Figure 9:**
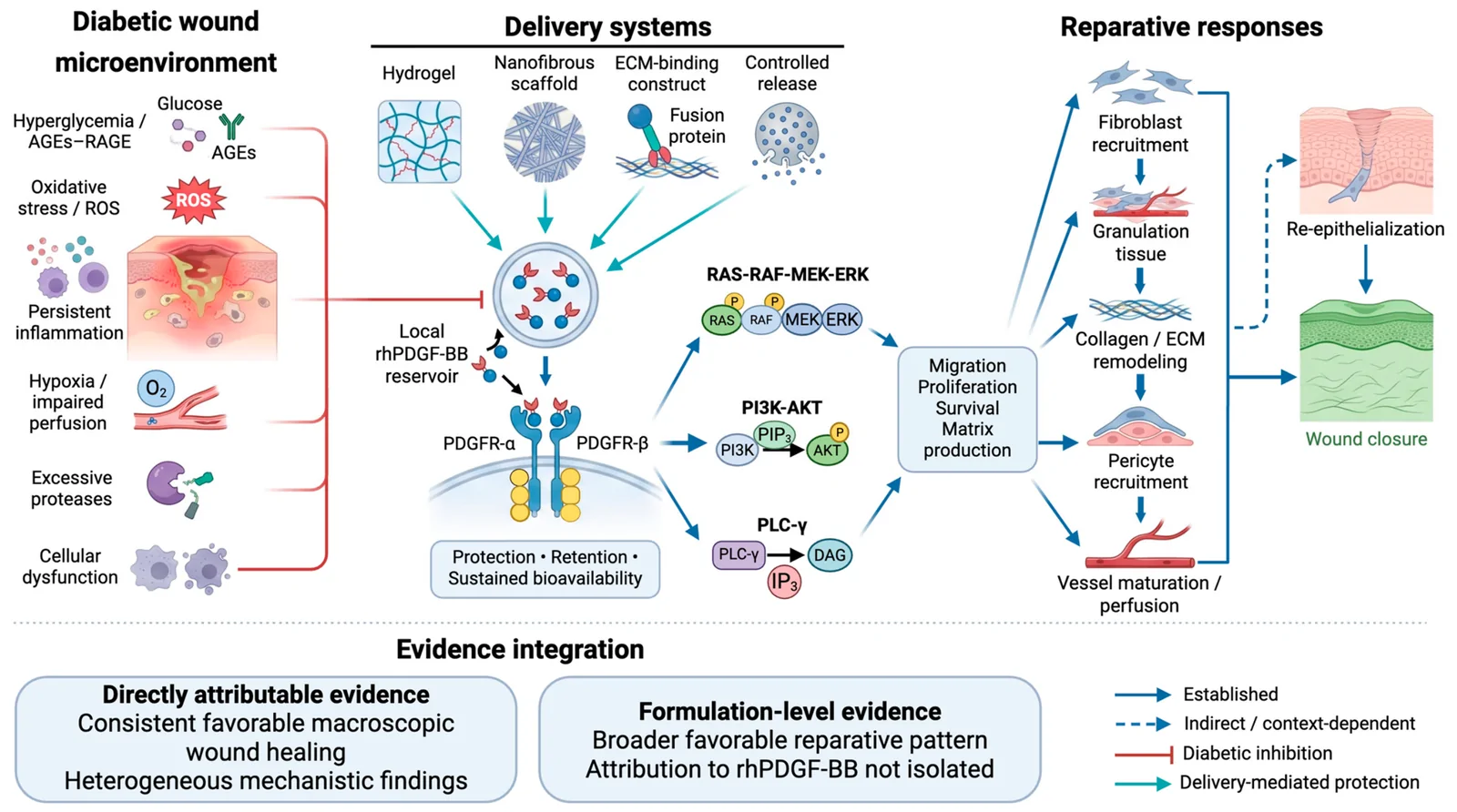
The schematic integrates the biological constraints imposed by the diabetic wound microenvironment, the potential protective role of delivery systems, and PDGFR-mediated signaling with the reparative responses identified in this review. Directly attributable evidence showed a predominantly favorable but exploratory study-level pattern for macroscopic wound healing, whereas mechanistic findings were heterogeneous. Formulation-level evidence suggested broader reparative activity but did not permit isolation of the specific contribution of rhPDGF-BB. The model combines findings from the included studies with established pathway biology from the external literature and should, therefore, be interpreted as an evidence-informed conceptual framework; not every depicted relationship was directly evaluated in the reviewed experiments, and uniform pathway activation was not demonstrated. AGEs, advanced glycation end-products; RAGE, receptor for advanced glycation end-products; ROS, reactive oxygen species; ECM, extracellular matrix; rhPDGF-BB, recombinant human platelet-derived growth factor-BB; PDGFR, platelet-derived growth factor receptor; PI3K, phosphoinositide 3-kinase; AKT, protein kinase B; PLC-γ, phospholipase C gamma; PIP_3_, phosphatidylinositol 3,4,5-trisphosphate; DAG, diacylglycerol; IP_3_, inositol 1,4,5-trisphosphate. Created in BioRender. Carrillo, J. (2026) https://BioRender.com/l40l5v0.

The translational interpretation of rhPDGF-BB requires consideration of the biological environment into which the growth factor is delivered. A diabetic foot ulcer does not represent the isolated absence of a single reparative mediator but rather a spatially and temporally heterogeneous tissue state characterized by hyperglycemia, advanced glycation end-products, oxidative stress, persistent or dysregulated inflammation, fibroblast dysfunction, impaired angiogenesis, extracellular matrix disorganization, and excessive proteolytic activity [57,58,59,60,61]. Human single-cell and spatial transcriptomic analyses further demonstrate that healing and nonhealing diabetic ulcers contain distinct fibroblast, endothelial, keratinocyte, and immune cell states, emphasizing that therapeutic responsiveness is likely determined by the composition and functional condition of the wound microenvironment [58]. These abnormalities can simultaneously increase the need for reparative signaling and reduce the ability of resident cells to respond appropriately to an exogenously administered growth factor (Figure 9).

The established receptor biology literature indicates that rhPDGF-BB binds to PDGFR-α and PDGFR-β, inducing receptor homo- or heterodimerization and tyrosine autophosphorylation. The resulting signaling can engage pathways including RAS–RAF–MEK–ERK, PI3K–AKT, and phospholipase C-γ, which regulate cellular migration, proliferation, survival, and matrix production [46,57,59]. In fibroblasts, these signals support chemotaxis into the wound bed, expansion of the reparative stromal compartment, granulation tissue formation, and collagen and extracellular matrix deposition. In pericytes and other perivascular mesenchymal cells, PDGFR-dependent signaling contributes to vascular support cell recruitment and vessel stabilization, thereby complementing endothelial angiogenic responses [46,47]. Improved stromal support, vascular maturation, and matrix organization can subsequently facilitate re-epithelialization and macroscopic closure, although these downstream effects are likely to be partly indirect and dependent on the surrounding cellular context [9,10,11,14,15,19,20,21,26,28,29,30,34] (Figure 9).

The present findings should not be extrapolated directly to other PDGF isoforms. PDGF-AA activates PDGFR-αα, whereas PDGF-AB activates PDGFR-αα and PDGFR-αβ; in contrast, PDGF-BB can activate PDGFR-αα, PDGFR-αβ, and PDGFR-ββ [46]. PDGF-AA and PDGF-AB may, therefore, share some PDGFR-α-mediated effects on fibroblast migration, proliferation, and extracellular matrix production, but they cannot be assumed to reproduce PDGFR-β-dependent functions relevant to pericyte recruitment and vascular stabilization [46,47]. Because PDGF-AA and PDGF-AB were outside the eligibility criteria and no included study directly compared these isoforms, the efficacy, safety, and dose findings of this review should be considered specific to rhPDGF-BB and becaplermin.

This mechanistic framework helps explain why macroscopic wound healing showed the clearest favorable pattern in the present review, whereas histological, extracellular-matrix, vascular, inflammatory, and molecular outcomes were less consistent. Wound closure is an integrated endpoint resulting from several overlapping processes and may improve even when individual mechanistic markers differ across models or assessment times. Conversely, activation of PDGFR-dependent signaling does not guarantee coordinated restoration of all phases of repair. Persistent inflammation, severe hypoxia, cellular senescence, neuropathy, infection, or extensive matrix destruction may continue to limit healing despite adequate exposure to rhPDGF-BB. The observed heterogeneity should, therefore, not be interpreted as evidence against biological activity, but it indicates that rhPDGF-BB functions as one component within a broader reparative network rather than as a universal molecular switch capable of independently correcting the diabetic wound environment (Figure 7, Figure 8 and Figure 9).

Local bioavailability represents a second determinant of therapeutic activity. Chronic wound fluids can contain high concentrations of neutrophil elastase and matrix metalloproteinases capable of degrading endogenous and exogenously administered growth factors, including PDGF-BB [60]. Soluble rhPDGF-BB may also be lost through diffusion, wound exudate, dressing changes, or rapid clearance before sustained receptor activation can occur. Hydrogels, nanofibrous scaffolds, extracellular-matrix-binding constructs, and other engineered delivery systems may improve therapeutic performance by protecting the growth factor, increasing its residence time, concentrating it within the wound bed, and providing a matrix that supports cell migration and tissue organization [8,9,10,11,31,32,35,51,52,53,60]. This interpretation is consistent with the broader favorable pattern observed among formulation-level comparisons [8,9,16,26,27,28,29,32,35]. However, because several of these systems incorporated additional growth factors, bioactive materials, or other therapeutic components, their effects represent formulation-level activity and cannot be attributed exclusively to rhPDGF-BB (Figure 8 and Figure 9).

Figure 9 integrates these observations into three interconnected mechanistic levels: the inhibitory diabetic wound microenvironment; the central rhPDGF-BB–PDGFR signaling module; and the modifying role of delivery systems. The central pathway links receptor activation to fibroblast recruitment, granulation tissue formation, collagen and extracellular matrix remodeling, perivascular cell support, vascular maturation, re-epithelialization, and wound closure. In parallel, hyperglycemia, oxidative stress, unresolved inflammation, hypoxia, and protease activity oppose these reparative processes, while biomaterial-based delivery may increase local stability and exposure. The model distinguishes well-established biological interactions from context-dependent or indirect mechanisms and should, therefore, be interpreted as an evidence-informed conceptual synthesis rather than as proof that every pathway was demonstrated consistently across the included studies (Figure 9).

From a translational perspective, these findings suggest that the effectiveness of rhPDGF-BB is unlikely to depend solely on the administered dose. Its activity may also be influenced by wound perfusion, protease burden, inflammatory state, infection, tissue viability, receptor-responsive cell populations, and the ability of the formulation to maintain biologically active concentrations within the wound bed [5,56,58,60,61]. This provides a plausible explanation for the difference between strong mechanistic rationale and uncertain clinical effectiveness. The most appropriate translational interpretation is, therefore, that rhPDGF-BB may amplify selected reparative pathways when the wound environment remains sufficiently responsive, while advanced formulations may extend or strengthen this signal by improving local bioavailability. Neither the preclinical findings nor the proposed mechanistic model support replacing debridement, offloading, infection control, vascular assessment, or other components of comprehensive diabetic wound care.

### 3.8. Strengths and Limitations of This Review

This review has several strengths. The protocol was finalized and registered in the OSF, the search was conducted across multiple databases and supplemented by citation searching, and study selection, data extraction, and risk-of-bias assessment were performed using predefined procedures. All 29 eligible controlled in vivo studies were incorporated into the evidence synthesis [8,9,10,11,12,13,14,15,16,17,18,19,20,21,22,23,24,25,26,27,28,29,30,31,32,33,34,35,36]. The combination of quantitative meta-analysis, structured descriptive synthesis, SYRCLE risk-of-bias assessment, and GRADE evaluation enabled the evidence to be examined at complementary levels. A further strength was the application of the attribution-specific framework at the intervention–comparator contrast level. This framework distinguished comparisons capable of isolating the specific contribution of rhPDGF-BB from formulation-level comparisons involving engineered variants, delivery systems, or multicomponent interventions in which the independent contribution of the growth factor could not be determined [8,9,14,16,26,27,28,29,31,32,34,35]. This distinction reduced the risk of incorrectly attributing the complete effect of a delivery platform or combination treatment to rhPDGF-BB alone.

Several limitations should, nevertheless, be considered. The included studies were heterogeneous with respect to animal species and strain, diabetic model, sex and age, wound dimensions, splinting, formulation, dose, administration schedule, comparator, outcome definition, and assessment time point [8,9,10,11,12,13,14,15,16,17,18,19,20,21,22,23,24,25,26,27,28,29,30,31,32,33,34,35,36]. Consequently, only three studies could be combined in the primary meta-analysis of percentage wound closure at days 10–12 [9,13,29], and only two provided compatible data for time to complete closure [24,27]. These small evidence sets precluded reliable subgroup analyses, meta-regression, and formal evaluation of small-study effects. The absence of extractable numerical data in many reports also limited the number of outcomes that could be synthesized quantitatively.

Methodological reporting was frequently insufficient. None of the studies reported a fully reproducible random sequence generation method; 28 were, consequently, judged as unclear-risk in this domain [8,9,10,11,13,14,15,16,17,18,19,20,21,22,23,24,25,26,27,28,29,30,31,32,33,34,35,36], whereas one used a fixed positional allocation scheme and was judged as high-risk [12]. Allocation concealment and intervention period blinding were also seldom described, with explicit coded and blinded procedures reported only in a small subset of experiments [28,29]. High risk arising from incomplete outcome data was identified in six studies [9,11,16,19,31,35], and unit-of-analysis concerns involving multiple wounds or other non-independent observations were identified in six [10,11,12,31,35,36]. Although unclear judgments do not demonstrate that bias occurred, the limited reporting reduced confidence in the internal validity and reproducibility of the evidence.

The descriptive synthesis increased coverage of outcomes that could not be meta-analyzed, but effect direction classifications do not incorporate effect magnitude, precision, statistical significance, or methodological quality. Moreover, findings from engineered rhPDGF-BB variants, delivery systems, or multicomponent interventions without an appropriate matched formulation-minus-factor comparator could only be interpreted at the formulation level [8,9,14,16,26,27,28,29,31,32,34,35]. Safety reporting was also incomplete and heterogeneous; only a subset of studies provided an eligible in vivo safety or tolerability assessment [8,10,11,13,16,19,20,24,27,29,34,36], and the absence of a reported adverse event could not be interpreted as evidence of safety.

Finally, the quantitative evidence was derived exclusively from mouse models with experimentally created acute dorsal wounds [9,13,29]. These models do not fully reproduce the chronicity, neuropathy, ischemia, infection, repeated mechanical loading, and comorbidity profile of human diabetic foot ulcers. The very low GRADE certainty, therefore, reflects not only imprecision and risk-of-bias concerns but also substantial translational indirectness. Accordingly, the findings should be interpreted as preclinical signals supporting further investigation rather than as direct estimates of clinical efficacy or safety.

### 3.9. Implications for Future Research

Future investigations should move beyond proof-of-concept efficacy studies and adopt prospective designs that prioritize reproducibility, biological relevance, and attribution of effect. Random sequence generation, allocation concealment, blinded outcome assessment, a priori sample size calculation, prespecified exclusion criteria, and appropriate handling of multiple wounds within the same animal should become routine. Both sexes should be represented unless a biologically justified restriction is prespecified, and sufficiently powered studies should evaluate potential sex-by-treatment interactions. ARRIVE 2.0 and the wound-specific WRAHPS recommendations should be incorporated at the study design stage, with complete reporting of the species, strain, age, sex, diabetic phenotype, glycemic criteria, wound creation procedure, analgesia, dressings, attrition, and experimental unit [62,63].

Model selection should reflect the intended clinical inference. Findings should be replicated across complementary models of diabetes rather than treating any single rodent model as definitive, with careful characterization of diabetes duration and severity and, when relevant, the incorporation of clinically important modifiers such as advanced age, obesity, ischemia, or infection. For rodent excisional wounds intended to approximate human repair, rigid splinting should be used and its integrity documented because it reduces contraction and shifts closure toward granulation tissue formation and re-epithelialization [39]. Wound diameter, depth, anatomical location, depilation method, number of wounds per animal, wound coverage, treatment timing, follow-up duration, and outcome time points should also be standardized. Harmonized assessment should combine serial planimetry with blinded histomorphometry and prespecified measures of re-epithelialization, granulation tissue, collagen organization, vascular maturation, inflammation, and molecular target engagement.

Intervention-specific designs must distinguish the effect of rhPDGF-BB from that of the delivery vehicle or additional active components. Studies of hydrogels, scaffolds, engineered proteins, or multicomponent formulations should include matched carrier-only controls, dose-matched free rhPDGF-BB, the individual co-interventions when applicable, and the complete formulation. Factorial designs would be particularly informative for identifying interactions between rhPDGF-BB and other components. Dose–response and dosing frequency experiments should be accompanied by local pharmacokinetic and pharmacodynamic measurements, including release kinetics, tissue retention, protein degradation, systemic exposure, PDGFR activation, and downstream pathway engagement. These measurements would clarify whether formulation-associated benefits result from greater local exposure, prolonged signaling, protection from proteolysis, or a true biological interaction.

Safety should be prospectively defined as an outcome rather than inferred from the absence of reported adverse events. Future studies should assess local irritation, excessive granulation or fibrosis, wound infection, body weight, clinical condition, hematologic and biochemical parameters, systemic exposure, and histopathological changes in major organs. Repeated-dose studies with follow-up extending into the remodeling phase are also needed to evaluate scar quality, tissue architecture, recurrence, and delayed toxicity.

Promising interventions should subsequently be validated in higher-fidelity translational systems, including standardized porcine models of impaired healing and human ex vivo skin, preferably obtained from diabetic donors or experimentally conditioned to reproduce relevant features of impaired repair. The feasibility of porcine and human-skin-based platforms is supported by studies included in this review [12,21], while WRAHPS provides a framework for their standardized implementation and reporting [63]. Convergent efficacy, mechanistic target engagement, reproducible manufacturing, and an acceptable safety profile across complementary models would provide a stronger basis for clinical translation than favorable wound closure findings from a single rodent experiment. Prospective protocol registration and availability of complete numerical data, including null and adverse findings, would further facilitate replication and future quantitative synthesis.

## 4. Materials and Methods

### 4.1. Protocol Registration and Reporting

This systematic review and meta-analysis was conducted and reported in accordance with the Preferred Reporting Items for Systematic Reviews and Meta-Analyses (PRISMA) 2020 Statement [64]. The finalized review protocol was registered on the Open Science Framework (OSF; registration: https://doi.org/10.17605/OSF.IO/T7X5D). The eligibility criteria, search strategy, study selection process, data extraction procedures, risk-of-bias assessment, and statistical analysis plan were predefined before the corresponding stages of this review were conducted. This review evaluated the preclinical efficacy of recombinant human platelet-derived growth factor-BB (rhPDGF-BB) in experimental diabetic wound healing using evidence from in vivo animal studies.

### 4.2. Eligibility Criteria

The eligibility criteria were established according to a single Population, Intervention, Comparator, Outcomes, and Study design (PICOS) framework. The primary review question was as follows: in non-human animals with experimental diabetic cutaneous wounds, what is the effect of exogenously administered, biologically active rhPDGF-BB on wound healing and related biological outcomes, and to what extent does the intervention–comparator design permit the observed effect to be attributed specifically to the growth factor? The intervention component encompassed the eligible delivery contexts described below because rhPDGF-BB remained the index biologically active intervention. The D/F framework was applied at the comparison level to determine the permitted level of attribution: findings were classified as directly attributable when an appropriate no-factor or otherwise equivalent comparator was available, and as formulation-level when the independent contribution of rhPDGF-BB could not be isolated. This classification did not represent a second review question or a separate PICOS framework.

Population (P): Eligible studies used non-human animals subjected to a cutaneous wound model in the presence of genetically determined, chemically induced, diet-associated, or otherwise experimentally established diabetes, or persistent hyperglycemia classified as diabetic according to the criteria used in the original study. No restrictions were imposed regarding animal species, strain, sex, age, method of diabetes induction, anatomical wound location, or type or dimensions of the cutaneous wound. The diabetic condition, cutaneous wound, and administration of the eligible intervention were required to coexist within the same experimental comparison. Studies containing both diabetic and normoglycemic groups were eligible. Normoglycemic groups could be recorded to characterize the experimental design and provide contextual references, but only comparisons involving diabetic animals were used to evaluate treatment efficacy. Experiments conducted exclusively in normoglycemic animals or involving non-cutaneous tissue repair were excluded.

Intervention (I): Eligible interventions comprised exogenously administered, biologically active recombinant human platelet-derived growth factor-BB (rhPDGF-BB), including becaplermin and the commercial becaplermin formulation Regranex. rhPDGF-BB could be evaluated as the index intervention, a component of a multicomponent therapy, or an active reference comparator. It could be administered as an unformulated growth factor, a commercial formulation, an engineered construct containing an intact and biologically active human PDGF-BB moiety, or as part of a biomaterial or drug delivery system. Accordingly, hydrogels, scaffolds, nanofibers, nanoparticles, surface-functionalized constructs, sequential delivery platforms, and other multicomponent formulations containing an identifiable rhPDGF-BB component were eligible, irrespective of dose, route, frequency, or treatment duration.

Studies were excluded when they did not administer an eligible exogenous rhPDGF-BB intervention and instead evaluated only endogenous PDGF expression; platelet-rich plasma, platelet lysates, or other platelet-derived preparations; PDGF-AA or PDGF-AB; PDGF-BB of non-human origin; unspecified PDGF products whose recombinant human identity could not be verified; PDGF-derived fragments or mimetics; or gene- or cell-based therapies intended to induce PDGF-BB expression without administering the recombinant human growth factor.

Comparator (C): Eligible comparators included untreated diabetic wounds, placebo or vehicle, standard wound care, unloaded or formulation-matched delivery systems, alternative rhPDGF-BB doses or formulations, and other active therapies. Studies in which rhPDGF-BB, becaplermin, or Regranex served as an active reference comparator were also eligible. For multicomponent interventions, comparisons with an otherwise equivalent formulation lacking rhPDGF-BB were considered informative of the specific contribution of the growth factor. When a formulation-matched comparator was unavailable, the study remained eligible, but its findings were attributed to the complete therapeutic formulation and were not interpreted as isolating the independent effect of rhPDGF-BB.

Outcomes (O): Studies were required to report at least one in vivo outcome related to diabetic cutaneous wound healing or its underlying biological processes. The primary outcome domain was macroscopic wound healing, assessed principally as percentage wound closure, change in wound area, residual wound area, or time to complete wound closure. Secondary eligible outcomes included re-epithelialization, granulation tissue formation, collagen deposition, extracellular matrix remodeling, angiogenesis, tissue perfusion, inflammatory or immune responses, cellular proliferation, molecular signaling, and treatment-related adverse effects.

Study design (S): Original controlled in vivo experimental studies using animal models of diabetic cutaneous wound healing were eligible. Reports combining in vivo and in vitro experiments were included only when they contained at least one eligible controlled animal experiment; only data obtained from the eligible in vivo component were extracted and synthesized. Exclusively in vitro or ex vivo studies, human clinical studies, uncontrolled experiments, reviews, systematic reviews, protocols, editorials, conference abstracts without sufficient methodological and outcome information, and publications without original experimental data were excluded. No restrictions were imposed on publication year, language, or follow-up duration.

### 4.3. Information Sources and Search Strategy

A comprehensive literature search was conducted in five electronic databases: PubMed, Embase, Scopus, Web of Science Core Collection, and ScienceDirect. The initial searches were performed on 12 February 2026 and were comprehensively updated on 29 July 2026. Each database was searched from inception, or from the earliest available coverage, to the date of the final search update.

The database-specific search strategies combined controlled vocabulary, including Medical Subject Headings (MeSH; https://www.ncbi.nlm.nih.gov/mesh/, (accessed on 12 February 2026)) in PubMed and Emtree terms in Embase, with free-text terms representing three principal concepts: (1) recombinant human platelet-derived growth factor-BB, including becaplermin and Regranex; (2) diabetes and experimental models of diabetes; and (3) cutaneous wounds, ulcers, and wound healing. Search syntax, field codes, truncation operators, and proximity operators were adapted to the requirements of each database.

No animal study or preclinical study design filters were applied because incomplete or inconsistent indexing could have resulted in the omission of eligible animal experiments or reports combining in vivo and in vitro components. No restrictions were imposed regarding publication year or language.

The reference lists of all included articles and relevant reviews were manually screened to identify additional eligible studies. Forward citation searches were also conducted for the included articles. Records identified through these supplementary methods were subjected to the same eligibility assessment as those retrieved through the database searches. The complete original and updated search strategies, corresponding search dates, and number of records retrieved from each database are provided in Supplementary Table S5.

### 4.4. Screening and Eligibility Assessment

All records identified through the database searches were imported into Mendeley Manager (https://www.mendeley.com/) for reference management and subsequently exported to Rayyan (https://www.rayyan.ai/), where duplicate records were identified and removed. Any records identified through supplementary searching of reference lists or forward citations were subjected to the same selection process.

Study selection was performed independently by two reviewers according to the predefined eligibility criteria and comprised two sequential phases. In the first phase, the titles and abstracts of all unique records were screened to identify potentially eligible reports. Records considered irrelevant by both reviewers were excluded. In the second phase, the full texts of all reports considered potentially eligible by at least one reviewer were sought and independently assessed against the complete PICOS criteria. Reports for which the full text could not be obtained were recorded as not retrieved and were not subjected to the eligibility assessment. Reasons for exclusion at the full-text stage were documented for each report.

Disagreements arising during either phase were resolved through discussion between the two reviewers until consensus was reached. When consensus could not be achieved, a third reviewer was consulted to make the final decision. Multiple reports describing the same experimental study were linked and counted as a single study. When a secondary or duplicate report did not provide unique eligible data beyond that available in a complete primary report, it was excluded at the full-text stage; otherwise, all relevant information across linked reports was considered collectively.

The study selection process was documented using the PRISMA 2020 flow diagram, including the numbers of records identified, duplicate records removed, unique records screened, reports sought for retrieval, reports not retrieved, full-text reports assessed for eligibility, reports excluded with reasons, and studies ultimately included in the systematic review.

### 4.5. Data Extraction

Data extraction was performed independently by two reviewers using a standardized electronic form developed specifically for this review. Before formal extraction, the form was pilot tested on a subset of the included studies to assess the clarity, consistency, and completeness of the predefined data items. Disagreements were resolved through discussion and consensus; when a discrepancy persisted, a third reviewer was consulted for adjudication.

The extracted bibliographic and study-level information included first author, year of publication, country, study objective, and experimental design. Animal and disease model characteristics comprised species, strain, sex, age or body weight, diabetic status, method of diabetes induction or genetic model, criteria used to confirm diabetes or hyperglycemia, and duration of diabetes before wound creation. Wound model characteristics included anatomical location, wound type, number and dimensions, depth, splinting or other procedures used to control wound contraction, and follow-up duration. When a publication combined in vivo and in vitro experiments, only information derived from eligible controlled animal experiments was extracted.

Intervention characteristics included the identity and role of rhPDGF-BB, becaplermin, or Regranex as the index intervention, a component of a multicomponent therapeutic formulation, or an active reference comparator. Additional data included formulation components, delivery system, dose or concentration, route, frequency and duration of administration, treatment schedule, and assessment time points. Comparator information comprised untreated or standard-care controls, placebo or vehicle controls, unloaded or formulation-matched delivery systems, alternative rhPDGF-BB doses or formulations, and other active treatments. For multicomponent interventions, the availability of a formulation-matched comparator lacking rhPDGF-BB was recorded to determine whether the observed effect could be attributed specifically to rhPDGF-BB or only to the complete therapeutic formulation.

Extraction was structured at the study–experiment–arm level to preserve distinctions among separate experiments, treatment arms, assessment time points, and outcome evaluations reported within the same publication. For studies containing multiple in vivo experiments or treatment arms, measurement methods and outcomes were attributed only to the experiments and groups in which they were actually assessed. Findings from ancillary experiments that did not contain an eligible rhPDGF-BB comparison were identified separately and were not attributed to rhPDGF-BB. Multiple reports describing the same underlying study were linked and considered collectively, whereas distinct experiments within the same report remained separately identifiable.

The original reporting and analytical structure used by the study authors was retained. Accordingly, sample sizes and units were recorded as animals, wounds, terminal cohorts, experimental replicates, or repeated experiments, as applicable. Unique-animal totals were not inferred when they could not be reliably established from the original report, and multiple wounds or observations from the same animal were not automatically interpreted as independent experimental units.

Outcome data were extracted according to the domains prespecified in the PICOS framework. These included macroscopic wound-healing outcomes—such as percentage wound closure, change or residual wound area, and time to complete closure—as well as re-epithelialization, granulation tissue formation, collagen deposition, extracellular matrix remodeling, angiogenesis, tissue perfusion, inflammatory or immune responses, cellular proliferation, molecular signaling, and treatment-related adverse effects. For each outcome, the reviewers recorded its definition, measurement method, reporting scale, assessment time point, experimental unit, intervention and comparator groups, group sample size, summary value, measure of dispersion, and principal findings.

### 4.6. Risk-of-Bias Evaluation Procedure

Risk of bias was assessed independently by two reviewers using the SYRCLE (Systematic Review Centre for Laboratory Animal Experimentation) Risk of Bias tool, which was specifically developed for intervention studies involving laboratory animals [65]. The assessment comprised ten domains: sequence generation, baseline characteristics, allocation concealment, random housing, blinding of caregivers and investigators, random outcome assessment, blinding of outcome assessors, incomplete outcome data, selective outcome reporting, and other potential sources of bias.

Each domain was judged as having a low, high, or unclear risk of bias in accordance with the SYRCLE signaling questions and guidance. Judgments were based on the information reported in the full-text articles and any available Supplementary Materials. An unclear risk-of-bias judgment was assigned when the reported information was insufficient to determine whether the methods adequately protected against the corresponding source of bias; insufficient reporting alone was not interpreted as evidence of high risk.

For reports combining in vivo and in vitro experiments, the assessment was restricted to the eligible controlled animal component. Methodological information pertaining exclusively to in vitro experiments or to ineligible experimental comparisons was not considered when assigning the risk-of-bias judgments.

Disagreements between the two reviewers were resolved through discussion and consensus. When consensus could not be reached, a third reviewer was consulted for adjudication. Judgments were recorded separately for each study and domain. No numerical summary score or overall global risk-of-bias judgment was assigned to preserve the domain-specific interpretation of the SYRCLE assessment.

### 4.7. Quantitative Synthesis and Statistical Analysis

Quantitative synthesis was conducted separately for each outcome. A study was included in an outcome-specific meta-analysis when it reported an interpretable rhPDGF-BB intervention–comparator contrast, assessed a sufficiently comparable outcome within a compatible follow-up window, and provided the numerical information required to calculate an effect estimate and its variance. For continuous outcomes, group means, measures of dispersion, and sample sizes were required; for dichotomous outcomes, the numbers of events and total experimental units in each group were required. No missing measures of variability were imputed. A meta-analysis was conducted only when at least two independent studies were considered sufficiently comparable in terms of outcome definition, intervention–comparator contrast, measurement scale, and assessment time. Studies that met the systematic review eligibility criteria but provided incomplete, non-extractable, or methodologically incompatible quantitative data were retained in the structured descriptive synthesis described in Section 4.8.

Primary quantitative analyses were restricted to comparisons permitting estimation of the specific effect of rhPDGF-BB. These comprised rhPDGF-BB, becaplermin, or Regranex administered alone versus an untreated, placebo, vehicle, standard-care, or otherwise appropriate control, as well as multicomponent rhPDGF-BB-containing formulations compared with the corresponding formulation lacking rhPDGF-BB. Formulation-level comparisons without a formulation-matched comparator, head-to-head comparisons with other active therapies, and comparisons between alternative rhPDGF-BB doses or formulations were eligible for separate quantitative synthesis only when at least two sufficiently compatible independent studies were available; otherwise, they were retained in the structured descriptive synthesis.

Outcomes were pooled only when their definitions, measurement approaches, reporting scales, comparator types, and assessment times were considered sufficiently compatible. For percentage wound closure, assessments conducted on days 10–12 were treated as a narrow common follow-up window. This window was defined before quantitative pooling because it was the narrowest consecutive interval containing numerically extractable and scale-compatible estimates from at least two independent studies, thereby limiting temporal variation while preserving the minimum evidence required for meta-analysis. The window was not intended to imply that days 10, 11, and 12 represented identical biological stages across diabetic or wound models. Assessments outside this interval were not combined. An exact-day sensitivity analysis was considered feasible only if at least two independent studies provided extractable means, measures of dispersion, and sample sizes at the same assessment time; no individual day met this requirement. When a publication contributed multiple independent eligible experiments to the same outcome and follow-up window, experiment-level estimates were first combined using fixed-effect inverse-variance pooling to obtain a single study-level estimate before inclusion in the overall random-effects meta-analysis. When sufficiently similar intervention groups shared a common comparator, the intervention groups were combined using standard formulas to avoid counting the comparator more than once.

Before pooling, the experimental unit and analytical independence were verified for each contributing comparison. Sample sizes were defined at the level of the independent animal rather than the individual wound. When multiple wounds were created in the same animal, a comparison was included in the quantitative synthesis only if the original analysis reduced the wound-level measurements to a single animal-level value or otherwise accounted for the within-animal design. Repeated measurements from the same animals were not entered as independent observations; only one estimate from the prespecified follow-up window or one reported time-to-closure summary per study was used. When within-animal correlation could not be reconstructed, and independence was not supported by the original report, the comparison was not pooled. Treating such observations as independent would underestimate the standard error and overweight the corresponding study. No within-animal correlation coefficient was imputed.

Macroscopic wound-healing outcomes were harmonized so that their direction was consistent across studies. When residual wound area was reported as a percentage of the initial wound area, it was converted to percentage wound closure using the following transformation:

Wound closure (%) = 100 − Residual wound area (%)(1)

Because this linear transformation has a coefficient with an absolute value of one, the corresponding standard deviation or standard error remained unchanged. Residual wound area was, therefore, not analyzed as a separate outcome because it represents the inverse expression of percentage wound closure.

For the outcomes meeting the pooling criteria, effects were expressed as mean differences (MDs) with 95% confidence intervals (CIs). Positive MDs indicated greater percentage wound closure and, therefore, favored rhPDGF-BB, whereas negative MDs for time to complete wound closure indicated faster healing and favored rhPDGF-BB. No outcome met the criteria for quantitative synthesis using standardized mean differences or risk ratios.

Because biological and methodological heterogeneity was anticipated across animal species, diabetic models, wound models, rhPDGF-BB formulations, delivery strategies, doses, and follow-up periods, pooled estimates were calculated using inverse-variance random-effects models. This model was selected a priori because the inferential target was the mean of a distribution of potentially different treatment effects across non-identical experimental settings, rather than a single common effect shared by all studies. A fixed-effect model was not considered appropriate because its common-effect assumption was not biologically or methodologically plausible for the included evidence. The small number of contributing studies increases the uncertainty of a random-effects estimate but does not make the common-effect assumption more defensible. Between-study variance (τ^2^) was estimated using restricted maximum likelihood, and Hartung–Knapp inference was applied to incorporate uncertainty in the pooled-effect estimate when only a small number of studies were available. Statistical heterogeneity was assessed using Cochran’s Q test and quantified using τ^2^ and I^2^. Prediction intervals were not reported because only two or three studies contributed to each meta-analysis, precluding meaningful estimation. Statistical tests were two-sided, with *p* < 0.05 considered statistically significant, although interpretation was based primarily on the magnitude, direction, and precision of the effect estimates.

For the primary meta-analysis of percentage wound closure at days 10–12, all eligible component experiments reported by Greenhalgh et al. were combined within the study before inclusion in the overall model. A sensitivity analysis restricted the contribution of Greenhalgh et al. to experiments evaluating rhPDGF-BB at 10 µg/day for 5 days; these experiments were again combined within the study before inclusion in the overall model.

Post hoc robustness analyses were performed for the primary meta-analysis. Leave-one-study-out analyses sequentially omitted each of the three study-level estimates and refitted the REML random-effects model. For these refits, the Hartung–Knapp method was used with the ad hoc standard error safeguard (‘adhoc.hakn.ci = “se”’), which prevents the Hartung–Knapp standard error from becoming smaller than the standard error from the conventional random-effects model. Influence diagnostics were calculated from the underlying REML model and included externally studentized residuals, DFFITS, Cook’s distances, covariance ratios, leave-one-out τ^2^ and Q statistics, hat values, study weights, and DFBETAS. Default diagnostic cutoffs were treated only as screening indicators, and the results were interpreted descriptively because the model contained only three studies. Additional sensitivity analyses were performed, where applicable, after excluding study-level estimates from (i) studies judged as having high risk of bias for incomplete outcome data, (ii) non-splinted wound models, (iii) comparisons classified as formulation-level (F), and (iv) studies with unresolved experimental-unit concerns. Meta-analysis was undertaken only when at least two independent studies remained; otherwise, the unpooled effect estimate and its 95% confidence interval from the remaining study were reported. No comparison classified as F and no study with unresolved experimental units contributed to the primary model; therefore, these two exclusions did not require model refitting.

Potential small-study effects were to be assessed using funnel plots and Egger’s regression test only when an outcome-specific meta-analysis contained at least ten studies. Because no meta-analysis reached this threshold, funnel plot asymmetry and small-study effects were not formally assessed. Analyses were conducted in R (version 4.5.2) using the meta package (version 8.5-0) and the metafor package (version 5.0-1).

### 4.8. Procedures for Synthesizing Outcomes Not Amenable to Meta-Analysis

Macroscopic, histological, mechanistic, safety, and other outcomes that did not meet the criteria for quantitative pooling were synthesized using a modified effect direction plot based on the approach described by Boon and Thomson [66]. The synthesis was reported in accordance with the Synthesis Without Meta-Analysis (SWiM) reporting guideline [67]. The specific study estimates included in the meta-analyses were not duplicated in the effect direction plot; however, other eligible findings from the same studies that were not quantitatively pooled remained eligible for the structured descriptive synthesis. Only findings derived from eligible controlled in vivo animal comparisons were included.

Studies were displayed in rows, and the following prespecified outcome domains were displayed in columns: macroscopic wound healing; histological repair; collagen and extracellular matrix remodeling; angiogenesis and tissue perfusion; inflammatory and immune responses; cellular proliferation and molecular signaling; and treatment-related adverse effects. All comparisons were oriented so that a favorable direction represented a better outcome in the rhPDGF-BB, becaplermin, Regranex, or rhPDGF-BB-containing formulation group relative to the corresponding comparator.

Each eligible finding was classified as favorable to the intervention, unfavorable to the intervention, or having no discernible direction. Classification was based on the observed direction of the effect and did not depend on statistical significance. Accordingly, a non-statistically significant finding was not classified as showing no effect when an interpretable effect direction was available. Findings reported only as showing no statistically significant difference, without an extractable point estimate or otherwise interpretable between-group direction, were classified as having no discernible direction. Mechanistic findings for which an increase or decrease could not be interpreted unambiguously as favorable or unfavorable to wound healing were also classified as having no discernible direction.

For a study–domain combination represented by a single eligible finding, the domain classification reflected the direction of that finding. When a domain was represented by multiple outcomes, experimental comparisons, conditions, or assessment time points within the same study, it was classified as favorable or unfavorable when at least 70% of the eligible findings showed the same direction. When this threshold was not reached, or when findings varied meaningfully across experimental conditions, doses, or assessment time points, the study–domain combination was classified as having no clear or consistent direction. Study–domain combinations without relevant eligible findings were classified as providing no eligible evidence. For the across-study domain summaries, the 70% rule was used solely as an exploratory descriptive predominance threshold. It was not an inferential test of consistency, and exceeding this threshold was not interpreted as evidence of a statistically robust or certain treatment effect.

Each eligible finding was additionally coded as direct evidence (D) when the comparison permitted attribution of the observed effect specifically to rhPDGF-BB, becaplermin, or Regranex. This category included comparisons of the growth factor or its commercial formulation with an appropriate comparator and comparisons of an rhPDGF-BB-containing formulation with an otherwise equivalent formulation lacking rhPDGF-BB. Findings derived from multicomponent rhPDGF-BB-containing formulations without a formulation-matched comparator were coded as formulation-level evidence (F). A study–domain combination containing both types of findings was designated D/F. Direct and formulation-level findings were evaluated separately when they produced discordant directions within the same study–domain combination. The number of eligible findings contributing to each classification was also recorded.

The modified effect direction plot was intended exclusively as a structured descriptive synthesis. It did not represent effect magnitude, precision, statistical significance, sample size, risk of bias, or certainty of evidence. No sign test or other inferential analysis of effect direction was performed.

### 4.9. Assessment of the Certainty of Evidence

The certainty of the evidence for the primary quantitative outcome—percentage wound closure at days 10–12—was assessed at the body-of-evidence level using the GRADE framework adapted for preclinical animal intervention studies proposed by Hooijmans et al. [68]. The assessment was restricted to the studies and comparisons contributing to the primary meta-analysis. The sensitivity analysis was considered when evaluating the robustness of the primary finding but was not graded as a separate body of evidence. Time to complete wound closure, outcomes included in the structured descriptive synthesis, and findings reported only descriptively were not assigned separate certainty ratings because the certainty assessment was restricted to the primary quantitative outcome.

In accordance with the proposed preclinical GRADE framework, evidence derived from randomized animal experiments was initially considered high-certainty evidence. Inadequate, absent, or unreported randomization and other methodological limitations were subsequently considered within the risk-of-bias domain. Certainty was assessed across five downgrading domains: risk of bias, inconsistency, imprecision, indirectness, and publication bias. The body of evidence could be downgraded by one level for serious concerns or by two levels for very serious concerns within a domain.

Risk-of-bias judgments were informed by the outcome-specific SYRCLE assessments of the studies contributing to the primary meta-analysis, while considering the relative contribution of each study to the pooled estimate. Inconsistency was evaluated by examining the direction and magnitude of the individual effect estimates, overlap of confidence intervals, statistical heterogeneity quantified using I^2^ and τ^2^, and plausible methodological or biological explanations for between-study differences.

Imprecision was assessed according to the number of contributing studies and animals, the width of the confidence interval around the pooled estimate, and whether the interval included the null effect and encompassed materially different conclusions. Because no validated minimally important difference was established for percentage wound closure across the included animal models, judgments focused primarily on whether the confidence interval was compatible with potentially important benefit, little or no benefit, or an effect in the opposite direction.

Indirectness was evaluated in relation to both the prespecified preclinical PICOS and the translational relevance of the evidence to human diabetic foot ulcers. The assessment considered animal species and strains, mechanisms and duration of diabetes, wound characteristics, correspondence of the experimental wounds to chronic human diabetic foot ulcers, intervention formulation, dose and route of administration, comparator, outcome measurement, and follow-up period. The same underlying limitation was not used to downgrade certainty more than once.

Publication bias was assessed qualitatively by considering the comprehensiveness of the search, the possible non-publication or selective availability of studies, and patterns such as a concentration of small studies with favorable findings. Within-study selective outcome reporting was considered under the risk-of-bias domain and was not counted again as publication bias. Funnel plots and statistical tests for small-study effects were not used because the number of studies contributing to the primary meta-analysis was insufficient for reliable evaluation.

Potential upgrading factors—including a large magnitude of effect, a dose–response relationship, and circumstances in which all plausible residual confounding would reduce the observed effect—were considered when applicable. Consistency across independent animal species or experimental models was also examined as a potential preclinical upgrading factor. Upgrading factors were considered only after any downgrading decisions and could not increase certainty beyond high. The same evidence was not counted simultaneously as an upgrading factor and as part of the assessments of inconsistency or indirectness.

Two reviewers independently assessed each domain and assigned the overall certainty rating. Disagreements were resolved through discussion and consensus, with consultation of a third reviewer when necessary. Overall certainty was classified as high, moderate, low, or very low. The final rating and the reasons for each downgrading or upgrading decision were reported in a single-outcome Summary of Findings table.

## 5. Conclusions

Across 29 controlled in vivo studies, directly attributable comparisons showed a favorable direction for macroscopic wound healing in 17 of 22 studies, although this descriptive pattern should be regarded as exploratory. Both quantitative syntheses were restricted to directly attributable contrasts but remained inconclusive. The primary meta-analysis suggested a 12.94-percentage-point increase in wound closure at days 10–12; however, the confidence interval crossed the null, and the certainty of evidence was rated as very low. The analysis of time to complete closure similarly favored rhPDGF-BB relative to factor-free carrier controls but was based on only two studies and was associated with substantial uncertainty. Directly attributable histological, extracellular matrix, vascular, inflammatory, and molecular findings were more heterogeneous.

Formulation-level comparisons involving engineered rhPDGF-BB variants, delivery systems, or multicomponent interventions showed broader favorable reparative patterns. These findings apply to the complete formulations and cannot establish the independent contribution of rhPDGF-BB when an appropriate matched formulation-minus-factor comparator was unavailable. Safety and tolerability were insufficiently and inconsistently assessed at both attribution levels, preventing conclusions regarding either safety or harm, particularly under repeated dosing or prolonged follow-up. Overall, the directly attributable evidence suggests possible biological activity of rhPDGF-BB in diabetic wound repair but does not establish a precise or robust treatment effect, while the formulation-level evidence identifies potentially promising therapeutic systems without demonstrating the isolated efficacy of the growth factor. Confirmation will require standardized, adequately powered, and bias-controlled studies incorporating matched comparators, pharmacokinetic and pharmacodynamic assessment, prospective safety evaluation, and validation in higher-fidelity translational models.

## Figures and Tables

**Table 1:** General characteristics of the included studies and animal models.

Study	Country	Experimental Design	Species and Strain	Sex	Age/Body Weight	Diabetes Model	Diabetes Confirmation and Duration	Reported Sample Size and Unit	Follow-Up
Fang et al., 2025 [8]	Taiwan	Controlled, three parallel groups; allocation method NR	Mouse, BALB/c	Male	8 weeks; 20–25 g	STZ-induced diabetes	STZ 40 mg/kg (route NR); fasting glucose > 300 mg/dL after several weeks	n = 3/group (9 mice across normal, untreated diabetic, and treated diabetic groups)	18 days
Deng et al., 2025 [9]	China	Randomized controlled, six parallel groups	Mouse, C57BL/6J	Male	6 weeks; 7-day acclimation; weight NR	STZ-induced diabetes	STZ 180 mg/kg i.p.; fasting glucose > 11.1 mmol/L at 72 h; interval from confirmation to wounding NR	n = 6/group; 36 mice total	22 days (wound closure assessed until day 12; healed tissue assessed on day 22)
Saklani et al., 2024 [20]	India	Controlled, seven groups in both normal and diabetic models; allocation method NR	Mouse, BALB/c	Male	4–6 weeks; 30–32 g	Alloxan-induced diabetes	Alloxan 400 mg/kg i.p.; blood glucose > 190–200 mg/dL; wounds created after 1 week	Seven groups × n = 4 in each normal and diabetic model (28/model); article states ‘28 pairs’	14 days
Ganesh et al., 2023 [30]	India	Randomized controlled, six parallel groups	Mouse, C57BL/6	Male	6–8 weeks; 20–25 g	STZ-induced diabetes	STZ 50 mg/kg i.p. for 5 consecutive days; fasting glucose > 200 mg/dL at 2 weeks	n = 5/group; 30 mice total (20 diabetic and 10 normoglycemic)	14 days
Alshoubaki et al., 2023 [31]	Australia/Japan	Controlled, three treatment groups; allocation method NR	Mouse, BKS.Cg-Dock7m +/+ Leprdb/J (Leprdb/db)	Male	12–14 weeks; weight NR	Genetic Leprdb/db model	Genetic model; glycemic confirmation and diabetes duration NR	8–10 wounds/group/time point; two wounds/mouse; exact unique mice NR	9 days
White et al., 2021 [32]	United States	Controlled ‘walk-in clinic’ design with spontaneous diabetes; random allocation reported	Mouse, NOD; NOR nondiabetic controls	Female	Variable; glucose surveillance began at 8 weeks; weight NR	Spontaneous type 1 diabetes	Two readings > 250 mg/dL or one >400 mg/dL; insulin-treated; glucose stabilized at 250–300 mg/dL for 2 consecutive weeks before wounding	n = 3–20 across groups/outcomes; exact unique mice NR	3 or 7 days
Nguyen et al., 2018 [33]	United States	Controlled parallel-group experiments, repeated at least twice; random allocation reported	Mouse, BKS.Cg-Dock7m +/+ Leprdb/J (db/db)	Female	8 weeks; approximately 40 g	Genetic Leprdb/db model	Genetic model; glycemic confirmation and diabetes duration NR	Becaplermin comparison: 34 mice at day 7 (vehicle 11; R-ND-336 11; becaplermin 12); n = 7–9/group at days 10/14 after scheduled harvests	14 days
Gallant-Behm et al., 2018 [34]	United States	Controlled parallel and combination experiments; allocation method NR	Mouse, db/db (strain designation otherwise NR)	Male	6–8 weeks; weight NR	Genetic db/db model	Genetic model; glycemic confirmation and diabetes duration NR	n = 8–10 mice/group per experiment; two wounds/mouse; exact unique mice across experiments NR	10 days
Ishihara et al., 2018 [35]	United States	Randomized controlled experiments; not blinded; wound-healing assay repeated three times	Mouse, C57BLKS/J-m/Leprdb (db/db)	Male	10–11 weeks; weight NR	Genetic Leprdb/db model	Genetic model; glycemic confirmation and diabetes duration NR	27 mice in the main wound-healing model; separate n = 10 cohorts for retention and flow cytometry; cohort overlap NR	10 days (flow cytometry at day 5)
McLaughlin et al., 2017 [36]	United States	Three independent controlled experiments; wound treatments randomized within animal	Rat, Sprague–Dawley	Male	6 weeks at induction; 170 ± 2 g initially; diabetic rats 324 ± 7 g at wounding	STZ-induced type 1 diabetes	STZ 40 mg/kg i.p. on 2 consecutive days; diabetes within 4–5 days; blood glucose > 350 mg/dL for 5 weeks before wounding	Approximately 15 hyperglycemic rats/experiment × 3 (approximately 45 diabetic rats); additional normoglycemic controls NR	14 days (mechanistic cohorts at days 1, 2, and 4)
Lee et al., 2016 [10]	Taiwan	Randomized controlled, three parallel groups	Rat, Sprague–Dawley	Male	12 weeks; 306 ± 17 g	STZ-induced diabetes	STZ 70 mg/kg i.p.; blood glucose > 300 mg/dL at 72 h; interval from confirmation to wounding NR	n = 6/group; 18 rats total	14 days
Lee et al., 2015 [11]	Taiwan	Randomized controlled, three parallel groups	Rat, Sprague–Dawley	NR	Age NR; 368 ± 42 g	STZ-induced diabetes	STZ 70 mg/kg i.p.; plasma glucose > 300 mg/dL at 72 h; wounding 1 week after STZ	n = 6/group; 18 rats total	14 days
O’Brien et al., 2014 [12]	United States	Controlled paired wound experiments within pigs; side assignment was fixed rather than randomized	Pig, Yorkshire	Female	2–3 months at arrival; 20–25 kg; ≥1-week acclimation	STZ-induced diabetes	STZ 150 mg/kg i.v.; fasting glucose > 200 mg/dL and maintained mainly at 250–450 mg/dL; becaplermin comparison at 45 days post-STZ	Six diabetic pigs induced; one died, and five remained; triplicate wounds/treatment/pig; pigs contributing to becaplermin comparison NR	21 days overall (becaplermin comparison assessed on days 7 and 14)
Park et al., 2014 [13]	United States	Randomized controlled, two parallel groups; histopathology masked	Mouse, BKS.Cg-m +/+ Leprdb (db/db)	Male	16 weeks; 52.6 ± 0.4 g	Genetic Leprdb/db model	Genetic model; study-specific glycemic threshold and diabetes duration NR	n = 12/group; 24 mice total	11 days
Allen et al., 2014 [14]	United States	Controlled, five parallel groups; allocation method NR	Mouse, C57BL/6J and Leprdb/db	NR	8–12 weeks; weight NR	Genetic Leprdb/db model	Genetic model; blood glucose assessed, but threshold and diabetes duration NR	n = 8/group; 40 mice total (32 diabetic and 8 wild-type)	28 days or until closure
Dwivedi and Chaudhary, 2012 [15]	India	Controlled, four parallel groups; treatment allocation method NR	Rat, Wistar albino	Male	Age NR; 150–200 g	Alloxan-induced diabetes	Alloxan 75 mg/kg i.p.; fasting glucose > 200 mg/dL at 72 h; later interval to wounding NR	n = 24 analyzed: 6 normoglycemic; 18 diabetic rats randomly selected from 22/24 alloxan-treated rats	14 days
Berdal et al., 2011 [16]	Norway	Open-label controlled, eight parallel groups	Mouse, C57Bl/KsBom-db/db and C57Bl/KsBom-db/+	NR	2–4 months; diabetic mean of 41.3 ± 0.7 g	Genetic db/db model	Only db/db mice with plasma glucose ≥ 16 mmol/L included; diabetes present at 2–4 months	87 mice total: 75 diabetic across seven groups and 12 nondiabetic; PDGF-BB + IGF-1 group n = 12	17 days for serial closure; biopsies on days 17–18; observation period of up to 45 days
Cheng et al., 2011 [17]	United States	Controlled three-group experiments, repeated independently; allocation method NR	Mouse, BKS.Cg-m +/+ Leprdb/J (db/db)	NR	6 weeks; weight NR	Genetic Leprdb/db model	Genetic model; no study-specific glycemic threshold reported	n = 3/group/experiment; placebo/F-5/becaplermin comparison quantified across 3 independent experiments (27 mice)	18 days for the head-to-head comparison
Tkalčević et al., 2007 [18]	Croatia	Controlled three-group terminal-cohort experiment; allocation method NR	Mouse, C57BL/KsJ db +/db+	Female	6 weeks; 38–45 g	Genetic db/db model	Stable hyperglycemia confirmed throughout; numeric threshold and diabetes duration NR	3 groups × n = 5/time point × 5 terminal times; 75 mice total	24 days (terminal cohorts on days 6, 9, 12, 18, and 24)
Cheng et al., 2007 [19]	China	Controlled, three parallel groups with terminal assessments; allocation method NR	Rat, Wistar	Male	Age NR; 160–190 g	Pre-existing experimental diabetes; induction method NR	Purchased as diabetic; mean blood glucose: 23 ± 3.54 mmol/L; duration before wounding NR	n = 6–8/group across three groups (18–24 enrolled); n = 5–7 analyzed per outcome/time	14 days
Zhao et al., 2006 [21]	United States	Controlled paired-wound experiment with contralateral vehicle control; allocation method NR	Mouse, BKS Cg-m +/+ Lepr(db)	NR	NR	Genetic Leprdb model	Genetic model; glycemic confirmation and diabetes duration NR	12 mice total, two groups of six; paired treatment and vehicle wounds within each mouse	11 days
Ševeljević-Jaran et al., 2006 [22]	Croatia	Controlled multi-arm experiments with separate imaging and terminal histology cohorts; macroscopic scoring observer-blinded	Rat, Wistar Han	Male	Age NR; 250–290 g	Alloxan-induced hyperglycemia	Alloxan 175 mg/kg s.c.; 5 days; included if glucose at 22.5–40.5 mmol/L	n = 6/group in each experiment; separate 6-day imaging and 3-/7-day histology cohorts; exact unique rats NR	8 days (imaging until day 6; histology on days 4 and 8)
Chan et al., 2006 [23]	United States	Controlled parallel experiments across wound sizes and vehicle controls; allocation method NR	Mouse, C57BL/6 wild-type and lep/r db/db	Male	6–8 weeks; weight NR	Genetic leptin-receptor db/db model	Hyperglycemic phenotype confirmed at harvest (db/db mean: 590 ± 6 mg/dL); no pre-wounding threshold/duration reported	n = 8/group; multiple wound-size and vehicle experiments; exact unique mice NR	Up to 24 days (serial photographs for 3 weeks; early histology time point unclear because it was reported as day 7 in the Methods and day 5 in the corresponding figure; late histology on day 24)
Uhl et al., 2003 [24]	Germany	Randomized controlled hyperglycemic arm with attrition; repeated-measures follow-up	Mouse, barrier-bred hairless strain	Both sexes	8–10 weeks; 22–28 g	STZ-induced hyperglycemia	STZ 60 mg/kg i.v.; 7 days before wounding; glucose > 300 mg/dL on wound day	24 hyperglycemic mice randomized; 17 analyzed (vehicle: 9; PDGF-BB: 8); 60 mice total across all model strata	24 days
Rodgers et al., 2003 [25]	United States	Controlled, four parallel groups in the diabetic wound model; wound assessment blinded	Mouse, C57BL/KsJ-db/db	Male	NR	Genetic db/db model	Genetic model; glycemic confirmation and diabetes duration NR	n = 5/group; 20 mice in the diabetic model	25 days
Brown et al., 1997 [26]	United States	Controlled two-arm terminal-cohort study; apoptosis scoring blinded; allocation method NR	Mouse, C57BL/KsJ-db/db and nondiabetic littermates	NR	8–12 weeks; weight NR	Genetic db/db model	Genetic model; study-specific glycemic threshold and duration NR	Natural-history arm n = 4/group/time at 8 times; treatment arm n = 6/group/time (four diabetic treatments, days 5 and 10); nondiabetic controls n = 6 as reported	28 days overall; treatment arm assessed on days 5 and 10
Kiritsy et al., 1995 [27]	United States	Two controlled parallel-group experiments; allocation method NR; wound assessment blinded	Mouse, C57BL/KsJ-db/db	NR	15 weeks; 40–50 g	Genetic db/db model	Random subsamples tested 1 week preoperatively; diabetic glucose means: 1109 ± 60 and 812 ± 79 mg/dL; no eligibility threshold	104 mice total: experiment 1, n = 31 + 31; experiment 2, n = 16 + 14 + 12	Until complete healing, up to 63 days; treatment until day 24
Brown et al., 1994 [28]	United States	Randomized, blinded controlled experiments repeated two to three times	Mouse, C57BL/KsJ-db/db	Female	8–12 weeks; 35–45 g	Genetic db/db model	Diabetic phenotype described; laboratory mean glucose > 900 mg/dL; no study-specific eligibility threshold	n = 8–10/group/experiment; experiments repeated 2–3 times; pooled PDGF-BB/TGF-α analysis n = 36/group; exact unique total NR	21 days
Greenhalgh et al., 1990 [29]	United States	Randomized, coded, blinded controlled experiments with multiple terminal cohorts	Mouse, C57BL/KsJ-db/db and heterozygous nondiabetic littermates	Both sexes	8–12 weeks; diabetic: 40–50 g, nondiabetic: 25–32 g	Genetic db/db model	Mean glucose: 927 ± 35 mg/dL in 52 diabetic mice; no study-specific eligibility threshold	Intervention experiments generally n = 4–10/group; natural-history series n = 5–19/time point; exact unique total NR	Up to 42 days in serial studies; one chronic wound observed to day 90

Note: Sample sizes and experimental units are reproduced as reported. When n refers to wounds, assessment-specific observations, experimental replicates, repeated experiments, or terminal cohorts rather than unique animals, this is stated explicitly. Unique-animal totals were not inferred when the source report was insufficient; detailed group-level denominators and units are provided in Table S1. db/db, leptin receptor-deficient diabetic model; i.p., intraperitoneal; i.v., intravenous; NOD, non-obese diabetic; NOR, non-obese resistant; NR, not reported; s.c., subcutaneous; STZ, streptozotocin.

**Table 2:** Wound models and experimental treatment protocols.

Study	Wound Model	Contraction Control	Role of rhPDGF-BB	Attrib. Code	Product/ Formulation	Delivery System	Dose/ Concentration	Administration Schedule	Comparator(s)	Assessment Time Points
Fang et al., 2025 [8]	Single 10 mm circular full-thickness dorsal wound	No splint; transparent dressing	Main component of experimental formulation	F	Human rhPDGF-BB in PEI–polypyrrole–Pluronic F127 thermosensitive hydrogel	Topical injectable hydrogel; transparent dressing	0.5 µg of rhPDGF-BB in 100 µL/wound	Every other day until day 10 (5 applications)	Untreated diabetic and nondiabetic wounds; transparent dressing in all groups	Photographs every other day; histology on day 18
Deng et al., 2025 [9]	Single 8 mm circular full-thickness dorsal wound	No splint; breathable gauze	Main intervention, alone and in dual-GF formulation	D/F	Transgenic silk-sericin hydrogels containing human PDGF-BB, EGF, or both	10 mm × 2 mm hydrogel disc or topical solution	PDGF-BB approximately 1.18 µg/g hydrogel; EGF approximately 1.63 µg/g	Single application at wounding; redosing NR	Saline; WT-sericin hydrogel; EGF-sericin; commercial EGF + PDGF-BB solution	Days 0, 3, 7, and 12; healed tissue on day 22
Saklani et al., 2024 [20]	Single 6 mm circular full-thickness dorsal wound	No splint	Active reference comparator	D	Becaplermin gel (standard treatment)	Topical gel	1 mg gel/wound/day; becaplermin concentration NR	Once daily for 4 days	Blank Na-CMC hydrogel and five peptide hydrogels; evaluated in normal and diabetic mice	Days 0, 2, 4, 7, 10, and 14; histology on days 4/7/14 in normal and 7/14 in diabetic mice
Ganesh et al., 2023 [30]	Four 6 mm circular full-thickness dorsal wounds/mouse	No splint; Transpore dressing	Active reference comparator	D	Regranex 0.01% becaplermin gel	Topical gel	0.01%; amount/application NR	Once daily for 14 days	Diabetic vehicle; pterostilbene 5 or 10 mg/kg i.p.; normoglycemic vehicle or pterostilbene	Days 0, 7, 10, and 14; terminal tissue on day 14
Alshoubaki et al., 2023 [31]	Two 5 mm circular full-thickness dorsal wounds/mouse	No splint; Adaptic and secondary bandages	Main intervention and engineered variant	D/F	Wild-type human rhPDGF-BB and AREG26-38-PDGF-BB fusion	Topical solution beneath dressing	0.5 µg of protein in 10 µL/wound	Single application on the day after wounding	Saline; wild-type rhPDGF-BB	Day 9
White et al., 2021 [32]	Two 6 mm circular full-thickness dorsal wounds/mouse	Silicone rubber splints	Main intervention, alone and in multi-GF combinations	D/F	Wild-type or PlGF-2-fused human PDGF-BB, with VEGF-A165 and/or HB-EGF, in fibrin	Topical fibrin gel under clear dressing	200 ng of each growth factor/wound	Single administration at wounding	Fibrin control; wild-type and engineered single-GF and multi-GF regimens	Days 3 and 7
Nguyen et al., 2018 [33]	Single 8 mm circular full-thickness dorsal thoracic wound	Splinted model stated; splint method NR	Active reference comparator	D	Regranex 0.01% becaplermin gel	Topical gel	5 µg of becaplermin in 50 µL/wound/day	Once daily from day 1 to day 14	Vehicle and MMP-9 inhibitor R-ND-336 (50 µg/wound/day)	Days 0, 7, 10, and 14; molecular analyses on day 7
Gallant-Behm et al., 2018 [34]	Two 6 mm circular full-thickness dorsal wounds/mouse	Tegaderm dressing; no rigid splint	Main intervention, alone and combined with miRNA inhibitors	D/F	Human rhPDGF-BB in PBS, alone or combined with MRG-110	Four intradermal injections around each wound	2 µg of rhPDGF-BB/wound; MRG-110 10–100 nmol/wound in dose-ranging experiments and 30 or 92 nmol/wound in the combination experiment	Days 0, 2, 4, and 8	PBS; rhVEGF165; dose-ranging miR-92a inhibitors; MRG-110 monotherapy; rhPDGF-BB monotherapy	Day 10; distribution 48 h after final dose
Ishihara et al., 2018 [35]	Four 6 mm circular full-thickness dorsal wounds/mouse	No splint; adhesive film	Component of dual-GF formulation	F	Human rhPDGF-BB + rhVEGF-A165 in fibrin, with or without laminin peptide	Topical fibrin gel	50 ng of PDGF-BB + 100 ng of VEGF-A165 in 80 µL/wound	Single application at wounding	Fibrin alone; laminin-peptide fibrin; GF-loaded fibrin without peptide	Days 4, 7, and 10; flow cytometry on day 5
McLaughlin et al., 2017 [36]	Four 6 mm circular full-thickness dorsal wounds/rat to panniculus	No splint; wounds left undressed	Active reference comparator	D	Regranex becaplermin gel	Topical gel	0.05 mL/wound/day; active concentration NR	Once daily to day 14	Vehicle/saline cream and naltrexone 0.03% cream; wound-level randomization	Every other day until day 14; mechanistic cohorts on days 1, 2, and 4
Lee et al., 2016 [10]	Two 8 mm circular full-thickness dorsal wounds/rat	No splint	Main component of experimental scaffold	D	rhPDGF-BB-eluting PLGA–collagen scaffold	Implanted nanofibrous scaffold	Fabrication mixture: 5 mg of rhPDGF-BB with 280 mg of PLGA + 140 mg of collagen; per-wound dose NR	Single scaffold placement at wounding; no replacement	Matched PLGA–collagen scaffold and PLGA-only scaffold	Days 3, 7, and 14
Lee et al., 2015 [11]	Two 8 mm circular full-thickness dorsal wounds/rat	No splint	Main component of experimental membrane	D	rhPDGF-BB-loaded PLGA nanofibrous membrane	Topical nanofibrous membrane	Fabrication mixture: 5 mg of rhPDGF-BB with 280 mg of PLGA; per-wound dose NR	Single membrane placement at wounding; no replacement	PLGA-only membrane and conventional gauze	Days 0, 3, 7, and 14
O’Brien et al., 2014 [12]	Multiple 1.5 × 1.5 cm full-thickness dorsal wounds, approximately 5 mm deep, in paired fields	No splint; Opsite/gauze/wrap	Active reference comparator	D	Becaplermin gel (Regranex)	Topical gel to triplicate wounds	As prescribed; exact dose/application NR	Single application at wounding	5% CMC vehicle and Hsp90α F-5 peptide (45 µM)	Days 0, 7, 14, and 21; becaplermin comparison on days 7 and 14; histology on day 14
Park et al., 2014 [13]	Two 8 mm circular full-thickness dorsal wounds/mouse	Silicone donut splints	Main intervention	D	Purified human rhPDGF-BB in 5% PEG/PBS	Topical solution under coverslip/Tegaderm	3 µg in 30 µL/wound/day (0.01%)	Immediately after surgery and once daily for 10 days	5% PEG/PBS vehicle	Days 0, 2, 4, 6, 8, 10, and 11; terminal on day 11
Allen et al., 2014 [14]	Two 6 mm circular full-thickness dorsal wounds/mouse	Silicone stents	Main intervention, alone and combined with AMD3100	D/F	Regranex 0.01% becaplermin gel	Topical gel; AMD3100 administered i.p.	2 µg of becaplermin/wound/day; AMD3100 10 mg/kg/day	Started on day 3 and continued until closure	Wild-type saline; diabetic saline; AMD3100 alone; rhPDGF-BB alone	Wound imaging every 7 days; blood on days 0, 7, 14, and 21; histology on day 28
Dwivedi and Chaudhary, 2012 [15]	Single circular 500 mm^2^ full-thickness wound, 0.2 cm deep	No splint	Active reference comparator	D	Becaplermin gel obtained from a pharmacy	Topical gel after saline cleansing	50 mg of gel/wound/application; becaplermin concentration NR	Twice daily for 14 days	Untreated diabetic; Ampucare at 0.5 mL twice daily; nondiabetic control	Days 0, 5, 10, and 14; terminal tissue on day 14
Berdal et al., 2011 [16]	Single 1.5 × 1.5 cm full-thickness dorsal wound through panniculus	No splint; OpSite dressing	Component of two-GF active control	F	Human rhPDGF-BB + rhIGF-1 in human serum albumin/saline	Topical solution under OpSite	1 µg of rhPDGF-BB + 1 µg of rhIGF-1 in 100 µL/wound	Once daily on days 0–4	Four aminated β-1,3-D-glucan schedules; diabetic placebo ± insulin; nondiabetic placebo	Seven measurements over 17 days; growth factor biopsies on days 17–18; observation until day 45
Cheng et al., 2011 [17]	Single 1.2 × 1.2 cm full-thickness dorsal wound	No splint; bandage/Coban	Active reference comparator	D	Regranex (becaplermin 0.01%; 100 µg/g PDGF-BB)	Topical gel	100 µL of gel/wound; active mass per wound NR	Single application at wounding	CMC placebo and Hsp90α F-5 peptide (~1 mM) in CMC; CMC reported as 5% in Methods and 10% in Figures 5 and 6 of the original study	Days 0, 5, 10, 14, and 18; histology on day 14
Tkalčević et al., 2007 [18]	Single 8 mm circular full-thickness non-occluded interscapular wound	No splint; non-occluded	Main intervention	D	*E. coli*-derived human rhPDGF-BB in PBS/0.1% mouse serum albumin	Topical solution	1 µg in 50 µL/wound/day	Immediately after wounding and once daily until day 18	Vehicle and PL14736, a synthetic 15-amino-acid peptide (100 µg/wound/day)	Terminal cohorts on days 6, 9, 12, 18, and 24
Cheng et al., 2007 [19]	Four circular full-thickness dorsal wounds/rat, 2.54 cm^2^ and 4–5 mm deep	No splint	Main intervention	D	Regranex 0.01% rhPDGF-BB in 3.5% CMC	Topical gel	7.0 µg of rhPDGF-BB/cm^2^	Once daily on days 1–14	Untreated and 3.5% CMC vehicle	Days 3, 7, and 14
Zhao et al., 2006 [21]	Two 8 mm circular full-thickness dorsal wounds/mouse	No splint reported	Active reference comparator	D	Becaplermin gel (Regranex)	Topical gel; paired contralateral wounds	10 mg of becaplermin formulation/wound/application, as reported; active rhPDGF-BB mass NR	Day 0 and every second day until day 10 (6 applications)	Contralateral 2% CMC; separate SBD.4A/CMC paired group	Day 11
Ševeljević-Jaran et al., 2006 [22]	Single 8 mm circular full-thickness dorsal wound to fascia	No splint; no dressing	Active reference comparator	D	Regranex 0.01% becaplermin gel	Topical gel	20 µg of becaplermin in 0.2 g of gel/wound/day	Once daily for 5 days; separate 3- and 7-day histology cohorts	Untreated; Carbopol vehicle; PL14736 10–1000 µg/wound	Days 0, 1, 4, and 6; terminal tissue on days 4 and 8
Chan et al., 2006 [23]	Single circular full-thickness dorsal wound; related experiments used 0.6, 1.0, or 1.5 cm^2^ wounds	No splint; Opsite dressing	Main intervention	D	Regranex containing rhPDGF-BB in a 5% PEG vehicle	Topical solution injected beneath Opsite	10 µg of rhPDGF-BB in 0.2 mL/wound/day	Once daily on days 1–5	Diabetic vehicle controls receiving saline or 5% PEG; untreated wild-type control	Photographs every 2–3 days for 3 weeks; early histology time point unclear (day 7 in the Methods and day 5 in the corresponding figure); late histology on day 24
Uhl et al., 2003 [24]	Single 2.5 mm circular full-thickness ear wound to cartilage	Anatomic ear model; semipermeable membrane	Main intervention	D	Amgen human rhPDGF-BB in sodium acetate/0.25% albumin	Topical solution under membrane	3 µg in 3 µL/wound	Single application at wounding	Carrier vehicle	Baseline and days 1, 3, 6, 9, 12, 18, and 24; followed to closure
Rodgers et al., 2003 [25]	Single 13 mm circular full-thickness dorsal wound	No splint; semipermeable Tegaderm dressing	Active reference comparator	D	Regranex formulation of recombinant human PDGF	Topical gel under dressing	100 µL/wound/day; active concentration NR	Once daily on days 0–4	Placebo; angiotensin-(1–7); NorLeu^3^-angiotensin-(1–7)	Days 0, 3, 7, 11, 14, 18, 21, and 25
Brown et al., 1997 [26]	Single 1.5 × 1.5 cm full-thickness dorsal wound through panniculus	No splint; OpSite dressing	Main intervention, alone and in two-GF formulation	D/F	Human rhPDGF-BB in 5% PEG/PBS, alone or with rhIGF-II	Topical solution under OpSite	10 µg of rhPDGF-BB/wound/day; combination also contained 10 µg of rhIGF-II	Once daily on days 0–4	Vehicle; rhIGF-II alone; nondiabetic vehicle	Terminal treatment cohorts on days 5 and 10; natural history observations until day 28
Kiritsy et al., 1995 [27]	Single 1.5 × 1.5 cm full-thickness dorsal wound through panniculus	No splint; Tegaderm dressing	Main intervention, alone and in two-GF formulation	D/F	Human rhPDGF-BB alone or with rhIGF-I in methylcellulose	Topical gel under Tegaderm	PDGF-BB 4 or 40 µg/cm^2^; combination of PDGF-BB + IGF-I, 4 µg/cm^2^ each	Immediately and every other day until day 24 (13 applications)	Methylcellulose vehicle and PDGF-BB monotherapy	Twice weekly until complete healing, up to day 63
Brown et al., 1994 [28]	Single 1.5 × 1.5 cm full-thickness dorsal wound through panniculus	No splint; OpSite dressing	Main intervention, alone and in two-GF formulations	D/F	Human rhPDGF-BB in 5% PEG, alone or with EGF/TGF-α	Topical solution injected beneath OpSite	10 µg of rhPDGF-BB/wound/day; combinations contained 1 µg of EGF or TGF-α	Once daily on days 0–4	Vehicle; EGF; TGF-α monotherapies	Days 0, 3, 7, 11, 15, 18, and 21; histology on days 15 and 21
Greenhalgh et al., 1990 [29]	Single 1.5 × 1.5 cm full-thickness dorsal wound through panniculus	No splint; OpSite dressing	Main intervention, alone and with rbFGF	D/F	Yeast-derived human rPDGF-BB in 5% PEG/PBS, alone or with human rbFGF	Topical solution injected beneath OpSite	rPDGF-BB 1 or 10 µg/wound/day; rbFGF 0.4 or 1 µg/wound/day	Daily for 5, 10, or 14 days, depending on the experiment	Vehicle; rbFGF monotherapy; nondiabetic vehicle	Days 3, 5, 7, 10, and 21 in terminal experiments; serial follow-up to 6 weeks, with one wound observed until day 90

Note: D, effect directly attributable to rhPDGF-BB because a no-factor or otherwise equivalent comparator was available; F, effect attributable only to a multicomponent formulation containing rhPDGF-BB; D/F, article containing both comparison types. Active reference comparator describes the role of becaplermin/Regranex and is not a third attribution category. Dose information was reproduced as reported. Active rhPDGF-BB mass per wound was not inferred from a product name, formulation concentration, application volume, or fabrication batch loading when the information required for a valid conversion was unavailable. AREG, amphiregulin; CMC, carboxymethylcellulose; EGF, epidermal growth factor; GF, growth factor; HB-EGF, heparin-binding EGF-like growth factor; Hsp90α, heat-shock protein 90 alpha; IGF, insulin-like growth factor; i.p., intraperitoneal; MMP, matrix metalloproteinase; NR, not reported; PBS, phosphate-buffered saline; PEG, polyethylene glycol; PEI, polyethyleneimine; PlGF-2, placental growth factor 2; PLGA, poly(lactic-co-glycolic acid); rh, recombinant human; VEGF, vascular endothelial growth factor; WT, wild type.

**Table 3:** Outcome domains and assessment methods.

Study	Macroscopic Wound Healing	Histological Repair	Collagen and ECM Remodeling	Angiogenesis/ Perfusion	Inflammation/ Immune Response	Proliferation/ Molecular Signaling	Adverse Effects	Assessment Time Points
Fang et al., 2025 [8]	Serial digital photography; ImageJ residual wound area and closure.	H&E: epidermal integrity, granulation tissue, and inflammatory cell infiltration.	Masson’s trichrome: collagen deposition and maturation.	CD31 immunohistochemistry: vessel density.	H&E: neutrophil/lymphocyte accumulation.	Not assessed in vivo.	Local H&E screening for ulceration, erosion, and localized fibroblast proliferation; mortality/systemic effects NR.	Imaging: day 0 and every other day to day 18; tissue: day 18.
Deng et al., 2025 [9]	Digital photography; ImageJ wound area ratio and wound width.	H&E: epithelialization, epidermal thickness, skin appendages, and neutrophils.	Masson’s trichrome/ImageJ: collagen deposition and organization.	CD31 immunofluorescence: neovascularization.	CD206 immunofluorescence and H&E neutrophil assessment.	Not assessed in vivo.	NR; hemolysis testing was not an in vivo wound-model assessment.	Macroscopic: days 0, 3, 7, and 12; tissue: day 12; healed tissue: day 22.
Saklani et al., 2024 [20]	Serial digital photography; ImageJ percentage wound area.	H&E: re-epithelialization, inflammatory cells, and hair follicles.	Masson’s trichrome/ImageJ: collagen deposition.	CD31 immunohistochemistry/ImageJ: microvessel network.	H&E: inflammatory cell infiltration.	Not assessed in vivo.	OECD 402 dermal-toxicity observations, including body weight, general health, and behavior; no adverse effects reported.	Macroscopic: days 0, 2, 4, 7, 10, and 14; histology: days 4, 7, and 14 (normal) and 7 and 14 (diabetic).
Ganesh et al., 2023 [30]	Serial wound photographs; percentage closure.	H&E: wound gap, epithelial margins, fibrous cap, granulation tissue, and tissue architecture.	Masson’s trichrome: collagen deposition.	Not assessed in vivo.	Wound-tissue qPCR/immunoblotting: CD163, CD206, NLRP3, and ICAM-1.	Nrf2 immunohistochemistry; qPCR for NRF2, HO-1, SOD1, PPARγ, CD163, and CD206; immunoblotting for p-Akt/Akt, HO-1, CD206, NLRP3, and ICAM-1.	NR.	Macroscopic: days 0, 7, 10, and 14; tissue: day 14.
Alshoubaki et al., 2023 [31]	Not assessed separately; closure was measured histomorphometrically.	H&E histomorphometry: epithelial gap/closure, granulation tissue, and granulocytes.	Not assessed.	CD31 and desmin immunostaining: vessel abundance and maturity.	H&E: granulocyte infiltration.	Not assessed in the wound experiment.	NR for the wound experiment.	Day 9.
White et al., 2021 [32]	Not assessed by serial photography; wound extent was measured histomorphometrically.	H&E: wound extent, re-epithelialization, and granulation tissue.	Not assessed.	Flow cytometry: endothelial cell abundance.	Flow cytometry: neutrophils; M1-, M2-, and arginase-positive macrophages; and T-cell subsets.	Flow-cytometric assessment of Ki-67-positive proliferating cells in wound tissue (day 7).	NR.	Days 3 and 7; Ki-67: day 7.
Nguyen et al., 2018 [33]	Serial digital photography; ImageJ percentage residual wound area.	H&E: re-epithelialization.	In situ DQ-collagen/DQ-gelatin zymography and affinity proteomics: active MMP-8 and MMP-9.	Not assessed.	ROS bioluminescence and NF-κB p65 ELISA.	Not assessed separately.	No wound model adverse-effect assessment reported.	Macroscopic: days 0, 7, 10, and 14; NF-κB: days 1 and 2; ROS: days 3 and 7; histology/zymography: day 7 and histology also day 14.
Gallant-Behm et al., 2018 [34]	No serial macroscopic endpoint in the eligible diabetic mouse comparison; wound width was measured histomorphometrically.	H&E: wound width, re-epithelialization, and granulation tissue area/thickness.	Not assessed.	CD31 immunohistochemistry: endothelial cell/vessel area and particle measurements.	Not assessed directly.	ITGA5 immunohistochemistry at the wound edge and in blood vessels.	Plasma ALT, AST, BUN, and creatinine; no liver or kidney dysfunction attributed to treatment.	Histology/IHC: day 10; distribution and safety: 48 h after the final dose.
Ishihara et al., 2018 [35]	No serial photographic endpoint; closure was evaluated histomorphometrically.	H&E: re-epithelialization and granulation tissue area.	Not assessed.	Flow cytometry: CD31-positive/CD45-negative endothelial cells.	Flow cytometry: Ly6G-positive/CD11b-positive neutrophils and Ly6C-positive/CD11b-positive monocytes.	Ki67 within CD31-positive/CD45-negative endothelial cells.	NR.	Histology: days 4, 7, and 10; flow cytometry: day 5.
McLaughlin et al., 2017 [36]	Calibrated serial photography; ImageJ residual wound area.	H&E: epithelial thickness and general tissue pathology.	Not assessed.	VEGF immunohistochemistry: percentage of VEGF-positive vessels.	Toluidine-blue staining: mast-cell counts.	BrdU basal epithelial labeling index; PDGF and FGF-2 immunohistochemistry.	Body weight and glucose monitoring; H&E assessment for cell death/structural pathology.	Macroscopic: day 0 and every other day up to day 14; tissue: days 1, 2, and 4.
Lee et al., 2016 [10]	Serial wound images; ImageJ wound closure percentage.	H&E: re-epithelialization, stratum corneum, tissue integration, and inflammatory infiltrate.	Collagen I and MMP-9 immunofluorescence.	Not assessed.	H&E: qualitative inflammatory cell infiltration.	Not assessed separately.	Local scaffold integration and inflammatory response described; systemic adverse effects NR.	Macroscopic: days 0, 3, 7, and 14; histology/immunofluorescence: days 7 and 14.
Lee et al., 2015 [11]	Wound edge tracing on glass slides; planimetric wound area.	Histology: re-epithelialization, stratum corneum, and cellular infiltration.	MMP-9 immunofluorescence.	Qualitative neovascularization/endothelial lining on histology.	Qualitative inflammatory cell infiltration on histology.	Not assessed as a direct proliferation or signaling endpoint.	Local inflammatory response described; systemic adverse effects NR.	Macroscopic: days 0, 3, 7, and 14; histology/immunofluorescence: day 14.
O’Brien et al., 2014 [12]	Standardized digital photography; AlphaEase open-wound area and closure.	H&E and pan-keratin immunohistochemistry: epithelialization and rete-ridge formation.	Picrosirius red/polarized light: collagen and dermal organization.	PECAM-1 immunohistochemistry: capillary lumen density.	Calprotectin immunohistochemistry: macrophage counts.	Not assessed in vivo.	NR.	Macroscopic: days 0, 7, 14, and 21; becaplermin comparison: days 7 and 14; tissue: day 14.
Park et al., 2014 [13]	Masked ImageJ tracing of inner/outer wound margins; percentage closure and contraction.	H&E: re-epithelialization length, epithelial gap, granulation/fibrovascular proliferation, and repair score.	Picrosirius red/polarized light: collagen percentage.	Not assessed.	H&E: semiquantitative inflammatory infiltrate score.	Not assessed in vivo.	Daily monitoring of body weight, appetite, pain/general health, infection, and exudate; no treatment-related clinical toxicity reported.	Macroscopic: days 0, 2, 4, 6, 8, 10, and 11; tissue: day 11.
Allen et al., 2014 [14]	Standardized serial photography; calibrated percentage closure and time to complete closure.	Not assessed as a general repair endpoint.	Not assessed.	CD31 immunofluorescence; FITC-lectin perfusion; DiI-labeled progenitor cell incorporation.	Not assessed.	Flow cytometry of circulating lineage-negative/Sca-1-positive/c-kit-positive progenitor cells; DiI cell tracking.	NR.	Macroscopic: days 0, 7, 14, 21, and 28; progenitor cell flow cytometry: baseline and days 7, 14, and 21; vascular endpoints: days 7, 21, and 28.
Dwivedi and Chaudhary, 2012 [15]	Graphical planimetry: wound area/contraction; gross photographs.	H&E: fibrous tissue organization, epithelialization, scar formation, granulation, and semiquantitative repair scores.	Granulation-tissue hydroxyproline and hexosamine; histological collagen score.	Histological angiogenesis score.	H&E: neutrophils, cellular debris, and microabscesses; S. aureus colony count.	Histological squamous-cell proliferation score; tissue protein, nitrate, ascorbic acid, MDA, xanthine oxidase, catalase, and glutathione.	NR.	Macroscopic: days 0, 5, 10, and 14; tissue/biochemistry: day 14.
Berdal et al., 2011 [16]	Manual wound area measurement; percentage wound closure.	H&E: blinded Greenhalgh 1–12 healing score and granulation tissue maturity.	Masson’s trichrome: semiquantitative collagen deposition.	Qualitative capillarization within the histological granulation tissue assessment.	F4/80 immunohistochemistry: macrophages; wound culture for infection.	Not assessed.	Clinical signs and cultures for wound infection; infection incidence reported by group; other adverse effects NR.	Closure: seven assessments over days 0–17 (follow-up to day 45); histology: days 12–14 and 17–18.
Cheng et al., 2011 [17]	Standardized digital photography; AlphaEase/ImageJ percentage wound closure.	H&E and pan-keratin immunostaining: re-epithelialization.	α-SMA immunostaining: myofibroblast recruitment/contraction.	PECAM-1 immunostaining: endothelial cells/blood vessels.	Not assessed.	Not assessed as a direct proliferation or signaling endpoint.	NR.	Macroscopic: days 0, 5, 10, 14, and 18; tissue: day 14. Histologic assays were performed in the peptide/placebo experiment; the becaplermin head-to-head comparison was macroscopic.
Tkalčević et al., 2007 [18]	Gross closure status at terminal cohorts; quantitative macroscopic method NR.	H&E: semiquantitative immature/mature granulation tissue and fibrosis.	Van Gieson staining organized collagen.	Vascularity within granulation tissue scoring; desmin/α-SMA immunostaining of vessels/myofibroblasts.	Not assessed in the eligible excisional-wound experiment.	Desmin/α-SMA immunostaining; no in vivo EGR-1/NAB2 assay.	NR.	Terminal cohorts: days 6, 9, 12, 18, and 24; immunostaining: days 9 and 12.
Cheng et al., 2007 [19]	Wound edge tracing and computerized planimetry: wound area, re-epithelialized area, percentage closure, and contraction.	H&E: gross-to-microscopic repair morphology, epithelium, and granulation tissue.	Masson’s trichrome: collagen deposition and organization.	Factor VIII-related antigen immunohistochemistry: new capillary sprouts.	H&E: qualitative inflammatory cell infiltration.	PCNA immunohistochemistry: labeling index.	Body weight and blood glucose monitoring; treatment-related adverse effects NR.	Macroscopic/metabolic: days 0, 3, 7, 10, and 14; tissue: days 7 and 14.
Zhao et al., 2006 [21]	No serial macroscopic measurement; healing stage was determined microscopically.	H&E/Masson’s trichrome: re-epithelialization, cellularity, granulation-tissue thickness, and healing stage classification.	Masson’s trichrome: collagen deposition, orientation, and ECM maturation.	Qualitative vessel presence within histological sections; no dedicated vascular quantification.	H&E: qualitative inflammatory cell infiltration.	Not assessed in vivo.	No adverse-effect assessment in the eligible db/db experiment.	Day 11.
Ševeljević-Jaran et al., 2006 [22]	Standardized digital photography; Leica planimetry of wound area; blinded five-item macroscopic healing score.	H&E/morphometry: re-epithelialization, granulation tissue, necrosis, and inflammation.	Mallory staining: cellular, organized, and collagen-dominant granulation tissue categories.	Vessels included qualitatively in granulation tissue classification; no separate vascular quantification.	Histological necrosis/inflammation assessment.	Not assessed.	NR.	Macroscopic: days 0, 1, 4, and 6; 3- and 7-day wounds for histology (sacrifice on study days 4 and 8).
Chan et al., 2006 [23]	Digital photographs every 2–3 days; computerized planimetry, percentage closure, and time to 50%/complete closure.	H&E: granulation tissue and epithelial quality.	Not assessed.	Not assessed as a dedicated endpoint.	H&E: descriptive inflammatory infiltrate.	Not assessed.	NR.	Macroscopic: every 2–3 days for 3 weeks; early histology time point unclear (day 7 in the Methods and day 5 in the corresponding figure); late histology: day 24.
Uhl et al., 2003 [24]	Intravital fluorescence microscopy: wound surface area, re-epithelialization, and time to complete closure.	Not assessed by conventional histology.	Not assessed.	Intravital microscopy: revascularized area, vessel diameters, and permeability; laser Doppler red blood cell flux/perfusion.	Rhodamine 6G intravital microscopy: rolling and adherent leukocytes.	Not assessed.	Attrition and reasons recorded (severe hyperglycemia, weight loss/exsiccosis, or wound perforation); treatment-specific adverse effects NR.	Baseline and days 1, 3, 6, 9, 12, 18, and 24; laser Doppler also on day 9; daily checks until closure.
Rodgers et al., 2003 [25]	Blinded wound/epithelial edge tracing; graph paper planimetry of open area and complete closure.	H&E: appearance and re-epithelialization of newly formed tissue.	H&E: qualitative dermal fibrosis and adnexal regeneration.	H&E: qualitative vessel formation.	H&E: qualitative dermal inflammatory reaction.	Not assessed.	NR.	Macroscopic: days 0, 3, 11, 14, 18, 21, and 25; histology: necropsy/day 25.
Brown et al., 1997 [26]	Serial wound edge tracing (approximately twice weekly); image analysis planimetry and percentage closure.	H&E: 12-point score for cellular infiltration, granulation tissue, vascularity, and re-epithelialization.	H&E: collagen organization within the healing score.	H&E: vascularity within the healing score.	H&E: inflammatory cell infiltration.	In situ labeling of fragmented DNA: apoptosis score and distribution.	NR.	Natural history tissue: 0.5, 1, 3, 5, 10, 14, 20, and 28 days; growth factor cohorts: days 5 and 10; closure assessed serially.
Kiritsy et al., 1995 [27]	Standardized Polaroid photographs twice weekly; blinded planimetry, percentage closure, and median/mean time to complete healing.	Not assessed in the reported experiments.	Not assessed.	Not assessed.	Not assessed.	Not assessed.	Treatment tolerance recorded; late mortality occurred equally across groups and was attributed to age/frequent anesthesia.	Twice weekly until complete healing, up to day 63.
Brown et al., 1994 [28]	Serial epithelial edge tracing; image analysis planimetry and percentage closure.	H&E: 12-point score for cellular entry, granulation tissue, vascularity, collagen, and re-epithelialization.	Trichrome staining and histological scoring: collagen deposition/maturation.	Vascularity within the H&E healing score.	Cellular/inflammatory infiltration within the H&E healing score.	Not assessed.	NR.	Macroscopic: days 0, 3, 7, 11, 15, 18, and 21; histology: days 15 and 21.
Greenhalgh et al., 1990 [29]	Serial wound edge tracing through OpSite; computerized planimetry, percentage closure, and contraction estimate.	H&E/Masson’s trichrome: blinded 12-point score for cellular invasion, granulation tissue, vascularity, and re-epithelialization.	Masson’s trichrome and collagen component of the healing score.	Histological capillary ingrowth/vascularity within the healing score.	Histological inflammatory cell infiltration.	Not assessed.	Body weight, food intake, and general tolerance monitored; no gross procedural problems reported.	Terminal experiments: days 3, 5, 7, 10, and 21; wounds checked 3–5 times weekly for up to 6 weeks; one chronic wound followed to day 90.

Note: The table reports the outcome domains and methods used, not effect direction or magnitude. Histological measurement approaches are distinguished as qualitative, semiquantitative, or quantitative according to the methods reported in the original study. Qualitative indicates descriptive morphology without numerical scoring; semiquantitative indicates ordinal or composite scoring; and quantitative indicates continuous histomorphometric or image analysis measurement. When the original report did not provide sufficient methodological detail, the assessment method was retained as reported and was not inferred. Not assessed indicates that the domain was not evaluated in the eligible in vivo wound experiment; NR indicates insufficient or absent reporting. α-SMA, alpha-smooth-muscle actin; ALT, alanine aminotransferase; AST, aspartate aminotransferase; BrdU, bromodeoxyuridine; BUN, blood urea nitrogen; ECM, extracellular matrix; FGF, fibroblast growth factor; H&E, hematoxylin and eosin; HO-1, heme oxygenase-1; ICAM-1, intercellular adhesion molecule 1; IF, immunofluorescence; IHC, immunohistochemistry; ITGA5, integrin alpha 5; MDA, malondialdehyde; MMP, matrix metalloproteinase; NF-κB, nuclear factor kappa B; Nrf2, nuclear factor erythroid 2-related factor 2; PCNA, proliferating cell nuclear antigen; PECAM-1, platelet endothelial cell adhesion molecule 1; qPCR, quantitative polymerase chain reaction; ROS, reactive oxygen species; SOD1, superoxide dismutase 1; VEGF, vascular endothelial growth factor.

**Table 4:** Attribution-specific summary of the quantitative and structured descriptive evidence.

Evidence Level	Defining Intervention–Comparator Contrast	Evidence Base	Main Findings	Permitted Interpretation
Direct (D)	rhPDGF-BB, becaplermin, or Regranex versus untreated, placebo, vehicle, factor-free control, or a matched formulation lacking rhPDGF-BB.	26 studies [9,10,11,12,13,14,15,17,18,19,20,21,22,23,24,25,26,27,28,29,30,31,32,33,34,36]; 17 D-only and 9 D/F.	Both meta-analyses included only D contrasts. Percentage wound closure: 3 studies, 113 animals; MD: 12.94 percentage points (95% CI: −0.34 to 26.23). Time to complete closure: 2 studies, 45 animals; MD: −4.28 days (95% CI: −27.98 to 19.42). Favorable directions: macroscopic healing, 17/22; histological repair, 11/21; collagen/ECM, 9/15; angiogenesis/perfusion, 8/17; inflammation/immune response, 4/19; proliferation/molecular signaling, 2/9; adverse effects, 0/10.	Permits attribution of the between-group difference to the specific contribution of rhPDGF-BB relative to a factor-free comparator.
Formulation level (F)	Engineered rhPDGF-BB variants, rhPDGF-BB-containing delivery systems, or combinations with another biologically active intervention without a matched formulation-minus-factor comparator.	12 studies [8,9,14,16,26,27,28,29,31,32,34,35]; 3 F-only and 9 D/F.	No formulation-level findings were quantitatively pooled. Favorable directions: macroscopic healing, 8/8; histological repair, 9/10; collagen/ECM, 3/4; angiogenesis/perfusion, 7/9; inflammation/immune response, 3/7; proliferation/molecular signaling, 2/3; adverse effects, 0/4.	The between-group difference applies to the complete formulation or combination; the independent contribution of rhPDGF-BB cannot be isolated.

Note: CI, confidence interval; D, directly attributable evidence; D/F, studies contributing both directly attributable and formulation-level evidence; ECM, extracellular matrix; F, formulation-level evidence; MD, mean difference; rhPDGF-BB, recombinant human platelet-derived growth factor-BB. Effect direction fractions represent the number of studies showing a favorable direction divided by the number of studies with an eligible finding in that domain. These descriptive classifications do not incorporate effect magnitude, precision, statistical significance, sample size, risk of bias, or certainty of evidence. The 70% concordance threshold was prespecified for exploratory descriptive interpretation.

**Table 5:** Summary of findings and GRADE certainty assessment.

Outcome	Studies and Animals	Pooled Effect	GRADE Assessment	Certainty	Interpretation
Percentage wound closure at days 10–12 (higher values indicate greater wound closure)	3 studies 113 animals 60 rhPDGF-BB 53 controls	MD: 12.94 percentage points (95% CI: −0.34 to 26.23)	Risk of bias: Serious (−1) ^a^ Inconsistency: Not serious ^b^ Imprecision: Serious (−1) ^c^ Indirectness: Serious (−1) ^d^ Publication bias: No downgrade ^e^ Upgrading factors: None ^f^	**Very low** ⊕◯◯◯	The evidence is very uncertain regarding the effect of rhPDGF-BB on percentage wound closure at days 10–12.

^a^ The methods used to generate the allocation sequence were not reproducibly described in any of the three studies. Allocation concealment and several other safeguards were incompletely reported, and Deng et al.’s study [9], which contributed 32.0% of the model weight, was judged as high-risk-of-bias because of incomplete outcome data. One level, rather than two, was deducted because Greenhalgh et al. [29] contributed 61.5% of the weight and used coded and blinded procedures. ^b^ Although I^2^ was 54.6%, the confidence intervals of all three study estimates overlapped. Deng et al. [9] and Greenhalgh et al. [29] reported similar favorable estimates and accounted for 93.5% of the model weight. The discordant estimate from Park et al.’s study [13] was imprecise, and the sensitivity analysis retained a favorable pooled direction. ^c^ Only three studies and 113 animals contributed to the analysis. The 95% confidence interval crossed the null effect and was compatible with both little or no benefit and potentially important benefit. The sensitivity analysis produced a wider interval ranging from −9.26 to 26.68 percentage points. ^d^ All contributing studies used mice with experimentally created acute full-thickness dorsal wounds. These models incompletely represent the chronic and multifactorial characteristics of human diabetic foot ulcers. ^e^ Funnel plot asymmetry and statistical small-study-effect tests were not appropriate because only three studies were available. The comprehensive search identified both favorable and discordant findings; therefore, publication bias was not considered sufficiently evident to justify downgrading, although it cannot be excluded. ^f^ The evidence did not demonstrate a sufficiently large and robust effect, a consistent dose–response relationship, an opposing effect of plausible residual confounding, or reproducibility across independent animal species to justify upgrading. Abbreviations: CI, confidence interval; GRADE, Grading of Recommendations Assessment, Development and Evaluation; MD, mean difference; rhPDGF-BB, recombinant human platelet-derived growth factor-BB. GRADE certainty symbols: ⊕, one level of certainty; ◯, one unfilled level. Accordingly, ⊕◯◯◯ indicates very-low-certainty evidence.

## Data Availability

No new data were created or analyzed in this study. Data sharing is not applicable.

## Supplementary Materials

The following supporting information can be downloaded at https://www.mdpi.com/article/10.3390/ph19091497/s1. Table S1: Detailed experimental groups and sample sizes; Table S2: Study-level reporting of in vivo safety, tolerability, attrition, and systemic assessments; Table S3: Robustness and influence diagnostics for the primary meta-analysis of percentage wound closure at days 10–12; Table S4: Study-level context for the exploratory synthesis of directly attributable macroscopic wound-healing findings; Table S5: Complete electronic search strategies used in the final search update. All databases were searched from inception or the earliest available coverage to 29 July 2026. Tables S1–S4 summarize data from the included studies [8,9,10,11,12,13,14,15,16,17,18,19,20,21,22,23,24,25,26,27,28,29,30,31,32,33,34,35,36].

## Abbreviations

The following abbreviations are used in this manuscript:

AGEs	Advanced glycation end products
AKT	Protein kinase B
ALT	Alanine aminotransferase
ARRIVE	Animal Research: Reporting of In Vivo Experiments
AST	Aspartate aminotransferase
BrdU	5-bromo-2′-deoxyuridine
BUN	Blood urea nitrogen
CD	Cluster of differentiation
CI	Confidence interval
CMC	Carboxymethyl cellulose
DAG	Diacylglycerol
DNA	Deoxyribonucleic acid
ECM	Extracellular matrix
EGF	Epidermal growth factor
EGR-1	Early growth response protein 1
ERK	Extracellular signal-regulated kinase
FGF	Fibroblast growth factor
GRADE	Grading of Recommendations Assessment, Development and Evaluation
HB-EGF	Heparin-binding epidermal growth factor
HIF-1α	Hypoxia-inducible factor 1 alpha
HO-1	Heme oxygenase 1
IF	Immunofluorescence
IGF-I	Insulin-like growth factor I
IHC	Immunohistochemistry
IL	Interleukin
IP_3_	Inositol 1,4,5-trisphosphate
IWGDF	International Working Group on the Diabetic Foot
MAPK	Mitogen-activated protein kinase
MD	Mean difference
MDA	Malondialdehyde
MEK	Mitogen-activated protein kinase kinase
MeSH	Medical Subject Headings
MMP	Matrix metalloproteinase
NF-κB	Nuclear factor kappa B
NLRP3	NLR family pyrin domain-containing protein 3
NOD	Non-obese diabetic
NR	Not reported
Nrf2	Nuclear factor erythroid 2-related factor 2
OSF	Open Science Framework
PBS	Phosphate-buffered saline
PCNA	Proliferating cell nuclear antigen
PDGF	Platelet-derived growth factor
PDGF-BB	Platelet-derived growth factor-BB
PDGFR	Platelet-derived growth factor receptor
PEG	Polyethylene glycol
PICOS	Population, Intervention, Comparator, Outcomes, and Study Design
PI3K	Phosphoinositide 3-kinase
PIP_3_	Phosphatidylinositol 3,4,5-trisphosphate
PLGA	Poly(lactic-co-glycolic acid)
PLC-γ	Phospholipase C gamma
PRISMA	Preferred Reporting Items for Systematic Reviews and Meta-Analyses
RAF	Rapidly accelerated fibrosarcoma kinase
RAGE	Receptor for advanced glycation end-products
RAS	Rat sarcoma small gtpase
REML	Restricted maximum likelihood
rhPDGF-BB	Recombinant human platelet-derived growth factor-BB
ROS	Reactive oxygen species
SOD1	Superoxide dismutase 1
STZ	Streptozotocin
SWiM	Synthesis Without Meta-Analysis
SYRCLE	Systematic Review Centre for Laboratory Animal Experimentation
TGF	Transforming growth factor
TIMP	Tissue inhibitor of metalloproteinases
VEGF	Vascular endothelial growth factor
WRAHPS	Wound Reporting in Animal and Human Preclinical Studies

## References

[1] Armstrong D.G., Tan T.W., Boulton A.J.M., Bus S.A. (2023). Diabetic Foot Ulcers: A Review. JAMA.

[2] Xiong Y., Knoedler S., Alfertshofer M., Kim B.S., Jiang D., Liu G., Rinkevich Y., Mi B. (2025). Mechanisms and Therapeutic Opportunities in Metabolic Aberrations of Diabetic Wounds: A Narrative Review. Cell Death Dis..

[3] Smith J., Rai V. (2024). Novel Factors Regulating Proliferation, Migration, and Differentiation of Fibroblasts, Keratinocytes, and Vascular Smooth Muscle Cells during Wound Healing. Biomedicines.

[4] Wieman T.J., Smiell J.M., Su Y. (1998). Efficacy and Safely of a Topical Gel Formulation of Recombinant Human Platelet-Derived Growth Factor-BB (Becaplermin) in Patients with Chronic Neuropathic Diabetic Ulcers: A Phase III Randomized Placebo-Controlled Double-Blind Study. Diabetes Care.

[5] Chen P., Vilorio N.C., Dhatariya K., Jeffcoate W., Lobmann R., McIntosh C., Piaggesi A., Steinberg J., Vas P., Viswanathan V. (2024). Guidelines on Interventions to Enhance Healing of Foot Ulcers in People with Diabetes (IWGDF 2023 Update). Diabetes Metab. Res. Rev..

[6] Yager D.R., Chen S.M., Ward S.I., Olutoye O.O., Diegelmann R.F., Cohen I.K. (1997). Ability of Chronic Wound Fluids to Degrade Peptide Growth Factors Is Associated with Increased Levels of Elastase Activity and Diminished Levels of Proteinase Inhibitors. Wound Repair Regen..

[7] Shan B.H., Wu F.G. (2024). Hydrogel-Based Growth Factor Delivery Platforms: Strategies and Recent Advances. Adv. Mater..

[8] Fang Y.-P., Wu Y.-C., Liao W.-T., Chuang C.-H., Liu K.-F., Chen R.-F., Kuo Y.-R. (2025). A Conductive Polypyrrole-Based Thermosensitive Hydrogel Dressing Incorporating RhPDGF-BB for Enhanced Diabetic Wound Healing. Eur. J. Pharm. Sci..

[9] Deng H., Wang F., Zhou Y., Lei H., Zhou H., Chen S., Meng Z., He M., Tu D., Wang H. (2025). Biosynthesis of a Dual Growth Factors (GFs) Functionalized Silk Sericin Hydrogel to Promote Chronic Wound Healing in Diabetic Mice. Bioact. Mater..

[10] Lee C.-H., Chao Y.-K., Chang S.-H., Chen W.-J., Hung K.-C., Liu S.-J., Juang J.-H., Chen Y.-T., Wang F.-S. (2016). Nanofibrous RhPDGF-Eluting PLGA-Collagen Hybrid Scaffolds Enhance Healing of Diabetic Wounds. RSC Adv..

[11] Lee C.-H., Liu K.-S., Chang S.-H., Chen W.-J., Hung K.-C., Liu S.-J., Pang J.-H.S., Juang J.-H., Chou C.-C., Chang P.-C. (2015). Promoting Diabetic Wound Therapy Using Biodegradable RhPDGF-Loaded Nanofibrous Membranes CONSORT-Compliant Article. Medicine.

[12] O’Brien K., Bhatia A., Tsen F., Chen M., Wong A.K., Woodley D.T., Li W. (2014). Identification of the Critical Therapeutic Entity in Secreted Hsp90α That Promotes Wound Healing in Newly Re-Standardized Healthy and Diabetic Pig Models. PLoS ONE.

[13] Park S.A., Raghunathan V.K., Shah N.M., Teixeira L., Motta M.J., Covert J., Dubielzig R., Schurr M., Isseroff R.R., Abbott N.L. (2014). PDGF-BB Does Not Accelerate Healing in Diabetic Mice with Splinted Skin Wounds. PLoS ONE.

[14] Allen R.J., Soares M.A., Haberman I.D., Szpalski C., Schachar J., Lin C.D., Nguyen P.D., Saadeh P.B., Warren S.M. (2014). Combination Therapy Accelerates Diabetic Wound Closure. PLoS ONE.

[15] Dwivedi V.K., Chaudhary M. (2012). Comparative Wound Healing Efficacy of Ampucare and Becaplermin in Diabetic Rat. Afr. J. Pharm. Pharmacol..

[16] Berdal M., Appelbom H.I., Eikrem J.H., Lund A., Busund L.-T., Hanes R., Seljelid R., Jenssen T. (2011). Aminated β-1,3-D-Glucan Has a Dose-Dependent Effect on Wound Healing in Diabetic Db/Db Mice. Wound Repair Regen..

[17] Cheng C.-F., Sahu D., Tsen F., Zhao Z., Fan J., Kim R., Wang X., O’Brien K., Li Y., Kuang Y. (2011). A Fragment of Secreted Hsp90α Carries Properties That Enable It to Accelerate Effectively Both Acute and Diabetic Wound Healing in Mice. J. Clin. Investig..

[18] Tkalčević V.I., Čužić S., Brajša K., Mildner B., Bokulić A., Šitum K., Perović D., Glojnarić I., Parnham M.J. (2007). Enhancement by PL 14736 of Granulation and Collagen Organization in Healing Wounds and the Potential Role of Egr-1 Expression. Eur. J. Pharmacol..

[19] Cheng B., Liu H.-W., Fu X.-B., Sun T.-Z., Sheng Z.-Y. (2007). Recombinant Human Platelet-Derived Growth Factor Enhanced Dermal Wound Healing by a Pathway Involving ERK and c-Fos in Diabetic Rats. J. Dermatol. Sci..

[20] Saklani M., Jha C.B., Baidya A.T.K., Singh S., Kumar R., Mathur R., Tiwari A.K., Varshney R. (2024). Laminin Mimetic Angiogenic and Collagen Peptide Hydrogel for Enhance Dermal Wound Healing. Biomater. Adv..

[21] Zhao H., Mortezaei R., Wang Y., Sheng X., Aria F., Bojanowski K. (2006). SBD.4 Stimulates Regenerative Processes in Vitro, and Wound Healing in Genetically Diabetic Mice and in Human Skin/Severe-Combined Immunodeficiency Mouse Chimera. Wound Repair Regen..

[22] Ševeljević-Jaran D., Čužić S., Dominis-Kramarić M., Glojnarić I., Ivetić V., Radošević S., Parnham M.J. (2006). Accelerated Healing of Excisional Skin Wounds by PL 14736 in Alloxan-Hyperglycemic Rats. Skin Pharmacol. Physiol..

[23] Chan R.K., Liu P.H., Pietramaggiori G., Ibrahim S.I., Hechtman H.B., Orgill D.P. (2006). Effect of Recombinant Platelet-Derived Growth Factor (Regranex^®^) on Wound Closure in Genetically Diabetic Mice. J. Burn Care Res..

[24] Uhl E., Rösken F., Sirsjö A., Messmer K. (2003). Influence of Platelet-Derived Growth Factor on Microcirculation during Normal and Impaired Wound Healing. Wound Repair Regen..

[25] Rodgers K.E., Espinoza T., Felix J., Roda N., Maldonado S., DiZerega G. (2003). Acceleration of Healing, Reduction of Fibrotic Scar, and Normalization of Tissue Architecture by an Angiotensin Analogue, NorLeu3-A(1-7). Plast. Reconstr. Surg..

[26] Brown D.L., Kao W.W.-Y., Greenhalgh D.G. (1997). Apoptosis Down-Regulates Inflammation under the Advancing Epithelial Wound Edge: Delayed Patterns in Diabetes and Improvement with Topical Growth Factors. Surgery.

[27] Kiritsy C.P., Antoniades H.N., Carlson M.R., Beaulieu M.T., D’Andrea M., Lynch S.E. (1995). Combination of Platelet-Derived Growth Factor-BB and Insulin-like Growth Factor-I Is More Effective than Platelet-Derived Growth Factor-BB Alone in Stimulating Complete Healing of Full-Thickness Wounds in “Older” Diabetic Mice. Wound Repair Regen..

[28] Brown R.L., Breeden M.P., Greenhalgh D.G. (1994). PDGF and TGF-α Act Synergistically to Improve Wound Healing in the Genetically Diabetic Mouse. J. Surg. Res..

[29] Greenhalgh D.G., Sprugel K.H., Murray M.J., Ross R. (1990). PDGF and FGF Stimulate Wound Healing in the Genetically Diabetic Mouse. Am. J. Pathol..

[30] Ganesh G.V., Ramkumar K.M. (2023). Pterostilbene Accelerates Wound Healing Response in Diabetic Mice through Nrf2 Regulation. Mol. Immunol..

[31] Alshoubaki Y.K., Lu Y.-Z., Legrand J.M.D., Karami R., Fossat M., Salimova E., Julier Z., Martino M.M. (2023). A Superior Extracellular Matrix Binding Motif to Enhance the Regenerative Activity and Safety of Therapeutic Proteins. npj Regen. Med..

[32] White M.J.V., Briquez P.S., White D.A.V., Hubbell J.A. (2021). VEGF-A, PDGF-BB and HB-EGF Engineered for Promiscuous Super Affinity to the Extracellular Matrix Improve Wound Healing in a Model of Type 1 Diabetes. npj Regen. Med..

[33] Nguyen T.T., Ding D., Wolter W.R., Pérez R.L., Champion M.M., Mahasenan K.V., Hesek D., Lee M., Schroeder V.A., Jones J.I. (2018). Validation of Matrix Metalloproteinase-9 (MMP-9) as a Novel Target for Treatment of Diabetic Foot Ulcers in Humans and Discovery of a Potent and Selective Small-Molecule MMP-9 Inhibitor That Accelerates Healing. J. Med. Chem..

[34] Gallant-Behm C.L., Piper J., Dickinson B.A., Dalby C.M., Pestano L.A., Jackson A.L. (2018). A Synthetic MicroRNA-92a Inhibitor (MRG-110) Accelerates Angiogenesis and Wound Healing in Diabetic and Nondiabetic Wounds. Wound Repair Regen..

[35] Ishihara J., Ishihara A., Fukunaga K., Sasaki K., White M.J.V., Briquez P.S., Hubbell J.A. (2018). Laminin Heparin-Binding Peptides Bind to Several Growth Factors and Enhance Diabetic Wound Healing. Nat. Commun..

[36] McLaughlin P.J., Cain J.D., Titunick M.B., Sassani J.W., Zagon I.S. (2017). Topical Naltrexone Is a Safe and Effective Alternative to Standard Treatment of Diabetic Wounds. Adv. Wound Care.

[37] Zhao X.H., Gu H.F., Xu Z.R., Zhang Q., Lv X.Y., Zheng X.J., Yang Y.M. (2014). Efficacy of Topical Recombinant Human Platelet-Derived Growth Factor for Treatment of Diabetic Lower-Extremity Ulcers: Systematic Review and Meta-Analysis. Metabolism.

[38] Thanigaimani S., Jin H., Ahmad U., Anbalagan R., Golledge J. (2023). Comparative Efficacy of Growth Factor Therapy in Healing Diabetes-Related Foot Ulcers: A Network Meta-Analysis of Randomized Controlled Trials. Diabetes Metab. Res. Rev..

[39] Galiano R.D., Michaels V.J., Dobryansky M., Levine J.P., Gurtner G.C. (2004). Quantitative and Reproducible Murine Model of Excisional Wound Healing. Wound Repari Regen..

[40] Couturier A., Calissi C., Cracowski J.L., Sigaudo-Roussel D., Khouri C., Roustit M. (2023). Mouse Models of Diabetes-Related Ulcers: A Systematic Review and Network Meta-Analysis. eBioMedicine.

[41] Zhang Y., Leng K., Dong M., Wu J., Wang Z., Zhu Q., Gao X., Sun Y. (2026). Establishment and Translational Evaluation of Animal Models for Skin Wound Healing: A Systematic Review. Front. Physiol..

[42] Rajkumar V.S., Shiwen X., Bostrom M., Leoni P., Muddle J., Ivarsson M., Gerdin B., Denton C.P., Bou-Gharios G., Black C.M. (2006). Platelet-Derived Growth Factor-β Receptor Activation Is Essential for Fibroblast and Pericyte Recruitment during Cutaneous Wound Healing. Am. J. Pathol..

[43] Silveira L.L., Sarandy M.M., Novaes R.D., Morais-Santos M., Gonçalves R.V. (2024). OxInflammation Affects Transdifferentiation to Myofibroblasts, Prolonging Wound Healing in Diabetes: A Systematic Review. Int. J. Mol. Sci..

[44] Kanta J., Zavadakova A., Sticova E., Dubsky M. (2023). Fibronectin in Hyperglycaemia and Its Potential Use in the Treatment of Diabetic Foot Ulcers: A Review. Int. Wound J..

[45] Liu Y., Min D., Bolton T., Nubé V., Twigg S.M., Yue D.K., Mclennan S.V. (2009). Increased Matrix Metalloproteinase-9 Predicts Poor Wound Healing in Diabetic Foot Ulcers. Diabetes Care.

[46] Andrae J., Gallini R., Betsholtz C. (2008). Role of Platelet-Derived Growth Factors in Physiology and Medicine. Genes Dev..

[47] Kemp S.S., Aguera K.N., Cha B., Davis G.E. (2020). Defining Endothelial Cell-Derived Factors That Promote Pericyte Recruitment and Capillary Network Assembly. Arterioscler. Thromb. Vasc. Biol..

[48] Mace K.A., Yu D.H., Paydar K.Z., Boudreau N., Young D.M. (2007). Sustained Expression of Hif-1alpha in the Diabetic Environment Promotes Angiogenesis and Cutaneous Wound Repair. Wound Repair Regen..

[49] Clayton S.M., Shafikhani S.H., Soulika A.M. (2024). Macrophage and Neutrophil Dysfunction in Diabetic Wounds. Adv. Wound Care.

[50] Wolf S.J., Audu C.O., Moon J.Y., Joshi A.D., Melvin W.J., Barrett E.C., Mangum K., de Jimenez G.S., Rocco S., Buckley S. (2024). Diabetic Wound Keratinocytes Induce Macrophage JMJD3-Mediated Nlrp3 Expression via IL-1R Signaling. Diabetes.

[51] Dinh T., Braunagel S., Rosenblum B.I. (2015). Growth Factors in Wound Healing: The Present and the Future?. Clin. Podiatr. Med. Surg..

[52] Deng X., Gould M., Ali M.A. (2022). A Review of Current Advancements for Wound Healing: Biomaterial Applications and Medical Devices. J. Biomed. Mater. Res. B Appl. Biomater..

[53] Zhang Y., Lin S., Xu X., Yao Y., Feng S., Jiang S., Wang Y., He W., Mo R. (2025). Programmable Hierarchical Hydrogel Dressing for Sequential Release of Growth Factor and DNase to Accelerate Diabetic Wound Healing. J. Control. Release.

[54] Ziyadeh N., Fife D., Walker A.M., Wilkinson G.S., Seeger J.D. (2011). A Matched Cohort Study of the Risk of Cancer in Users of Becaplermin. Adv. Skin Wound Care.

[55] Slade H.B., Lynch S.E., Dickerson J.E. (2025). Safety of up to 140 Daily Applications of Recombinant Human Platelet-Derived Growth Factor (RhPDGF-BB) Onto Skin Wounds: Unboxing the Evidence. Wound Repair Regen..

[56] Chen M., Chang C., Levian B., Woodley D.T., Li W. (2023). Why Are There So Few FDA-Approved Therapeutics for Wound Healing?. Int. J. Mol. Sci..

[57] Eming S.A., Martin P., Tomic-Canic M. (2014). Wound Repair and Regeneration: Mechanisms, Signaling, and Translation. Sci. Transl. Med..

[58] Theocharidis G., Thomas B.E., Sarkar D., Mumme H.L., Pilcher W.J.R., Dwivedi B., Sandoval-Schaefer T., Sîrbulescu R.F., Kafanas A., Mezghani I. (2022). Single Cell Transcriptomic Landscape of Diabetic Foot Ulcers. Nat. Commun..

[59] Wilkinson H.N., Hardman M.J. (2020). Wound Healing: Cellular Mechanisms and Pathological Outcomes. Open Biol..

[60] Boeringer T., Gould L.J., Koria P. (2020). Protease-Resistant Growth Factor Formulations for the Healing of Chronic Wounds. Adv. Wound Care.

[61] Wang R., Gu S., Kim Y.H., Lee A., Lin H., Jiang D. (2025). Diabetic Wound Repair: From Mechanism to Therapeutic Opportunities. MedComm.

[62] du Sert N.P., Hurst V., Ahluwalia A., Alam S., Avey M.T., Baker M., Browne W.J., Clark A., Cuthill I.C., Dirnagl U. (2020). The ARRIVE Guidelines 2.0: Updated Guidelines for Reporting Animal Research. PLoS Biol..

[63] Ojeh N., Vecin N.M., Pastar I., Volk S.W., Wilgus T., Griffiths S., Ramey-Ward A.N., Driver V.R., DiPietro L.A., Gould L.J. (2025). The Wound Reporting in Animal and Human Preclinical Studies Guidelines. Wound Repair Regen..

[64] Page M.J., McKenzie J.E., Bossuyt P.M., Boutron I., Hoffmann T.C., Mulrow C.D., Shamseer L., Tetzlaff J.M., Akl E.A., Brennan S.E. (2021). The PRISMA 2020 Statement: An Updated Guideline for Reporting Systematic Reviews. BMJ.

[65] Hooijmans C.R., Rovers M.M., De Vries R.B.M., Leenaars M., Ritskes-Hoitinga M., Langendam M.W. (2014). SYRCLE’s Risk of Bias Tool for Animal Studies. BMC Med. Res. Methodol..

[66] Boon M.H., Thomson H. (2021). The Effect Direction Plot Revisited: Application of the 2019 Cochrane Handbook Guidance on Alternative Synthesis Methods. Res. Synth. Methods.

[67] Campbell M., McKenzie J.E., Sowden A., Katikireddi S.V., Brennan S.E., Ellis S., Hartmann-Boyce J., Ryan R., Shepperd S., Thomas J. (2020). Synthesis without Meta-Analysis (SWiM) in Systematic Reviews: Reporting Guideline. BMJ.

[68] Hooijmans C.R., De Vries R.B.M., Ritskes-Hoitinga M., Rovers M.M., Leeflang M.M., IntHout J., Wever K.E., Hooft L., de Beer H., Kuijpers T. (2018). Facilitating Healthcare Decisions by Assessing the Certainty in the Evidence from Preclinical Animal Studies. PLoS ONE.

