# Supplementary material for: Preclinical Efficacy of Recombinant Human Platelet-Derived Growth Factor-BB in Experimental Diabetic Wound Healing: A Systematic Review and Meta-Analysis of Animal Studies
Source: Pharmaceuticals (Basel). 2026 Sep 21;19(9):1497. doi: 10.3390/ph19091497 (PMC13610357; doi:10.3390/ph19091497)
Supplement: Supplementary file 1 [file pharmaceuticals-19-01497-s001.zip › Table_S5.pdf]

Table S5. Complete electronic search strategies used in the final search update. All databases were searched from inception or earliest available coverage through 29 July 2026.

Database	Search strategy	Search date	Records retrieved (n)
PubMed	("Becaplermin"[Mesh] OR "Platelet-Derived Growth Factor"[Mesh] OR becaplermin[tiab] OR Regranex[tiab] OR "platelet-derived growth factor BB"[tiab] OR "platelet derived growth factor BB"[tiab] OR "platelet-derived growth factor-BB"[tiab] OR "recombinant human platelet-derived growth factor"[tiab] OR "recombinant human platelet derived growth factor"[tiab] OR "recombinant platelet-derived growth factor"[tiab] OR "recombinant platelet derived growth factor"[tiab] OR "PDGF-BB"[tiab] OR "PDGF BB"[tiab] OR PDGFBB[tiab] OR "rhPDGF-BB"[tiab] OR "rhPDGF BB"[tiab] OR rhPDGFBB[tiab] OR "rPDGF-BB"[tiab] OR "rPDGF BB"[tiab] OR rPDGFBB[tiab] OR rhPDGF[tiab] OR rPDGF[tiab] OR PDGF[tiab])<br>AND<br>("Diabetes Mellitus"[Mesh] OR "Hyperglycemia"[Mesh] OR diabet*[tiab] OR hyperglyc*[tiab] OR "db/db"[tiab] OR "db db"[tiab] OR "ob/ob"[tiab] OR "ob ob"[tiab] OR streptozotocin[tiab] OR STZ[tiab] OR alloxan[tiab] OR Akita[tiab] OR "Zucker diabetic fatty"[tiab] OR ZDF[tiab] OR "Goto-Kakizaki"[tiab] OR "high-fat diet"[tiab] OR HFD[tiab])<br>AND<br>("Wound Healing"[Mesh] OR "Skin Ulcer"[Mesh] OR "Diabetic Foot"[Mesh] OR "Surgical Wound"[Mesh] OR wound*[tiab] OR ulcer*[tiab] OR "skin defect*"[tiab] OR "cutaneous defect*"[tiab] OR "skin injur*"[tiab] OR "cutaneous injur*"[tiab] OR "tissue repair"[tiab] OR reepithelial*[tiab] OR re-epithelial*[tiab])	29 July 2026	398
Embase	('becaplermin'/exp OR 'platelet derived growth factor'/exp OR becaplermin:ti,ab,kw OR regranex:ti,ab,kw OR 'platelet derived growth factor bb':ti,ab,kw OR 'platelet-derived growth factor bb':ti,ab,kw OR 'platelet-derived growth factor-bb':ti,ab,kw OR 'recombinant human platelet-derived growth factor':ti,ab,kw OR 'recombinant human platelet derived growth factor':ti,ab,kw OR 'recombinant platelet-derived growth factor':ti,ab,kw OR 'recombinant platelet derived growth factor':ti,ab,kw OR 'pdgf-bb':ti,ab,kw OR 'pdgf bb':ti,ab,kw OR pdgfbb:ti,ab,kw OR 'rhpdgf-bb':ti,ab,kw OR 'rhpdgf bb':ti,ab,kw OR rhpdgfbb:ti,ab,kw OR 'rpdgf-bb':ti,ab,kw OR 'rpdgf bb':ti,ab,kw OR rpdgfbb:ti,ab,kw OR rhpdgf:ti,ab,kw OR rpdgf:ti,ab,kw OR pdgf:ti,ab,kw)<br>AND<br>('diabetes mellitus'/exp OR 'hyperglycemia'/exp OR diabet*:ti,ab,kw OR hyperglyc*:ti,ab,kw OR 'db/db':ti,ab,kw OR 'db db':ti,ab,kw OR 'ob/ob':ti,ab,kw OR 'ob ob':ti,ab,kw OR streptozotocin:ti,ab,kw OR stz:ti,ab,kw OR alloxan:ti,ab,kw OR akita:ti,ab,kw OR 'zucker diabetic fatty':ti,ab,kw OR zdf:ti,ab,kw OR 'goto-kakizaki':ti,ab,kw OR 'high-fat diet':ti,ab,kw OR hfd:ti,ab,kw)<br>AND<br>('wound healing'/exp OR 'skin ulcer'/exp OR 'diabetic foot'/exp OR 'surgical wound'/exp OR wound*:ti,ab,kw OR ulcer*:ti,ab,kw OR 'skin defect*':ti,ab,kw OR 'cutaneous defect*':ti,ab,kw OR 'skin injur*':ti,ab,kw OR 'cutaneous injur*':ti,ab,kw OR 'tissue repair':ti,ab,kw OR reepithelial*:ti,ab,kw OR 're-epithelial*':ti,ab,kw)	29 July 2026	1,415

Database	Search strategy	Search date	Records retrieved (n)
Scopus	TITLE-ABS-KEY(becaplermin OR regranex OR "platelet-derived growth factor" OR "platelet-derived growth factor BB" OR "platelet derived growth factor BB" OR "platelet-derived growth factor-BB" OR "recombinant human platelet-derived growth factor" OR "recombinant human platelet derived growth factor" OR "recombinant platelet-derived growth factor" OR "recombinant platelet derived growth factor" OR "PDGF-BB" OR "PDGF BB" OR PDGFBB OR "rhPDGF-BB" OR "rhPDGF BB" OR rhPDGFBB OR "rPDGF-BB" OR "rPDGF BB" OR rPDGFBB OR rhPDGF OR rPDGF OR PDGF)<br>AND<br>TITLE-ABS-KEY("diabetes mellitus" OR hyperglycemia OR diabet* OR hyperglyc* OR "db/db" OR "db db" OR "ob/ob" OR "ob ob" OR streptozotocin OR STZ OR alloxan OR Akita OR "Zucker diabetic fatty" OR ZDF OR "Goto-Kakizaki" OR "high-fat diet" OR HFD)<br>AND<br>TITLE-ABS-KEY("wound healing" OR "skin ulcer" OR "diabetic foot" OR "surgical wound" OR wound* OR ulcer* OR "skin defect*" OR "cutaneous defect*" OR "skin injur*" OR "cutaneous injur*" OR "tissue repair" OR reepithelial* OR re-epithelial*)	29 July 2026	1,298
Web of Science Core Collection	TS=(becaplermin OR Regranex OR "platelet-derived growth factor" OR "platelet derived growth factor" OR "platelet-derived growth factor BB" OR "platelet derived growth factor BB" OR "platelet-derived growth factor-BB" OR "recombinant human platelet-derived growth factor" OR "recombinant human platelet derived growth factor" OR "recombinant platelet-derived growth factor" OR "recombinant platelet derived growth factor" OR "PDGF-BB" OR "PDGF BB" OR PDGFBB OR "rhPDGF-BB" OR "rhPDGF BB" OR rhPDGFBB OR "rPDGF-BB" OR "rPDGF BB" OR rPDGFBB OR rhPDGF OR rPDGF OR PDGF)<br>AND<br>TS=("diabetes mellitus" OR hyperglycemia OR diabet* OR hyperglyc* OR "db/db" OR "db db" OR "ob/ob" OR "ob ob" OR streptozotocin OR STZ OR alloxan OR Akita OR "Zucker diabetic fatty" OR ZDF OR "Goto-Kakizaki" OR "high-fat diet" OR HFD)<br>AND<br>TS=("wound healing" OR "skin ulcer" OR "diabetic foot" OR "surgical wound" OR wound* OR ulcer* OR "skin defect*" OR "cutaneous defect*" OR "skin injur*" OR "cutaneous injur*" OR "tissue repair" OR reepithelial* OR re-epithelial*)	29 July 2026	592
ScienceDirect (Search A)	(becaplermin OR Regranex OR "platelet derived growth factor BB" OR "PDGF BB" OR rhPDGF) AND (diabetes OR diabetic) AND (wound OR ulcer)	29 July 2026	54
ScienceDirect (Search B)	("recombinant human platelet derived growth factor" OR "recombinant platelet derived growth factor" OR rPDGF OR PDGF) AND (diabetes OR diabetic OR hyperglycemia) AND (wound OR ulcer)	29 July 2026	87
**Total**	**Records identified across all database searches before deduplication**		**3,844**

Note: ScienceDirect Searches A and B were conducted independently; their records were combined before deduplication. No restrictions were imposed regarding publication year or language, and no animal-study or preclinical study-design filters were applied. MeSH, Medical Subject Headings; TS, Topic; TITLE-ABS-KEY, title, abstract, and keywords.
