# Supplementary material for: Preclinical Efficacy of Recombinant Human Platelet-Derived Growth Factor-BB in Experimental Diabetic Wound Healing: A Systematic Review and Meta-Analysis of Animal Studies
Source: Pharmaceuticals (Basel). 2026 Sep 21;19(9):1497. doi: 10.3390/ph19091497 (PMC13610357; doi:10.3390/ph19091497)
Supplement: Supplementary file 1 [file pharmaceuticals-19-01497-s001.zip › Table_S4.pdf]

Table S4. Study-level context for the exploratory synthesis of directly attributable macroscopic wound-healing findings.

Study	Direction	Reported sample size for the eligible direct comparison	Experimental unit and independence	Quantitative context	Relevant risk-of-bias considerations
Deng et al., 2025	Favorable	Primary matched-carrier contrast: 12 mice (PDGF-BB-SH, n=6; WT-SH, n=6). Thirty-six mice across all six study arms.	One wound/mouse; parallel animal-level groups.	Percentage wound closure, days 10–12 (primary meta-analysis; Figure 4).	High risk for incomplete outcome data; sequence generation and allocation concealment unclear.
Saklani et al., 2024	Favorable	Eligible diabetic direct contrast: 8 mice (becaplermin, n=4; blank hydrogel, n=4).	One wound/mouse; parallel animal-level groups.	Not pooled.	Outcome denominators were not fully reconstructible; no confirmed non-independent wound-level analysis.
Ganesh et al., 2023	Favorable	Eligible direct contrast: 10 mice (Regranex, n=5; diabetic vehicle, n=5).	Four wounds/mouse; treatment assigned at the animal level and n reported as mice.	Not pooled.	No confirmed high-risk unit-of-analysis problem; sequence generation details and allocation concealment unclear.
Nguyen et al., 2018	Favorable	Becaplermin versus vehicle: day 7, 12 versus 11 mice; days 10/14, 9 versus 8 after scheduled tissue harvests.	One wound/mouse; parallel animal-level groups with prespecified terminal harvests.	Not pooled.	Outcome assessment blinded; no confirmed high-risk unit-of-analysis or incomplete-outcome concern.
McLaughlin et al., 2017	Favorable	Approximately 15 diabetic rats/experiment across three experiments (approximately 45 rats); exact arm-specific n not reported.	Four wounds/rat; treatments randomized within animals, including a duplicate wound; within-animal correlation not adequately addressed.	Not pooled.	High risk for other bias owing to non-independent wound observations; outcome denominators incompletely reconstructible.

Table S4. *Cont.*

Study	Direction	Reported sample size for the eligible direct comparison	Experimental unit and independence	Quantitative context	Relevant risk-of-bias considerations
Lee et al., 2016 [18]	Favorable	n=6 rats/group; 18 rats across the loaded scaffold, matched unloaded scaffold, and PLGA-only groups.	Two wounds/rat; wound-level observations were not adjusted for within-animal correlation.	Not pooled.	High risk for other bias owing to non-independent wound observations.
Lee et al., 2015 [19]	Favorable	n=6 rats/group; 18 rats across the loaded membrane, matched unloaded membrane, and gauze groups.	Two wounds/rat; wound-level observations were not adjusted for within-animal correlation.	Not pooled.	High risk for incomplete outcome data and for other bias owing to non-independent wound observations.
O'Brien et al., 2014 [20]	Favorable	Five diabetic pigs survived overall; the number contributing to the becaplermin comparison was not reported. Three wounds/treatment/pig.	Triplicate paired wounds within pigs; fixed positional assignment; within-animal correlation not adequately addressed.	Not pooled.	High risk for sequence generation, allocation concealment, and other bias; selective reporting and outcome denominators unclear.
Park et al., 2014 [21]	No clear direction	24 mice (rhPDGF-BB, n=12; vehicle, n=12).	Two wounds/mouse; both wounds received the same animal-level assignment.	Percentage wound closure, day 11 (primary meta-analysis; Figure 4).	Histopathology masked; no confirmed high-risk unit-of-analysis or incomplete-outcome concern.
Allen et al., 2014 [22]	Favorable	Eligible direct contrast: 16 diabetic mice (rhPDGF-BB, n=8; saline, n=8); 40 mice across all five study arms.	Two wounds/mouse; both wounds received the same animal-level assignment.	Not pooled.	No confirmed high-risk unit-of-analysis problem; sequence generation and allocation concealment unclear.

Table S4. *Cont.*

Study	Direction	Reported sample size for the eligible direct comparison	Experimental unit and independence	Quantitative context	Relevant risk-of-bias considerations
Dwivedi and Chaudhary, 2012 [23]	No clear direction	Eligible direct contrast: 12 diabetic rats (becaplermin, n=6; untreated diabetic, n=6); 24 rats analyzed overall.	One wound/rat; parallel animal-level groups.	Not pooled.	No confirmed high-risk unit-of-analysis or incomplete-outcome concern; allocation procedures unclear.
Cheng et al., 2011 [25]	Favorable	Eligible direct contrast: 18 mice across three experiments (becaplermin and placebo, n=3/group/experiment); 27 mice overall.	One wound/mouse; three independent experiments; animal-level n reported.	Not pooled.	No confirmed high-risk unit-of-analysis problem; sequence generation, allocation concealment, and assessor blinding unclear.
Tkalčević et al., 2007 [26]	No clear direction	Eligible direct contrast: 50 mice (rhPDGF-BB and vehicle, n=5/group at five terminal times); 75 mice overall.	One wound/mouse; independent prespecified terminal cohorts.	Not pooled.	Terminal sampling adequately accounted for; no confirmed high-risk unit-of-analysis or incomplete-outcome concern.
Cheng et al., 2007 [27]	Favorable	n=6–8 rats/group across three groups (18–24 enrolled); n=5–7 analyzed per outcome/time point.	Four wounds/rat; the level used for analysis could not be established definitively.	Not pooled.	High risk for incomplete outcome data; other bias unclear because the experimental unit was not definitively established.

Table S4. *Cont.*

Study	Direction	Reported sample size for the eligible direct comparison	Experimental unit and independence	Quantitative context	Relevant risk-of-bias considerations
Ševeljević-Jaran et al., 2006 [29]	No clear direction	n=6 rats/group in each imaging or histology experiment; exact unique total across separate cohorts not reported.	One wound/rat; separate imaging and terminal histology cohorts.	Not pooled.	Macroscopic assessment blinded; no confirmed high-risk unit-of-analysis or incomplete-outcome concern.
Chan et al., 2006 [30]	No clear direction	n=8 mice/group across separate wound-size and vehicle-control experiments; exact unique total not reported.	One wound/mouse; parallel experiments with incompletely reconstructible cohort overlap.	Not pooled.	Selective reporting unclear because of inconsistent histology assessment times; several design domains unclear.
Uhl et al., 2003 [31]	Favorable	24 hyperglycemic mice randomized; 17 analyzed (rhPDGF-BB, n=8; vehicle, n=9).	One ear wound/mouse; longitudinal animal-level analysis.	Time to complete closure (meta-analysis; Figure 6).	Attrition was reported and accounted for; no confirmed high-risk unit-of-analysis or incomplete-outcome concern.
Rodgers et al., 2003 [32]	Favorable	Eligible direct contrast: 10 mice (Regranex, n=5; placebo, n=5); 20 mice across all four diabetic groups.	One wound/mouse; parallel animal-level groups.	Not pooled: the complete-closure contrast was uninformative (0/5 vs. 0/5); other macroscopic findings were mapped.	Wound assessment blinded; no confirmed high-risk unit-of-analysis or incomplete-outcome concern.

Table S4. *Cont.*

Study	Direction	Reported sample size for the eligible direct comparison	Experimental unit and independence	Quantitative context	Relevant risk-of-bias considerations
Brown et al., 1997 [33]	Favorable	Eligible direct contrast: 24 mice (rhPDGF-BB and vehicle, n=6/group at days 5 and 10); 48 diabetic mice in the four-arm treatment experiment.	One wound/mouse; independent terminal cohorts.	Not pooled.	Terminal cohorts were planned; no confirmed high-risk unit-of-analysis or incomplete-outcome concern.
Kiritsy et al., 1995 [34]	Favorable	Eligible vehicle-controlled direct contrast: 28 mice (PDGF-BB, n=16; vehicle, n=12); 104 mice across both experiments.	One wound/mouse; parallel animal-level groups.	Time to complete closure (meta-analysis; Figure 6).	Wound assessment blinded; no confirmed high-risk unit-of-analysis or incomplete-outcome concern.
Brown et al., 1994 [35]	Favorable	n=8/group in the EGF experiment; pooled n=36/group in repeated TGF-α experiments; exact unique total across experiments not reported.	One wound/mouse; randomized parallel experiments repeated two to three times.	Not pooled.	Allocation concealment, intervention blinding, and outcome assessment were low risk; sequence-generation method incompletely reported.
Greenhalgh et al., 1990 [36]	Favorable	Primary meta-analysis: 77 mice across four component experiments (42 rhPDGF-BB-treated; 35 controls); exact unique total across the publication not reported.	One wound/mouse; multiple experiments and terminal cohorts were combined within publication before meta-analysis.	Percentage wound closure, day 10 (primary meta-analysis; Figure 4; dose-restricted sensitivity analysis, Figure 5).	Coded and blinded procedures; no confirmed high-risk unit-of-analysis or incomplete-outcome concern.

Note: The table summarizes the 22 study-level classifications contributing to the directly attributable macroscopic wound-healing synthesis. All mapped classifications were based on direct (D) contrasts; no formulation-level finding contributed to the 17/22 proportion. Sample sizes and experimental units are reproduced as reported and were not summed across studies because exact unique-animal totals could not be reconstructed reliably when reports used multiple wounds, repeated experiments, ranges, or terminal cohorts. Four studies had confirmed high-risk unit-of-analysis concerns involving non-independent wound observations [17–20]; all four were classified as favorable. No composite overall study-level risk-of-bias judgment was assigned. Sequence generation was unclear in 21 of the 22 studies and high risk in O'Brien et al. [20]; the final column highlights additional outcome-relevant concerns. The specific estimates included in the quantitative syntheses were not duplicated in the effect-direction map; other eligible macroscopic findings from the same publications remained eligible. Favorable indicates a study-level direction favoring the intervention; no clear direction indicates discordant findings or insufficient information to determine a direction. PDGF-BB-SH, platelet-derived growth factor-BB-functionalized sericin hydrogel; PLGA, poly(lactic-co-glycolic acid); rhPDGF-BB, recombinant human platelet-derived growth factor-BB; WT-SH, wild-type sericin hydrogel.
