# Supplementary material for: Preclinical Efficacy of Recombinant Human Platelet-Derived Growth Factor-BB in Experimental Diabetic Wound Healing: A Systematic Review and Meta-Analysis of Animal Studies
Source: Pharmaceuticals (Basel). 2026 Sep 21;19(9):1497. doi: 10.3390/ph19091497 (PMC13610357; doi:10.3390/ph19091497)
Supplement: Supplementary file 1 [file pharmaceuticals-19-01497-s001.zip › Table_S3.pdf]

Table S3. Robustness and influence diagnostics for the primary meta-analysis of percentage wound closure at days 10-12.

Panel A. Leave-one-study-out and sensitivity analyses

Analysis	Studies (k) / animals (n)	MD (percentage points)	95% CI	p-value	tau-squared	I-squared (%)	Interpretation
Primary analysis	3 / 113	12.94	-0.34 to 26.23	0.052	<0.0001	54.6	Three directly attributable study-level estimates.
Leave out Deng et al., 2025	2 / 101	6.91	-103.18 to 117.00	0.571	119.03	76.4	Also excludes the only study at high risk for incomplete outcome data.
Leave out Park et al., 2014	2 / 89	14.10	-13.26 to 41.45	0.096	0	0	Ad hoc Hartung-Knapp standard-error safeguard applied.
Leave out Greenhalgh et al., 1990	2 / 36	6.78	-104.58 to 118.14	0.581	118.02	74.7	Removes the publication contributing four internally pooled component experiments.
Greenhalgh dose-restricted analysis	3 / 91	8.71	-9.26 to 26.68	0.172	20.34	54.7	Greenhalgh contribution restricted to 10 micrograms/day for 5 days.
Restriction to mechanically splinted wounds	1 / 24	-3.60	-19.59 to 12.39	0.659	NA	NA	Unpooled estimate from Park et al., 2014; meta-analysis was not possible.
Exclude formulation-level (F) comparisons	NA	NA	NA	NA	NA	NA	Not applicable: all three primary contrasts were classified as directly attributable (D).
Exclude unresolved experimental-unit concerns	NA	NA	NA	NA	NA	NA	Not applicable: no contributing study had an unresolved experimental-unit problem.

Table S3. *Cont*.

Panel B. Influence diagnostics from the underlying REML model

Study	Externally studentized residual	DFFITS	Cook's distance	Covariance ratio	Deleted tau-squared	Deleted Q	Hat value	Weight (%)	DFBETAS	Default influence flag
Deng et al., 2025	0.5044	0.9265	8.4000	17.3279	119.0335	4.2353	0.3198	32.0	0.8714	Yes
Park et al., 2014	-2.0977	-0.5536	0.3065	1.0696	0	0.0008	0.0651	6.5	-0.5536	No
Greenhalgh et al., 1990	0.5119	0.7028	8.7708	17.7291	118.0202	3.9473	0.6151	61.5	0.8936	Yes

Note: Positive MD values favor the rhPDGF-BB-containing intervention. The primary and leave-one-study-out models used REML random effects with Hartung-Knapp inference. For leave-one-study-out models, the ad hoc standard-error safeguard (adhoc.hakn.ci = "se") was used because only two studies remained; this safeguard affected the Park-excluded interval, for which the unmodified Hartung-Knapp variance became arbitrarily small. I-squared values were calculated from Cochran's Q to remain consistent with the primary analysis. The splinted-only row reports the individual Park et al., 2014 estimate and was not meta-analyzed. Influence diagnostics were calculated from the underlying REML model and interpreted descriptively because k = 3. Default flags were used only as screening indicators; Deng et al., 2019 and Greenhalgh et al., 1990 were flagged primarily by Cook's distance. The Deng et al., 2019 comparison used a PDGF-BB-functionalized sericin hydrogel and a matched factor-free sericin hydrogel and therefore remained classified as D rather than F. CI, confidence interval; D, directly attributable comparison; DFBETAS, standardized change in the pooled coefficient after study deletion; DFFITS, standardized change in the fitted value after study deletion; F, formulation-level comparison; HK, Hartung-Knapp; MD, mean difference; NA, not applicable; REML, restricted maximum likelihood.
