# Supplementary material for: Preclinical Efficacy of Recombinant Human Platelet-Derived Growth Factor-BB in Experimental Diabetic Wound Healing: A Systematic Review and Meta-Analysis of Animal Studies
Source: Pharmaceuticals (Basel). 2026 Sep 21;19(9):1497. doi: 10.3390/ph19091497 (PMC13610357; doi:10.3390/ph19091497)
Supplement: Supplementary file 1 [file pharmaceuticals-19-01497-s001.zip › Table_S2.pdf]

Table S2. Study-level reporting of in vivo safety, tolerability, attrition, and systemic assessments.

Study	Study attribution	Safety/tolerability variables assessed	Safety-relevant follow-up	Reported adverse events or tolerability findings	Attrition and exclusions	Systemic laboratory or organ assessments	Attribution of the safety finding
Fang et al., 2025	F	Local H&E screening for ulceration, erosion, and localized fibroblast proliferation.	Local histology on day 18.	No clear local adverse-effect signal reported; mortality and other systemic effects NR.	No outcome attrition reported; low D8 risk.	NR.	Complete rhPDGF-BB hydrogel (F); the independent safety contribution of rhPDGF-BB was not isolated.
Deng et al., 2025	D/F	No eligible in vivo safety assessment; hemolysis testing was not performed in the wound model.	Study follow-up: 22 days; safety-specific follow-up NR.	NR.	Unexplained outcome-specific reductions or missing observations; high D8 risk.	NR.	Not assessable.
Saklani et al., 2024	D	OECD 402 dermal-toxicity observations, body weight, general health, and behavior.	Study follow-up: 14 days; safety-specific timing incompletely reported.	No adverse effects reported in the peptide-hydrogel groups.	Outcome denominators were incompletely reconstructible; unclear D8 risk.	Body weight and general clinical observations; laboratory and organ assessments NR.	Not specific to becaplermin; the reported toxicity assessment concerned the peptide-hydrogel groups.
Ganesh et al., 2023	D	No eligible in vivo safety assessment reported.	Study follow-up: 14 days; safety-specific follow-up NR.	NR.	No outcome attrition reported; low D8 risk.	NR.	Not assessable.

Table S2. *Cont.*

Study	Study attribution	Safety/tolerability variables assessed	Safety-relevant follow-up	Reported adverse events or tolerability findings	Attrition and exclusions	Systemic laboratory or organ assessments	Attribution of the safety finding
Alshoubaki et al., 2023	D/F	No eligible safety assessment in the wound experiment.	Study follow-up: 9 days; safety-specific follow-up NR.	NR.	Outcome-specific denominators varied (8-10 wounds/group); exact unique-animal total NR; high D8 risk.	NR.	Not assessable.
White et al., 2021	D/F	Daily wound inspection for signs of infection.	Through day 7.	No signs of wound infection reported; other treatment-related adverse effects NR.	Planned outcome-specific sample sizes varied; no unexplained attrition identified; low D8 risk.	NR.	Study-level monitoring across D/F interventions; rhPDGF-BB-specific safety was not reported separately.
Nguyen et al., 2018	D	No eligible in vivo safety assessment reported.	Study follow-up: 14 days; safety-specific follow-up NR.	NR.	Reduced later denominators reflected scheduled day-7 tissue harvests; low D8 risk.	NR.	Not assessable.
Gallant-Behm et al., 2018	D/F	Plasma ALT, AST, BUN, and creatinine; treatment distribution was also assessed.	Biochemistry/distribution: 48 h after the final dose; study follow-up: 10 days.	No liver or kidney dysfunction attributed to treatment.	No unexplained outcome attrition identified; low D8 risk.	ALT, AST, BUN, and creatinine; no organ histopathology reported.	Not clearly specific to rhPDGF-BB; systemic testing was reported at study/treatment level.

Table S2. *Cont.*

Study	Study attribution	Safety/tolerability variables assessed	Safety-relevant follow-up	Reported adverse events or tolerability findings	Attrition and exclusions	Systemic laboratory or organ assessments	Attribution of the safety finding
Ishihara et al., 2018	F	No eligible in vivo safety assessment reported.	Study follow-up: 10 days; safety-specific follow-up NR.	NR.	Outcome-specific denominators and overlap among cohorts were incompletely reported; high D8 risk.	NR.	Not assessable.
McLaughlin et al., 2017	D	Body weight, blood glucose, and local H&E assessment for cell death or structural pathology.	Clinical follow-up: 14 days; tissue assessments on days 1, 2, and 4.	No clear treatment-related toxicity reported; arm-specific safety results were limited.	Exact arm-specific denominators across repeated experiments NR; unclear D8 risk.	Body weight and blood glucose; systemic laboratory tests and organ histopathology NR.	D comparison, but treatment-specific adverse-event attribution was incompletely reported.
Lee et al., 2016	D	Local scaffold integration and inflammatory response.	Local tissue assessment on days 7 and 14.	No clear adverse local response attributed to treatment; systemic effects NR.	Outcome denominators were not fully reconstructible; unclear D8 risk.	NR.	Local matched-carrier comparison (D); systemic safety was not assessed.
Lee et al., 2015	D	Local inflammatory response to the implanted membrane.	Local tissue assessment on day 14.	No clear adverse local response attributed to treatment; systemic effects NR.	Unexplained outcome-specific reductions or missing observations; high D8 risk.	NR.	Local matched-carrier comparison (D); systemic safety was not assessed.

Table S2. *Cont.*

Study	Study attribution	Safety/tolerability variables assessed	Safety-relevant follow-up	Reported adverse events or tolerability findings	Attrition and exclusions	Systemic laboratory or organ assessments	Attribution of the safety finding
O'Brien et al., 2014	D	No eligible treatment-specific safety assessment reported.	Study follow-up: 21 days; becaplermin comparison assessed through day 14.	One of six diabetic pigs died; cause and relationship to treatment NR.	One pig died and five remained; number contributing to the becaplermin comparison NR; unclear D8 risk.	NR.	Not assessable for rhPDGF-BB.
Park et al., 2014	D	Daily body weight, appetite, pain/general health, wound infection, and exudate monitoring.	Daily through day 11.	No treatment-related clinical toxicity reported.	No outcome attrition reported; low D8 risk.	Body weight and clinical observations; laboratory and organ assessments NR.	rhPDGF-BB versus factor-free vehicle (D).
Allen et al., 2014	D/F	No eligible in vivo safety assessment reported.	Study follow-up: 28 days or until closure; safety-specific follow-up NR.	NR.	No outcome attrition reported; low D8 risk.	NR.	Not assessable.
Dwivedi and Chaudhary, 2012	D	No eligible in vivo safety assessment reported.	Study follow-up: 14 days; safety-specific follow-up NR.	NR.	Twenty-two of 24 alloxan-treated rats were available and 18 were randomly selected; no outcome attrition reported; low D8 risk.	NR.	Not assessable.

Table S2. *Cont.*

Study	Study attribution	Safety/tolerability variables assessed	Safety-relevant follow-up	Reported adverse events or tolerability findings	Attrition and exclusions	Systemic laboratory or organ assessments	Attribution of the safety finding
Berdal et al., 2011	F	Clinical signs and wound cultures for infection.	Closure follow-up to day 17; observation to day 45.	Wound-infection incidence was reported by group; other adverse effects NR.	Outcome-related exclusions or reductions were incompletely addressed; high D8 risk.	NR.	Complete rhPDGF-BB plus rhIGF-1 formulation (F); rhPDGF-BB-specific safety was not isolated.
Cheng et al., 2011	D	No eligible in vivo safety assessment reported.	Study follow-up: 18 days; safety-specific follow-up NR.	NR.	No unexplained outcome attrition identified; low D8 risk.	NR.	Not assessable.
Tkalčević et al., 2007	D	No eligible in vivo safety assessment reported.	Study follow-up: 24 days; safety-specific follow-up NR.	NR.	Prespecified terminal cohorts were accounted for; low D8 risk.	NR.	Not assessable.
Cheng et al., 2007	D	Body weight and blood glucose monitoring.	Through day 14.	Treatment-related adverse effects NR.	Six to eight rats/group enrolled and five to seven analyzed per outcome/time point; high D8 risk.	Body weight and blood glucose; systemic laboratory tests and organ histopathology NR.	D comparison, but adverse-event attribution was not reported.

Table S2. *Cont.*

Study	Study attribution	Safety/tolerability variables assessed	Safety-relevant follow-up	Reported adverse events or tolerability findings	Attrition and exclusions	Systemic laboratory or organ assessments	Attribution of the safety finding
Zhao et al., 2006	D	No eligible safety assessment in the db/db wound experiment.	Study follow-up: 11 days; safety-specific follow-up NR.	NR.	Paired wounds were retained; no outcome attrition reported; low D8 risk.	NR.	Not assessable.
Ševeljević-Jaran et al., 2006	D	No eligible in vivo safety assessment reported.	Study follow-up: 8 days; safety-specific follow-up NR.	NR.	Separate imaging and terminal histology cohorts were planned; low D8 risk.	NR.	Not assessable.
Chan et al., 2006	D	No eligible in vivo safety assessment reported.	Study follow-up: up to 24 days; safety-specific follow-up NR.	NR.	No outcome attrition reported; exact unique total across experiments NR; low D8 risk.	NR.	Not assessable.
Uhl et al., 2003	D	Daily clinical checks and documentation of reasons for loss from analysis.	Daily until closure; assessments through day 24.	Losses were associated with severe hyperglycemia, weight loss/exsiccosis, or wound perforation; treatment relationship NR.	Twenty-four hyperglycemic mice randomized and 17 analyzed; reasons were reported; low D8 risk.	No laboratory or organ assessment reported.	D study, but the reported losses were not attributed specifically to rhPDGF-BB.

Table S2. *Cont.*

Study	Study attribution	Safety/tolerability variables assessed	Safety-relevant follow-up	Reported adverse events or tolerability findings	Attrition and exclusions	Systemic laboratory or organ assessments	Attribution of the safety finding
Rodgers et al., 2003	D	No eligible in vivo safety assessment reported.	Study follow-up: 25 days; safety-specific follow-up NR.	NR.	No outcome attrition reported; low D8 risk.	NR.	Not assessable.
Brown et al., 1997	D/F	No eligible in vivo safety assessment reported.	Study follow-up: 28 days; treatment cohorts assessed on days 5 and 10.	NR.	Prespecified terminal cohorts were accounted for; low D8 risk.	NR.	Not assessable.
Kiritsy et al., 1995	D/F	General treatment tolerance and mortality.	Twice weekly until complete healing, up to day 63.	Late mortality occurred equally across groups and was attributed by the authors to age and frequent anesthesia.	Mortality was reported and explained; low D8 risk.	No laboratory or organ assessment reported.	D/F study; equal mortality across groups did not identify an rhPDGF-BB-specific signal.

Table S2. *Cont.*

Study	Study attribution	Safety/tolerability variables assessed	Safety-relevant follow-up	Reported adverse events or tolerability findings	Attrition and exclusions	Systemic laboratory or organ assessments	Attribution of the safety finding
Brown et al., 1994	D/F	No eligible in vivo safety assessment reported.	Study follow-up: 21 days; safety-specific follow-up NR.	NR.	No unexplained outcome attrition identified; exact unique total across repeated experiments NR; low D8 risk.	NR.	Not assessable.
Greenhalgh et al., 1990	D/F	Body weight, food intake, general tolerance, and gross procedural observations.	Wounds checked 3-5 times weekly for up to 42 days; one chronic wound followed to day 90.	No gross procedural or treatment-tolerance problems reported.	No unexplained outcome attrition identified; low D8 risk.	Body weight and food intake; laboratory tests and organ histopathology NR.	D/F study; reporting did not isolate a formulation- or rhPDGF-BB-specific safety effect.

Note: Study attribution describes the eligible intervention-comparator structures in each publication and does not imply that safety outcomes were reported separately for every contrast. D denotes a directly attributable comparison and F denotes a formulation-level comparison; the last column specifies the attribution permitted by the reported safety observation. D8 refers to the SYRCLE incomplete-outcome-data domain. Study follow-up is shown when a distinct safety-specific observation period was not reported. NR and 'no eligible safety assessment' indicate absent or insufficient reporting and must not be interpreted as evidence that adverse effects were absent. ALT, alanine aminotransferase; AST, aspartate aminotransferase; BUN, blood urea nitrogen; H&E, hematoxylin and eosin; rhIGF-1, recombinant human insulin-like growth factor 1; rhPDGF-BB, recombinant human platelet-derived growth factor-BB.
