# Supplementary material for: Preclinical Efficacy of Recombinant Human Platelet-Derived Growth Factor-BB in Experimental Diabetic Wound Healing: A Systematic Review and Meta-Analysis of Animal Studies
Source: Pharmaceuticals (Basel). 2026 Sep 21;19(9):1497. doi: 10.3390/ph19091497 (PMC13610357; doi:10.3390/ph19091497)
Supplement: Supplementary file 1 [file pharmaceuticals-19-01497-s001.zip › Table_S1.pdf]

Table S1. Detailed experimental groups and sample sizes.

Study	Experiment/ comparison set	Experimental group/arm	Treatment regimen	n reported	Unit of n	Eligible comparison(s)	Notes
Fang et al., 2025	Main wound-healing experiment	Normal control	No hydrogel; transparent dressing	3	mice	Contextual nondiabetic reference	—
Fang et al., 2025	Main wound-healing experiment	Diabetic untreated	No hydrogel; transparent dressing	3	mice	Comparator for composite hydrogel	—
Fang et al., 2025	Main wound-healing experiment	Diabetic rhPDGF-BB hydrogel	100 μL PEI–polypyrrole–F127 hydrogel containing 0.5 μg rhPDGF-BB every other day through day 10	3	mice	vs diabetic untreated	One wound/mouse; formulation-level attribution (F)
Deng et al., 2025	Six-arm randomized wound-healing experiment	Null	Saline	6	mice	Reference for all active arms	—
Deng et al., 2025	Six-arm randomized wound-healing experiment	WT-SH	Wild-type silk-sericin hydrogel disc	6	mice	Matched carrier comparator for GF hydrogels	—
Deng et al., 2025	Six-arm randomized wound-healing experiment	EGF-SH	EGF-functionalized sericin hydrogel	6	mice	Active non-PDGF comparator	—
Deng et al., 2025	Six-arm randomized wound-healing experiment	PDGF-BB-SH	PDGF-BB-functionalized sericin hydrogel; approximately 1.18 μg/g	6	mice	vs WT-SH and Null	Direct PDGF comparison (D)
Deng et al., 2025	Six-arm randomized wound-healing experiment	EGF/PDGF-BB-SH	Dual EGF/PDGF-BB sericin hydrogel	6	mice	vs WT-SH, single-GF hydrogels, and Null	Multicomponent formulation (F)
Deng et al., 2025	Six-arm randomized wound-healing experiment	Commercial GF standards	Commercial EGF + PDGF-BB solution	6	mice	Active reference for dual-GF hydrogel	Exact commercial GF doses NR
Saklani et al., 2024	Normal wound model	Blank hydrogel	Na-CMC hydrogel, 1 mg/wound/day for 4 days	4	mice	Vehicle comparator	—
Saklani et al., 2024	Normal wound model	Becaplermin standard	Becaplermin gel, 1 mg/wound/day for 4 days; active concentration NR	4	mice	Active reference comparator	—
Saklani et al., 2024	Normal wound model	IKVAV hydrogel	IKVAV peptide hydrogel, 1 mg/wound/day for 4 days	4	mice	vs blank and becaplermin	—
Saklani et al., 2024	Normal wound model	DYVRLAI hydrogel	DYVRLAI peptide hydrogel, 1 mg/wound/day for 4 days	4	mice	vs blank and becaplermin	—
Saklani et al., 2024	Normal wound model	CDYVRLAI hydrogel	CDYVRLAI peptide hydrogel, 1 mg/wound/day for 4 days	4	mice	vs blank and becaplermin	—
Saklani et al., 2024	Normal wound model	PGPIKVAV hydrogel	PGPIKVAV peptide hydrogel, 1 mg/wound/day for 4 days	4	mice	vs blank and becaplermin	—
Saklani et al., 2024	Normal wound model	Ac-PGPIKVAV hydrogel	Ac-PGPIKVAV peptide hydrogel, 1 mg/wound/day for 4 days	4	mice	vs blank and becaplermin	—
Saklani et al., 2024	Alloxan-diabetic wound model	Blank hydrogel	Na-CMC hydrogel, 1 mg/wound/day for 4 days	4	mice	Vehicle comparator	—
Saklani et al., 2024	Alloxan-diabetic wound model	Becaplermin standard	Becaplermin gel, 1 mg/wound/day for 4 days; active concentration NR	4	mice	Active reference comparator	—
Saklani et al., 2024	Alloxan-diabetic wound model	IKVAV hydrogel	IKVAV peptide hydrogel, 1 mg/wound/day for 4 days	4	mice	vs blank and becaplermin	—
Saklani et al., 2024	Alloxan-diabetic wound model	DYVRLAI hydrogel	DYVRLAI peptide hydrogel, 1 mg/wound/day for 4 days	4	mice	vs blank and becaplermin	—
Saklani et al., 2024	Alloxan-diabetic wound model	CDYVRLAI hydrogel	CDYVRLAI peptide hydrogel, 1 mg/wound/day for 4 days	4	mice	vs blank and becaplermin	—
Saklani et al., 2024	Alloxan-diabetic wound model	PGPIKVAV hydrogel	PGPIKVAV peptide hydrogel, 1 mg/wound/day for 4 days	4	mice	vs blank and becaplermin	—
Saklani et al., 2024	Alloxan-diabetic wound model	Ac-PGPIKVAV hydrogel	Ac-PGPIKVAV peptide hydrogel, 1 mg/wound/day for 4 days	4	mice	vs blank and becaplermin	—
Ganesh et al., 2023	Six-arm randomized experiment	CW	Normoglycemic + vehicle i.p. daily for 14 days	5	mice	Contextual nondiabetic reference	—
Ganesh et al., 2023	Six-arm randomized experiment	DW	Diabetic + vehicle i.p. daily for 14 days	5	mice	Reference for diabetic active arms	—
Ganesh et al., 2023	Six-arm randomized experiment	DW + PTS5	Pterostilbene 5 mg/kg i.p. daily for 14 days	5	mice	vs DW and Regranex	—
Ganesh et al., 2023	Six-arm randomized experiment	DW + PTS10	Pterostilbene 10 mg/kg i.p. daily for 14 days	5	mice	vs DW and Regranex	—
Ganesh et al., 2023	Six-arm randomized experiment	CW + PTS10	Normoglycemic + pterostilbene 10 mg/kg i.p. daily for 14 days	5	mice	Contextual active control	—
Ganesh et al., 2023	Six-arm randomized experiment	DW + Reg	Diabetic + Regranex 0.01% topically once daily for 14 days; amount NR	5	mice	vs DW and pterostilbene arms	Four wounds/mouse
Alshoubaki et al., 2023	Day-9 engineered-PDGF comparison	Saline	10 μL topical saline on day 1	10	wounds	Vehicle comparator	Two wounds/mouse; unique mice NR
Alshoubaki et al., 2023	Day-9 engineered-PDGF comparison	WT PDGF-BB	0.5 μg wild-type human rhPDGF-BB in 10 μL on day 1	8	wounds	vs saline and fusion protein	Two wounds/mouse; unique mice NR
Alshoubaki et al., 2023	Day-9 engineered-PDGF comparison	AREG26–38–PDGF-BB	0.5 μg equimolar engineered fusion in 10 μL on day 1	10	wounds	vs saline and WT PDGF-BB	Two wounds/mouse; unique mice NR

Table S1. Cont.

Study	Experiment/ comparison set	Experimental group/arm	Treatment regimen	n reported	Unit of n	Eligible comparison(s)	Notes
White et al., 2021	Single-GF and multi-GF fibrin experiments	NOR fibrin control	Nondiabetic NOR + fibrin vehicle	3–20	mice across outcomes	Contextual nondiabetic reference	—
White et al., 2021	Single-GF and multi-GF fibrin experiments	NOD fibrin control	Diabetic NOD + fibrin vehicle	3–20	mice across outcomes	Reference for GF arms	—
White et al., 2021	Single-GF and multi-GF fibrin experiments	VEGF-A165	200 ng wild-type VEGF-A165/wound in fibrin	3–20	mice across outcomes	Active non-PDGF arm	—
White et al., 2021	Single-GF and multi-GF fibrin experiments	VEGF-A165–PlGF	200 ng engineered VEGF-A165–PlGF/wound in fibrin	3–20	mice across outcomes	Active non-PDGF arm	—
White et al., 2021	Single-GF and multi-GF fibrin experiments	PDGF-BB	200 ng wild-type human PDGF-BB/wound in fibrin	3–20	mice across outcomes	vs NOD fibrin and engineered PDGF	Direct PDGF arm (D)
White et al., 2021	Single-GF and multi-GF fibrin experiments	PDGF-BB–PlGF	200 ng engineered PDGF-BB–PlGF/wound in fibrin	3–20	mice across outcomes	vs NOD fibrin and WT PDGF	Direct PDGF arm (D)
White et al., 2021	Single-GF and multi-GF fibrin experiments	HB-EGF	200 ng wild-type HB-EGF/wound in fibrin	3–20	mice across outcomes	Active non-PDGF arm	—
White et al., 2021	Single-GF and multi-GF fibrin experiments	HB-EGF–PlGF	200 ng engineered HB-EGF–PlGF/wound in fibrin	3–20	mice across outcomes	Active non-PDGF arm	—
White et al., 2021	Single-GF and multi-GF fibrin experiments	VEGF + PDGF	200 ng each wild-type VEGF-A165 and PDGF-BB/wound	3–20	mice across outcomes	vs fibrin and engineered pair	Two-GF formulation (F)
White et al., 2021	Single-GF and multi-GF fibrin experiments	VEGF–PlGF + PDGF–PlGF	200 ng each engineered VEGF-A165–PlGF and PDGF-BB–PlGF/wound	3–20	mice across outcomes	vs fibrin and WT pair	Two-GF formulation (F)
White et al., 2021	Single-GF and multi-GF fibrin experiments	WT triple GF	200 ng each WT VEGF-A165, PDGF-BB, and HB-EGF/wound	3–20	mice across outcomes	vs fibrin and engineered triple	Three-GF formulation (F)
White et al., 2021	Single-GF and multi-GF fibrin experiments	Engineered triple GF	200 ng each VEGF-A165–PlGF, PDGF-BB–PlGF, and HB-EGF–PlGF/wound	3–20	mice across outcomes	vs fibrin and WT triple	Three-GF formulation (F); exact arm-specific n NR
Nguyen et al., 2018	Becaplermin head-to-head experiment	Vehicle	50 μL topical vehicle once daily, days 1–14	11 (day 7); 8 (days 10/14)	mice	Reference for R-ND-336 and becaplermin	—
Nguyen et al., 2018	Becaplermin head-to-head experiment	R-ND-336	50 μg in 50 μL/wound/day, days 1–14	11 (day 7); 7 (days 10/14)	mice	vs vehicle and becaplermin	—
Nguyen et al., 2018	Becaplermin head-to-head experiment	Becaplermin	Regranex 0.01%, 5 μg in 50 μL/wound/day, days 1–14	12 (day 7); 9 (days 10/14)	mice	vs vehicle and R-ND-336	Three mice/group scheduled for day-7 tissue harvest

Table S1. Cont.

Study	Experiment/ comparison set	Experimental group/arm	Treatment regimen	n reported	Unit of n	Eligible comparison(s)	Notes
Gallant-Behm et al., 2018	Dose-screening experiment	PBS	Four 25-μL periwound intradermal injections on days 0, 2, 4, and 8	10	mice	Reference for active arms	—
Gallant-Behm et al., 2018	Dose-screening experiment	rhVEGF165	1 μg/wound on days 0, 2, 4, and 8	10	mice	Active non-PDGF comparator	—
Gallant-Behm et al., 2018	Dose-screening experiment	rhPDGF-BB	2 μg/wound on days 0, 2, 4, and 8	10	mice	vs PBS and other active arms	—
Gallant-Behm et al., 2018	Dose-screening experiment	MRG-110 10 nmol	10 nmol/wound on days 0, 2, 4, and 8	10	mice	vs PBS and rhPDGF-BB	—
Gallant-Behm et al., 2018	Dose-screening experiment	MRG-110 30 nmol	30 nmol/wound on days 0, 2, 4, and 8	10	mice	vs PBS and rhPDGF-BB	—
Gallant-Behm et al., 2018	Dose-screening experiment	MRG-110 100 nmol	100 nmol/wound on days 0, 2, 4, and 8	10	mice	vs PBS and rhPDGF-BB	—
Gallant-Behm et al., 2018	Dose-screening experiment	AntimiR #2, 10/30/100 nmol	Control antimiR at each dose on days 0, 2, 4, and 8	10/dose	mice	Sequence-control arms	—
Gallant-Behm et al., 2018	rhPDGF-BB combination experiment	PBS	Periwound intradermal injections on days 0, 2, 4, and 8	8–10	mice	Reference for combination experiment	—
Gallant-Behm et al., 2018	rhPDGF-BB combination experiment	rhPDGF-BB	2 μg/wound on days 0, 2, 4, and 8	8–10	mice	vs PBS and combinations	—
Gallant-Behm et al., 2018	rhPDGF-BB combination experiment	MRG-110	30 nmol/wound on days 0, 2, 4, and 8	8–10	mice	vs PBS and combination	—
Gallant-Behm et al., 2018	rhPDGF-BB combination experiment	MRG-110 92 nmol	92 nmol/wound on days 0, 2, 4, and 8	8–10	mice	vs PBS	—
Gallant-Behm et al., 2018	rhPDGF-BB combination experiment	rhPDGF-BB + MRG-110	2 μg rhPDGF-BB + 30 nmol MRG-110/wound on days 0, 2, 4, and 8	8–10	mice	vs monotherapies and PBS	—
Ishihara et al., 2018	Four-arm wound-healing experiment	Fibrin	80 μL fibrin/wound, single application	NR	wounds	Vehicle comparator	—
Ishihara et al., 2018	Four-arm wound-healing experiment	LAMA3 peptide fibrin	Laminin peptide in 80 μL fibrin/wound	NR	wounds	Carrier/peptide comparator	—
Ishihara et al., 2018	Four-arm wound-healing experiment	GF fibrin	50 ng PDGF-BB + 100 ng VEGF-A165 in 80 μL fibrin/wound	NR	wounds	vs fibrin and peptide-GF formulation	—
Ishihara et al., 2018	Four-arm wound-healing experiment	LAMA3 peptide + GF fibrin	Laminin peptide + 50 ng PDGF-BB + 100 ng VEGF-A165 in 80 μL fibrin/wound	NR	wounds	vs peptide alone and GF fibrin	27 mice in main model; four wounds/mouse; allocation by wound/time NR
McLaughlin et al., 2017	Three repeated wound-randomized experiments	Vehicle	0.05 mL saline/cream vehicle topically once daily through day 14	NR	wounds	Reference for naltrexone and Regranex	—
McLaughlin et al., 2017	Three repeated wound-randomized experiments	Naltrexone	0.03% naltrexone cream, 0.05 mL/wound/day through day 14	NR	wounds	vs vehicle and Regranex	—
McLaughlin et al., 2017	Three repeated wound-randomized experiments	Regranex	0.05 mL becaplermin gel/wound/day through day 14; active concentration NR	NR	wounds	vs vehicle and naltrexone	—
McLaughlin et al., 2017	Three repeated wound-randomized experiments	Duplicate randomized wound	Fourth wound repeated one of the assigned treatments	NR	wounds	Within-animal replicate	Approximately 15 diabetic rats/experiment; four wounds/rat; exact arm counts NR

Table S1. Cont.

Study	Experiment/ comparison set	Experimental group/arm	Treatment regimen	n reported	Unit of n	Eligible comparison(s)	Notes
Lee et al., 2016	Three-arm scaffold experiment	rhPDGF-BB/PLGA–collagen	Single rhPDGF-BB-eluting PLGA–collagen scaffold	6	rats	vs matched scaffold and PLGA only	Two wounds/rat; per-wound PDGF dose NR
Lee et al., 2016	Three-arm scaffold experiment	PLGA–collagen	Single unloaded PLGA–collagen scaffold	6	rats	Matched formulation-minus-factor comparator	—
Lee et al., 2016	Three-arm scaffold experiment	PLGA	Single PLGA-only scaffold	6	rats	Material comparator	—
Lee et al., 2015	Three-arm membrane experiment	rhPDGF-BB/PLGA membrane	Single rhPDGF-BB-loaded PLGA nanofibrous membrane	6	rats	vs PLGA membrane and gauze	Two wounds/rat; per-wound PDGF dose NR
Lee et al., 2015	Three-arm membrane experiment	PLGA membrane	Single unloaded PLGA nanofibrous membrane	6	rats	Matched formulation-minus-factor comparator	—
Lee et al., 2015	Three-arm membrane experiment	Conventional gauze	Gauze dressing	6	rats	Standard dressing comparator	—
O'Brien et al., 2014	Paired F-5/becaplermin experiment	CMC	5% CMC vehicle, single topical application	3/pig	wounds	Vehicle comparator	—
O'Brien et al., 2014	Paired F-5/becaplermin experiment	F-5	Hsp90α F-5 peptide 45 μM, single topical application	3/pig	wounds	vs CMC and becaplermin	—
O'Brien et al., 2014	Paired F-5/becaplermin experiment	Becaplermin	Becaplermin gel as prescribed, single topical application; exact dose NR	3/pig	wounds	vs CMC and F-5	Triplicate wounds/treatment/pig; number of pigs in this comparison NR
Park et al., 2014	Two-arm randomized splinted-wound experiment	Vehicle	30 μL 5% PEG/PBS/wound immediately and daily for 10 days	12	mice	Vehicle comparator	—
Park et al., 2014	Two-arm randomized splinted-wound experiment	rhPDGF-BB	3 μg human rhPDGF-BB in 30 μL/wound immediately and daily for 10 days	12	mice	vs vehicle	Two wounds/mouse; both wounds received same assignment
Allen et al., 2014	Five-arm combination experiment	WT saline	Wild-type mouse + saline	8	mice	Contextual nondiabetic reference	—
Allen et al., 2014	Five-arm combination experiment	Diabetic saline	db/db mouse + saline	8	mice	Reference for diabetic active arms	—
Allen et al., 2014	Five-arm combination experiment	Diabetic AMD3100	AMD3100 10 mg/kg i.p. daily from day 3 until closure	8	mice	vs diabetic saline and combination	—
Allen et al., 2014	Five-arm combination experiment	Diabetic rhPDGF-BB	Regranex 0.01%, 2 μg/wound/day from day 3 until closure	8	mice	vs diabetic saline and combination	—
Allen et al., 2014	Five-arm combination experiment	Diabetic AMD3100 + rhPDGF-BB	AMD3100 10 mg/kg i.p. + Regranex 2 μg/wound/day from day 3 until closure	8	mice	vs both monotherapies and diabetic saline	Two wounds/mouse
Dwivedi & Chaudhary, 2012	Four-arm wound-healing experiment	Normal control	Saline cleansing twice daily; no active treatment	6	rats	Contextual nondiabetic reference	—
Dwivedi & Chaudhary, 2012	Four-arm wound-healing experiment	Diabetic untreated	Saline cleansing twice daily; no active treatment	6	rats	Diabetic reference	—
Dwivedi & Chaudhary, 2012	Four-arm wound-healing experiment	Ampucare	Ampucare 0.5 mL topically twice daily for 14 days	6	rats	vs diabetic untreated and becaplermin	—
Dwivedi & Chaudhary, 2012	Four-arm wound-healing experiment	Becaplermin	Becaplermin gel 50 mg topically twice daily for 14 days; active concentration NR	6	rats	vs diabetic untreated and Ampucare	—

Table S1. Cont.

Study	Experiment/ comparison set	Experimental group/arm	Treatment regimen	n reported	Unit of n	Eligible comparison(s)	Notes
Berdal et al., 2011	Eight-group dose-schedule experiment	AG single dose	Aminated β-1,3-D-glucan, one topical dose	10	mice	vs PDGF-BB + IGF-1 and placebo	—
Berdal et al., 2011	Eight-group dose-schedule experiment	AG every 10th day	Aminated β-1,3-D-glucan, one dose every 10th day	7	mice	vs PDGF-BB + IGF-1 and placebo	—
Berdal et al., 2011	Eight-group dose-schedule experiment	AG ×5	Aminated β-1,3-D-glucan once daily for 5 consecutive days	11	mice	vs PDGF-BB + IGF-1 and placebo	—
Berdal et al., 2011	Eight-group dose-schedule experiment	AG ≥15 doses	Aminated β-1,3-D-glucan in cycles until closure; at least 15 doses	11	mice	vs PDGF-BB + IGF-1 and placebo	—
Berdal et al., 2011	Eight-group dose-schedule experiment	rhPDGF-BB + rhIGF-1	1 µg of each GF in 100 µL HSA/saline once daily on days 0–4	12	mice	vs AG schedules and diabetic placebo	Multicomponent GF active control (F)
Berdal et al., 2011	Eight-group dose-schedule experiment	Placebo + insulin	Saline topically once daily for 5 days + subcutaneous insulin implant	14	mice	Diabetic metabolic-treatment control	—
Berdal et al., 2011	Eight-group dose-schedule experiment	Diabetic placebo	Saline topically once daily for 5 days	10	mice	Diabetic placebo reference	—
Berdal et al., 2011	Eight-group dose-schedule experiment	Nondiabetic placebo	db/+ mouse + saline topically once daily for 5 days	12	mice	Contextual nondiabetic reference	—
Cheng et al., 2011	Three-arm active-reference experiment	Placebo	100 µL CMC gel, single topical application (5% in Methods; 10% in Figures 5–6)	3/experiment	mice	Vehicle comparator	Internal CMC-concentration discrepancy
Cheng et al., 2011	Three-arm active-reference experiment	F-5	Hsp90α F-5 peptide (~1 mM) in 100 µL CMC gel, single topical application (5% in Methods; 10% in Figures 5–6)	3/experiment	mice	vs placebo and becaplermin	—
Cheng et al., 2011	Three-arm active-reference experiment	Becaplermin	100 µL Regranex 0.01%, single topical application; active mass NR	3/experiment	mice	vs placebo and F-5	Three independent experiments; 27 mice total
Tkalčević et al., 2007	Three-arm terminal-cohort experiment	Vehicle	50 µL PBS/0.1% mouse serum albumin immediately and daily through day 18	5/time point	mice	Vehicle comparator	—
Tkalčević et al., 2007	Three-arm terminal-cohort experiment	rhPDGF-BB	1 µg in 50 µL/wound immediately and daily through day 18	5/time point	mice	vs vehicle and PL14736	—
Tkalčević et al., 2007	Three-arm terminal-cohort experiment	PL14736	Synthetic 15-amino-acid peptide, 100 µg in 50 µL/wound immediately and daily through day 18	5/time point	mice	Mechanistic active comparator	Five terminal times (days 6, 9, 12, 18, 24); 75 mice total
Cheng et al., 2007	Three-arm diabetic wound experiment	Untreated	No topical gel	6–8	rats/group	Untreated comparator	—
Cheng et al., 2007	Three-arm diabetic wound experiment	Vehicle	3.5% CMC/saline topically on days 1–14	6–8	rats/group	Vehicle comparator	—
Cheng et al., 2007	Three-arm diabetic wound experiment	rhPDGF-BB	Regranex 0.01% at 7.0 µg/cm² topically on days 1–14	6–8	rats/group	vs untreated and vehicle	Four wounds/rat; 5–7 analyzed/time point
Zhao et al., 2006	Paired-wound active-reference experiment	SBD.4A wound	SBD.4A 2 mg in 10 mg 2% CMC/wound on day 0 and every second day through day 10	6	mice	vs contralateral CMC wound	—
Zhao et al., 2006	Paired-wound active-reference experiment	SBD.4A contralateral control	10 mg 2% CMC/wound on the same schedule	6	mice	Paired vehicle comparator	—
Zhao et al., 2006	Paired-wound active-reference experiment	Becaplermin wound	Regranex 10 mg gel/wound on day 0 and every second day through day 10	6	mice	vs contralateral CMC wound	—
Zhao et al., 2006	Paired-wound active-reference experiment	Becaplermin contralateral control	10 mg 2% CMC/wound on the same schedule	6	mice	Paired vehicle comparator	Two wounds/mouse; 12 mice total

Table S1. Cont.

Study	Experiment/ comparison set	Experimental group/arm	Treatment regimen	n reported	Unit of n	Eligible comparison(s)	Notes
Ševeljević-Jaran et al., 2006	Dose-ranging active-reference experiment	Untreated	No topical treatment	6	rats/group/cohort	Untreated comparator	—
Ševeljević-Jaran et al., 2006	Dose-ranging active-reference experiment	Carbopol vehicle	0.05 g carbopol gel/wound/day	6	rats/group/cohort	Vehicle comparator	—
Ševeljević-Jaran et al., 2006	Dose-ranging active-reference experiment	PL14736 10 μg	10 μg in 0.05 g gel/wound/day	6	rats/group/cohort	vs vehicle and Regranex	—
Ševeljević-Jaran et al., 2006	Dose-ranging active-reference experiment	PL14736 100 μg	100 μg in 0.05 g gel/wound/day	6	rats/group/cohort	vs vehicle and Regranex	—
Ševeljević-Jaran et al., 2006	Dose-ranging active-reference experiment	PL14736 500 μg	500 μg in 0.05 g gel/wound/day	6	rats/group/cohort	vs vehicle and Regranex	—
Ševeljević-Jaran et al., 2006	Dose-ranging active-reference experiment	PL14736 1000 μg	1000 μg in 0.05 g gel/wound/day	6	rats/group/cohort	vs vehicle and Regranex	—
Ševeljević-Jaran et al., 2006	Dose-ranging active-reference experiment	Regranex	20 μg becaplermin in 0.2 g 0.01% gel/wound/day	6	rats/group/cohort	vs untreated, vehicle, and PL14736 doses	Daily ×5 for imaging; separate daily ×3 and ×7 histology cohorts
Chan et al., 2006	Main and vehicle-control experiments	Wild-type control	Untreated nondiabetic control beneath Opsite	8	mice	Contextual nondiabetic reference	—
Chan et al., 2006	Main and vehicle-control experiments	Diabetic vehicle controls	Saline or 5% PEG in separate vehicle experiments, 0.2 mL/wound/day on days 1–5	8	mice	Vehicle comparator	Saline and PEG were evaluated as separate controls
Chan et al., 2006	Main and vehicle-control experiments	Diabetic Regranex	10 μg rhPDGF-BB in 0.2 mL/wound/day on days 1–5	8	mice	vs diabetic vehicle	Related experiments used 0.6-, 1.0-, and 1.5-cm^2 wounds
Uhl et al., 2003	Randomized ear-wound experiment	Carrier	3 μL sodium acetate/0.25% albumin, single application	9	mice analyzed	Vehicle comparator	—
Uhl et al., 2003	Randomized ear-wound experiment	rhPDGF-BB	3 μg human rhPDGF-BB in 3 μL, single application	8	mice analyzed	vs carrier	24 hyperglycemic mice randomized; final analyzed n shown
Rodgers et al., 2003	Four-arm topical experiment	Placebo	10% CMC vehicle topically once daily on days 0–4	5	mice	Placebo comparator	—
Rodgers et al., 2003	Four-arm topical experiment	Angiotensin-(1–7)	100 μg/wound/day topically on days 0–4	5	mice	vs placebo and Regranex	—
Rodgers et al., 2003	Four-arm topical experiment	$NorLeu^3$-angiotensin-(1–7)	100 μg/wound/day topically on days 0–4	5	mice	vs placebo and Regranex	—
Rodgers et al., 2003	Four-arm topical experiment	Regranex	100 μL becaplermin gel/wound/day topically on days 0–4; active concentration NR	5	mice	vs placebo and peptide arms	—

Table S1. Cont.

Study	Experiment/ comparison set	Experimental group/arm	Treatment regimen	n reported	Unit of n	Eligible comparison(s)	Notes
Brown et al., 1997	Growth-factor treatment arm	Diabetic vehicle	0.1 mL 5% PEG/PBS topically once daily on days 0–4	6/time point	mice	Vehicle comparator	—
Brown et al., 1997	Growth-factor treatment arm	rhIGF-II	10 μg rhIGF-II in 0.1 mL/wound/day on days 0–4	6/time point	mice	Single-GF comparator	—
Brown et al., 1997	Growth-factor treatment arm	rhPDGF-BB	10 μg rhPDGF-BB in 0.1 mL/wound/day on days 0–4	6/time point	mice	vs vehicle and GF combination	—
Brown et al., 1997	Growth-factor treatment arm	rhPDGF-BB + rhIGF-II	10 μg each GF in 0.1 mL/wound/day on days 0–4	6/time point	mice	vs vehicle and both monotherapies	Terminal cohorts days 5 and 10
Brown et al., 1997	Growth-factor treatment arm	Nondiabetic vehicle	Nondiabetic littermate + vehicle	6	mice	Contextual nondiabetic reference	Timing reported incompletely
Kiritsy et al., 1995	Experiment 1: combination vs low-dose PDGF	PDGF-BB + IGF-I	4 μg/cm² of each GF immediately and every other day through day 24	31	mice	vs PDGF-BB 4 μg/cm²	—
Kiritsy et al., 1995	Experiment 1: combination vs low-dose PDGF	PDGF-BB 4 μg/cm²	PDGF-BB 4 μg/cm² immediately and every other day through day 24	31	mice	vs two-GF combination	—
Kiritsy et al., 1995	Experiment 2: vehicle, high-dose PDGF, and combination	Vehicle	Methylcellulose vehicle immediately and every other day through day 24	12	mice	Vehicle comparator	—
Kiritsy et al., 1995	Experiment 2: vehicle, high-dose PDGF, and combination	PDGF-BB 40 μg/cm²	PDGF-BB 40 μg/cm² immediately and every other day through day 24	16	mice	vs vehicle and two-GF combination	—
Kiritsy et al., 1995	Experiment 2: vehicle, high-dose PDGF, and combination	PDGF-BB + IGF-I	4 μg/cm² of each GF immediately and every other day through day 24	14	mice	vs vehicle and high-dose PDGF	—
Brown et al., 1994	EGF combination experiment	Vehicle	0.1 mL 5% PEG topically once daily on days 0–4	8	mice	Vehicle comparator	—
Brown et al., 1994	EGF combination experiment	rhPDGF-BB	10 μg/wound/day on days 0–4	8	mice	vs vehicle and PDGF + EGF	—
Brown et al., 1994	EGF combination experiment	EGF	1 μg/wound/day on days 0–4	8	mice	vs vehicle and combination	—
Brown et al., 1994	EGF combination experiment	rhPDGF-BB + EGF	10 μg PDGF-BB + 1 μg EGF/wound/day on days 0–4	8	mice	vs vehicle and both monotherapies	—
Brown et al., 1994	TGF-α combination experiments	Vehicle	0.1 mL 5% PEG topically once daily on days 0–4	8–10/replicate	mice	Vehicle comparator	—
Brown et al., 1994	TGF-α combination experiments	rhPDGF-BB	10 μg/wound/day on days 0–4	8–10/replicate	mice	vs vehicle and PDGF + TGF-α	—
Brown et al., 1994	TGF-α combination experiments	TGF-α	1 μg/wound/day on days 0–4	8–10/replicate	mice	vs vehicle and combination	—
Brown et al., 1994	TGF-α combination experiments	rhPDGF-BB + TGF-α	10 μg PDGF-BB + 1 μg TGF-α/wound/day on days 0–4	8–10/replicate	mice	vs vehicle and both monotherapies	Experiments repeated 2–3 times; pooled closure analysis n=36/group

Table S1. Cont.

Study	Experiment/ comparison set	Experimental group/arm	Treatment regimen	n reported	Unit of n	Eligible comparison(s)	Notes
Greenhalgh et al., 1990	Five-day PDGF dose experiment; day-10 assessment	Vehicle	0.1 mL 5% PEG/PBS daily on days 0–4	20	mice	Vehicle comparator	—
Greenhalgh et al., 1990	Five-day PDGF dose experiment; day-10 assessment	rPDGF-BB 1 μg	1 μg/wound/day on days 0–4	13	mice	vs vehicle and 10-μg dose	—
Greenhalgh et al., 1990	Five-day PDGF dose experiment; day-10 assessment	rPDGF-BB 10 μg	10 μg/wound/day on days 0–4	8	mice	vs vehicle and 1-μg dose	—
Greenhalgh et al., 1990	Five-day PDGF/FGF experiment; day-10 assessment	Vehicle	0.1 mL 5% PEG/PBS daily on days 0–4	5	mice	Vehicle comparator	—
Greenhalgh et al., 1990	Five-day PDGF/FGF experiment; day-10 assessment	rPDGF-BB	10 μg/wound/day on days 0–4	6	mice	vs vehicle, rbFGF, and combination	—
Greenhalgh et al., 1990	Five-day PDGF/FGF experiment; day-10 assessment	rbFGF	1 μg/wound/day on days 0–4	5	mice	vs vehicle and combination	—
Greenhalgh et al., 1990	Five-day PDGF/FGF experiment; day-10 assessment	rPDGF-BB + rbFGF	10 μg PDGF-BB + 1 μg rbFGF/wound/day on days 0–4	6	mice	vs vehicle and both monotherapies	—
Greenhalgh et al., 1990	Five- vs 10-day PDGF duration experiment; day-10 assessment	Vehicle ×5 days	0.1 mL vehicle daily on days 0–4	6	mice	Matched duration comparator	—
Greenhalgh et al., 1990	Five- vs 10-day PDGF duration experiment; day-10 assessment	Vehicle ×10 days	0.1 mL vehicle daily on days 0–9	4	mice	Matched duration comparator	—
Greenhalgh et al., 1990	Five- vs 10-day PDGF duration experiment; day-10 assessment	rPDGF-BB ×5 days	10 μg/wound/day on days 0–4	10	mice	vs vehicle ×5 and PDGF ×10	—
Greenhalgh et al., 1990	Five- vs 10-day PDGF duration experiment; day-10 assessment	rPDGF-BB ×10 days	10 μg/wound/day on days 0–9	5	mice	vs vehicle ×10 and PDGF ×5	—
Greenhalgh et al., 1990	Five-day PDGF/FGF experiment; day-21 assessment	Vehicle	0.1 mL 5% PEG/PBS daily on days 0–4	17	mice	Vehicle comparator	—
Greenhalgh et al., 1990	Five-day PDGF/FGF experiment; day-21 assessment	rPDGF-BB	10 μg/wound/day on days 0–4	9	mice	vs vehicle, rbFGF, and combination	—
Greenhalgh et al., 1990	Five-day PDGF/FGF experiment; day-21 assessment	rbFGF	1 μg/wound/day on days 0–4	16	mice	vs vehicle and combination	—
Greenhalgh et al., 1990	Five-day PDGF/FGF experiment; day-21 assessment	rPDGF-BB + rbFGF	10 μg PDGF-BB + 1 μg rbFGF/wound/day on days 0–4	8	mice	vs vehicle and both monotherapies	—
Greenhalgh et al., 1990	Ten-day PDGF dose experiment; day-21 assessment	Vehicle	0.1 mL 5% PEG/PBS daily on days 0–9	5	mice	Vehicle comparator	—
Greenhalgh et al., 1990	Ten-day PDGF dose experiment; day-21 assessment	rPDGF-BB 1 μg	1 μg/wound/day on days 0–9	6	mice	vs vehicle and 10-μg dose	—
Greenhalgh et al., 1990	Ten-day PDGF dose experiment; day-21 assessment	rPDGF-BB 10 μg	10 μg/wound/day on days 0–9	7	mice	vs vehicle and 1-μg dose	—

Note: The table presents 160 tabulated experimental entries required to reconstruct every eligible rhPDGF-BB/becaplermin comparison; unrelated experiments within the same article that contained no rhPDGF-BB arm are omitted. Reported n values are retained in their original unit and were not converted into unique-animal totals when reported by wound, time point, experiment, or terminal cohort. Dose information was likewise retained as reported, and active rhPDGF-BB mass per wound was not inferred when the formulation amount, concentration, device yield, or other information required for a valid conversion was unavailable. AG, aminated β-1,3-D-glucan; AMD3100, plerixafor; AREG, amphiregulin; CMC, carboxymethylcellulose; CW, control wound; D, effect directly attributable to rhPDGF-BB because a no-factor or otherwise equivalent comparator was available; DW, diabetic wound; EGF, epidermal growth factor; F, effect attributable only to a multicomponent formulation containing rhPDGF-BB; FGF, fibroblast growth factor; GF, growth factor; HB-EGF, heparin-binding EGF-like growth factor; HSA, human serum albumin; Hsp90α, heat-shock protein 90 alpha; IGF, insulin-like growth factor; i.p., intraperitoneal; LAMA3, laminin subunit alpha-3; Na-CMC, sodium carboxymethylcellulose; NOD, non-obese diabetic; NOR, non-obese resistant; NR, not reported; PBS, phosphate-buffered saline; PDGF, platelet-derived growth factor; PEG, polyethylene glycol; PEI, polyethyleneimine; PlGF, placental growth factor; PLGA, poly(lactic-co-glycolic acid); PTS, pterostilbene; rbFGF, recombinant basic fibroblast growth factor; rh, recombinant human; SH, silk-sericin hydrogel; TGF, transforming growth factor; VEGF, vascular endothelial growth factor; WT, wild type.
